# A systematic literature review on mammography: deep learning techniques for breast cancer detection with global and Asian perspectives

**DOI:** 10.1186/s12885-025-14876-5

**Published:** 2025-10-22

**Authors:** Ashwini Amin, Dinesh Acharya U, Prakashini Koteshwara, Siddalingaswamy P C, Stanley Mathew

**Affiliations:** 1 Manipal Institute of Technology, Manipal Academy of Higher Education, Manipal, Karnataka 576104 India; 2 Department of Radio Diagnosis, Kasturba Medical Hospital, Manipal Academy of Higher Education, Manipal, Karnataka 576104 India; 3 Department of Surgery, Kasturba Medical Hospital, Manipal Academy of Higher Education, Manipal, Karnataka 576104 India

**Keywords:** Artificial intelligence, Breast cancer, Deep learning, Mammograms, Systematic literature review, Asian, Mammography, Global trend

## Abstract

**Purpose:**

Breast cancer remains a leading cause of mortality in women worldwide, with notable disparities in incidence and prognosis across regions. This systematic review explores the application of Deep Learning-based computer-aided diagnostic (CAD) systems for breast cancer detection, with a special focus on Asia to highlight underrepresented perspectives and challenges.

**Methods:**

We conducted a systematic Literature review in accordance with PRISMA guidelines. A comprehensive search of Scopus and Web of Science databases was performed to identify relevant studies published between January 2018 and November 2023, with an additional hand search for recent studies from 2024 to 2025. After screening 1051 records, 287 articles were included based on predefined inclusion and exclusion criteria. Quality assessment focused on the relevance of deep learning-based approaches to mammographic breast cancer detection, emphasizing global research trends and focused analysis of studies involving Asian populations.

**Results:**

The review identified major research trends in deep learning-based mammographic analysis, with most studies focusing on lesion classification while comparatively fewer addressed detection, segmentation, and breast density assessment. Studies using Asian datasets revealed unique challenges, including higher breast density, limited annotations, and under-representation in public datasets. Analysis of methodologies highlighted varied use of image preprocessing and augmentation techniques. Focus maps were used to visualize contributions across tasks and populations, revealing gaps in multi-class BI-RADS classification and a global research bias toward Caucasian datasets (*>* 80%).

**Conclusion:**

This review reveals that most deep learning models for breast cancer detection are trained predominantly on Caucasian datasets, creating significant limitations when applied to other populations due to demographic differences in breast density and imaging characteristics. To improve breast cancer screening globally, researchers must develop deep learning systems using diverse datasets that represent different populations, validate these models across various ethnic groups, and ensure clinical testing includes women from multiple demographic backgrounds.

**Systematic review registration:**

PROSPERO CRD 42,023,478,896.

**Supplementary Information:**

The online version contains supplementary material available at 10.1186/s12885-025-14876-5.

## Introduction

Breast cancer (BC) is a growing global health concern, with its incidence rapidly increasing and surpassing lung and thyroid cancers as the most prevalent malignancy worldwide, according to GLOBOCAN 2022 [1, 2]. In Asia, BC presents unique challenges, including an earlier onset, approximately a decade sooner than in Western countries, and a higher proportion of diagnoses at advanced stages [3]. This earlier onset is particularly significant due to the characteristic higher breast density in Asian populations, which complicates mammographic interpretation and contributes to delayed diagnoses, inadequate treatment, and early recurrence, ultimately resulting in lower survival rates compared to Caucasian, African, and Oceania populations [4–7].

Mammography is the standard screening tool for early BC detection globally, yet its efficacy is compromised in Asian populations due to higher breast density in younger women, which obscures tumours and reduces image clarity [8, 9]. This limitation increases both false-negative and false-positive rates. Recent advances in Artificial Intelligence (AI) have shown promise in enhancing mammographic sensitivity by providing comprehensive image analysis, reducing human error, and improving diagnostic accuracy [10–12]. However, cultural, socioeconomic, and infrastructural barriers such as limited awareness, stigma, inadequate access to screening, and the scarcity of large, annotated datasets further complicate early detection efforts in Asia.

BC incidence in Asia varies significantly across the continent and remains lower than in Western countries, yet the peak incidence occurs in the pre-menopausal age, with a median of 50 years in most Asian countries. Despite the widespread use of BIRADS in clinical practice, assigning accurate categories to mammograms with dense breast tissue is time-consuming and error-prone, leading to high inter-observer variability. Therefore, there is an unmet demand for AI-driven solutions that not only enhance the accuracy of BC detection but also address the unique challenges faced in the Asian context. Given these challenges, this Systematic Literature Review (SLR) aims to provide an in-depth analysis of deep learning (DL)-based Computer-aided Detection (CAD) systems for BC detection globally, with a particular focus on the Asian context.

By consolidating recent advances in BC diagnosis, this review aims to capture the rapid evolution of DL technologies and their application in BC detection. These seven years have witnessed significant breakthroughs in AI, particularly with the rise of sophisticated DL architectures such as Convolutional Neural Networks (CNNs), transformers, and hybrid models that have drastically improved diagnostic accuracy. Focusing on this period allows us to highlight cutting-edge techniques that reflect the current state of research, bridging the gap between traditional CAD approaches and modern AI-driven solutions. This review not only synthesizes global developments but also emphasizes the unique challenges faced in the Asian context, thereby guiding both researchers and practitioners toward more effective, region-specific diagnostic strategies.

## Primer on breast cancer and diagnostic approaches

### About breast cancer

BC is a heterogeneous disease that arises from various breast tissues, primarily ducts and lobules, as shown in the Supplementary Fig. 1, and is staged using the TNM system [13]. While malignant tumours pose serious health risks, many breast abnormalities such as fibroadenomas and cysts are benign but can mimic malignancies on imaging, making accurate diagnosis challenging. Rare breast cancers as shown in the Supplementary Fig. 2 can arise in the nipple (Paget’s disease), connective tissue (phyllodes tumours), or blood vessels (angiosarcoma). Most breast lumps, however, are benign, such as fibroadenomas and cysts common in younger and middle-aged women. Breast density is a critical factor in BC screening, as dense tissue not only increases cancer risk but also obscures lesions in mammograms. The American College of Radiologists classifies density into four categories, from almost entirely fatty to extremely dense as shown in the Supplementary Fig. 3. High breast density, more common in Asian populations, significantly reduces the sensitivity of mammography. Another important imaging marker is calcification. Breast calcifications appear as white dots on mammograms, with distribution patterns (Supplementary Fig. 4) helping determine risk - benign patterns (Supplementary Fig. 5) versus malignant patterns (Supplementary Fig. 6). Lesion characteristics, including proliferative potential lesions (Supplementary Fig. 7), provide crucial diagnostic indicators with shape and margins determining malignancy risk.

### Global epidemiology and regional disparities

GLOBOCAN 2022 [14] data reveal BC as the top cancer in 159 countries worldwide (Supplementary Fig. 8 A) with 15.4% mortality rate globally, and BC-related deaths leading in 111 countries (Supplementary Fig. 8B). Current burden distribution shows China with the highest rates (15.6% incidence, 14.8% mortality), followed by the USA (12% incidence, 11.3% mortality) and India (8.4% incidence, 6.4% mortality) (Supplementary Fig. 9). This data implies Asian countries are most affected compared to Western nations. Despite the USA’s high incidence ranking, its mortality rate remains lower than India’s due to superior healthcare infrastructure and early detection capabilities. Within Asia, rates vary significantly: East Asia and the Pacific regions show the lowest rates while South Asia demonstrates the highest burden-approximately twice that of East Asia-Pacific sub-regions.

BC presents differently between Western and Asian populations, with notable cultural and epidemiological variations. Western women typically develop BC between the ages 60–70, while Asian women face an earlier onset (40–50 years) with more aggressive forms. In Asia, cultural factors Like social norms and modesty issues often impede breast health awareness and screening. Early detection rates vary dramatically; with 60–70% of US cases diagnosed at stage 1 compared to only 1–8% in India. Risk factors fall into two categories: modifiable factors include physical inactivity, night shift work, post-menopausal obesity, delayed first pregnancy (after age 30), lack of breastfeeding, alcohol consumption, and hormone replacement therapy; non-modifiable factors include age, genetic predisposition (BRCA1/BRCA2, HER2), early menarche (before age 12), late menopause (after age 55), breast density, and family history.

**Fig. 1:**
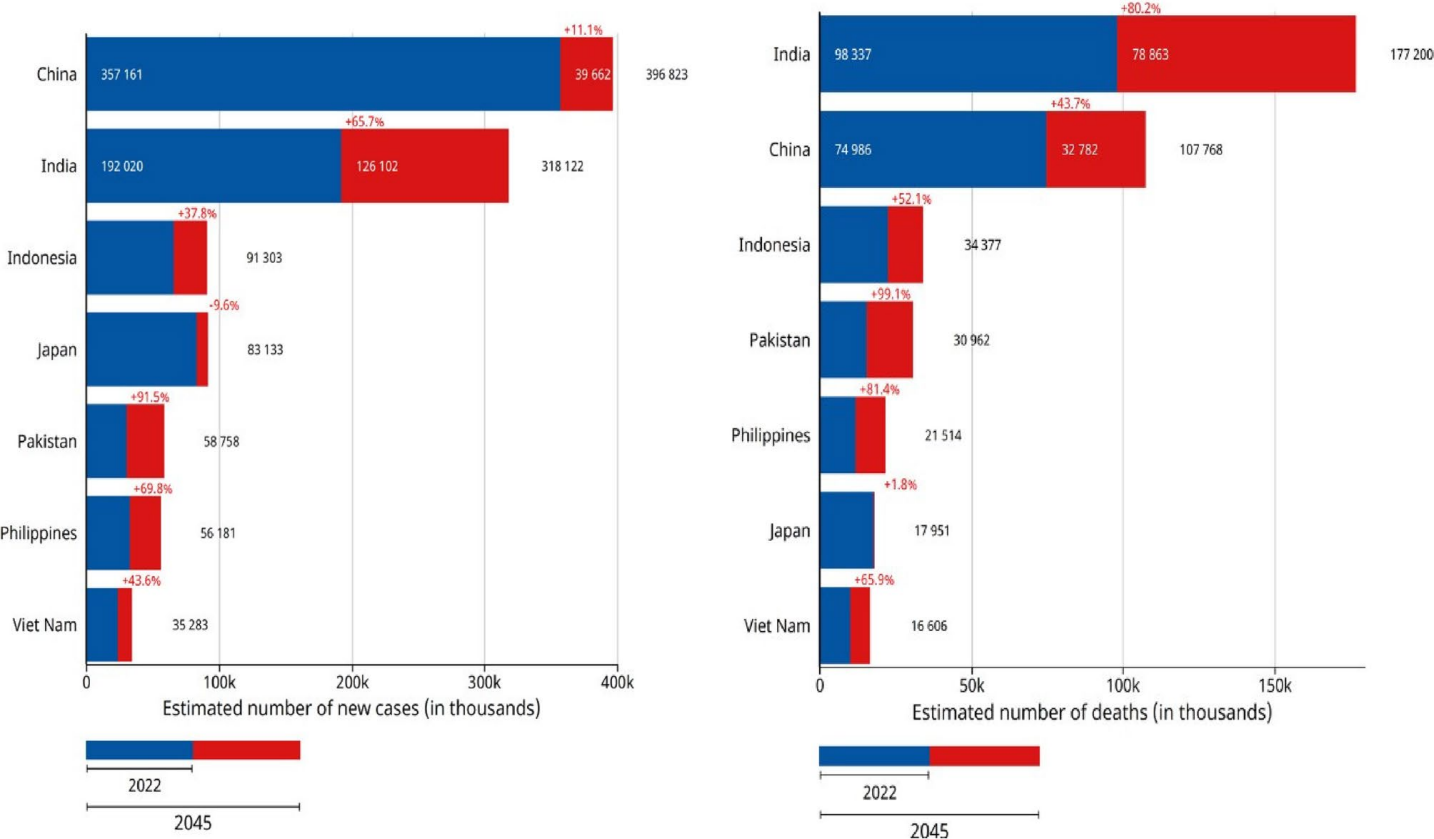
Estimated incidence and deaths from 2022 to 2040 [2]

Future projections suggest a dramatic increase in BC burden by 2040, with expectations of 3 million cases and 1 million deaths annually. The growth patterns vary significantly by development status, with low HDI countries facing an 80.6% increase, medium HDI countries (including India) 54.7%, high HDI countries (including China) 25%, and very high HDI countries (including the USA) 16.1% as shown in the Supplementary Fig. 10. The Fig. 1 chart shows projected breast cancer deaths across Asian countries from 2022 to 2045, with India facing the most dramatic increase of 80.2%. Developing Asian nations show alarming growth rates, with Pakistan (+ 99.1%), Philippines (+ 81.4%), and Indonesia (+ 52.1%) all experiencing substantial increases.

Japan shows minimal growth (+ 1.8%) due to its advanced healthcare system, while China, despite a lower percentage growth (+ 43.7%), will still see significant absolute increases.

### Mammography and breast cancer screening with deep learning based diagnosis approaches

Digital Mammography (DM) remains the gold standard for early BC detection, utilizing X-ray imaging in two standard views: mediolateral oblique (MLO, 45^◦^ angle) and cranio-caudal (CC, horizontal 0^◦^ angle). The Breast Imaging-Reporting and.

**Fig. 2:**
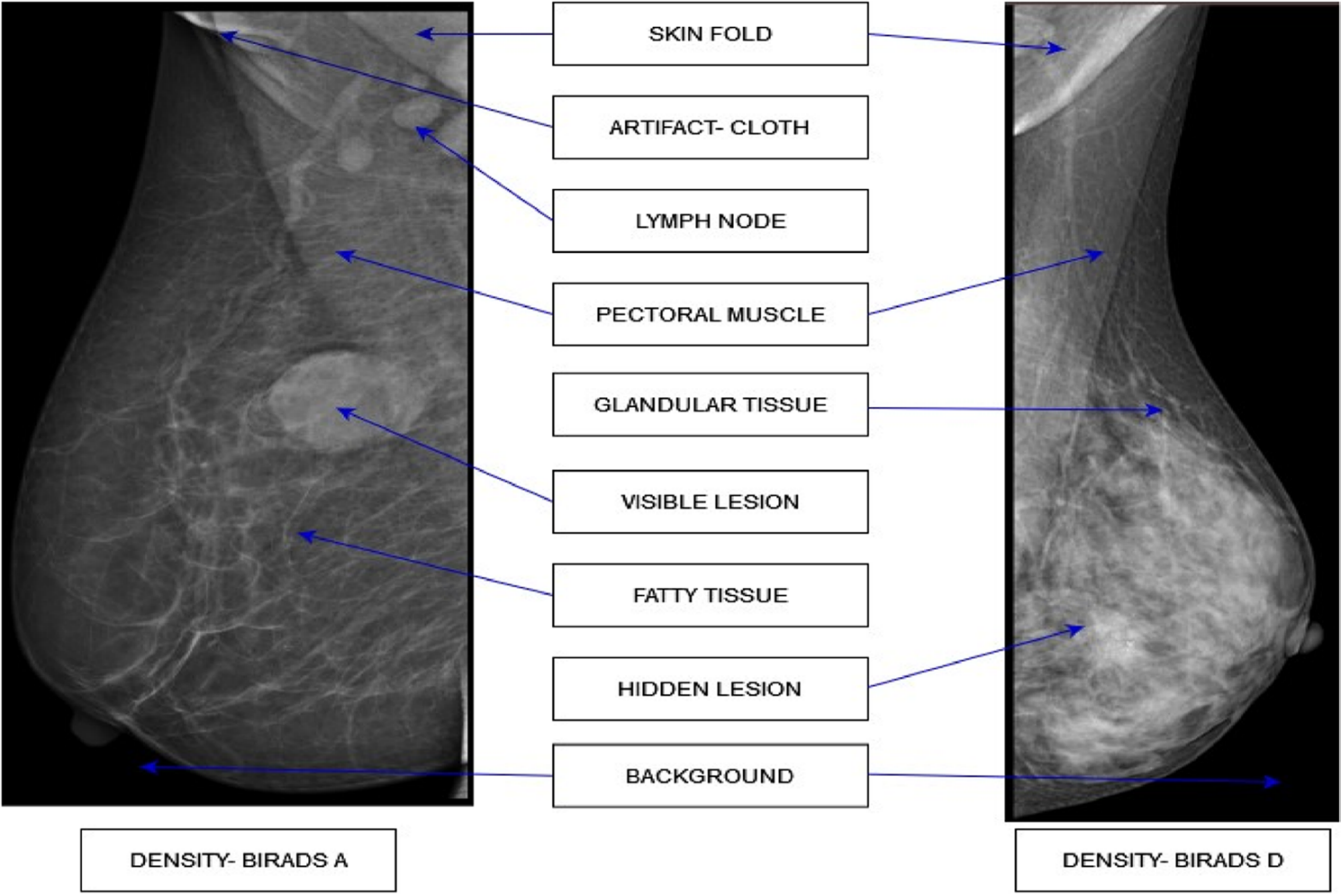
Various elements of the Digital Mammogram image belonging to Vin-Dr Mammo dataset [15]

Data System (BI-RADS) provides standardized classification ranging from category 0 (incomplete) to 6 (known malignancy) (Supplementary Table S1), while also categorizing breast density from Type A (almost entirely fatty) to Type D (extremely dense) (Supplementary Table S2). While DM offers advantages like superior resolution and quick imaging time, it presents challenges for Asian women who typically have denser breast tissue, potentially leading to false positives or missed diagnoses. The various elements of DM, as seen in the low-density breast (BI-RADS A) and high-density breast (BI-RADS D), are shown in Fig. 2. The evolution of technology from Screen Film Mammograms (SFM) to Full-Field Digital Mammograms (FFDM) has improved visualization, especially for dense breasts. Recent advances in artificial intelligence, particularly DL and CNNs, have revolutionized mammogram analysis. From early applications in the 1960 s to modern sophisticated networks like AlexNet, VGGNet, and EfficientNet, these technologies have progressed to include advanced techniques such as attention-based CNNs, Generative Adversarial Networks, and Visual Transformers, offering promising improvements in BC detection accuracy and early diagnosis.

## Methods

### Research goals

This SLR addresses the following research questions:

- **RQ1**: What are the current methodologies and advancements in DL-based BC and density detection, segmentation, and classification using mammograms into various.

categories of BI-RADS most extensively researched in the literature in Asia and the rest of the world?

- **RQ2**: What are the unique challenges and considerations in applying DL techniques to BC detection using mammography in the Asian population?
- **RQ3**: What are the most common mammogram datasets used in the literature, and why do Asian data need to be analyzed?
- **RQ4**: What is the potential impact of pre-processing and augmentation techniques on the robustness of DL models?
- **RQ5**: What are the key drawbacks and future directions that should be explored to enhance precise and faster assessment and improve diagnostic accuracy?

By exploring these areas, this review identifies gaps and offers insights to advance BC detection methods globally, particularly for Asian populations.

### Protocol registration and reporting guidelines

The protocol for this systematic literature review was prospectively registered in PROSPERO (CRD42023478896) to ensure methodological transparency. This review strictly follows the PRISMA (Preferred Reporting Items for Systematic Reviews and Meta-Analyses) guidelines [16] for systematic reviews, with appropriate adaptations for deep learning and technical research studies. The PRISMA framework guided our approach to article selection criteria, inclusion/exclusion parameters, study selection process, data extraction, quality assessment, and result reporting. A complete.

**Fig. 3:**
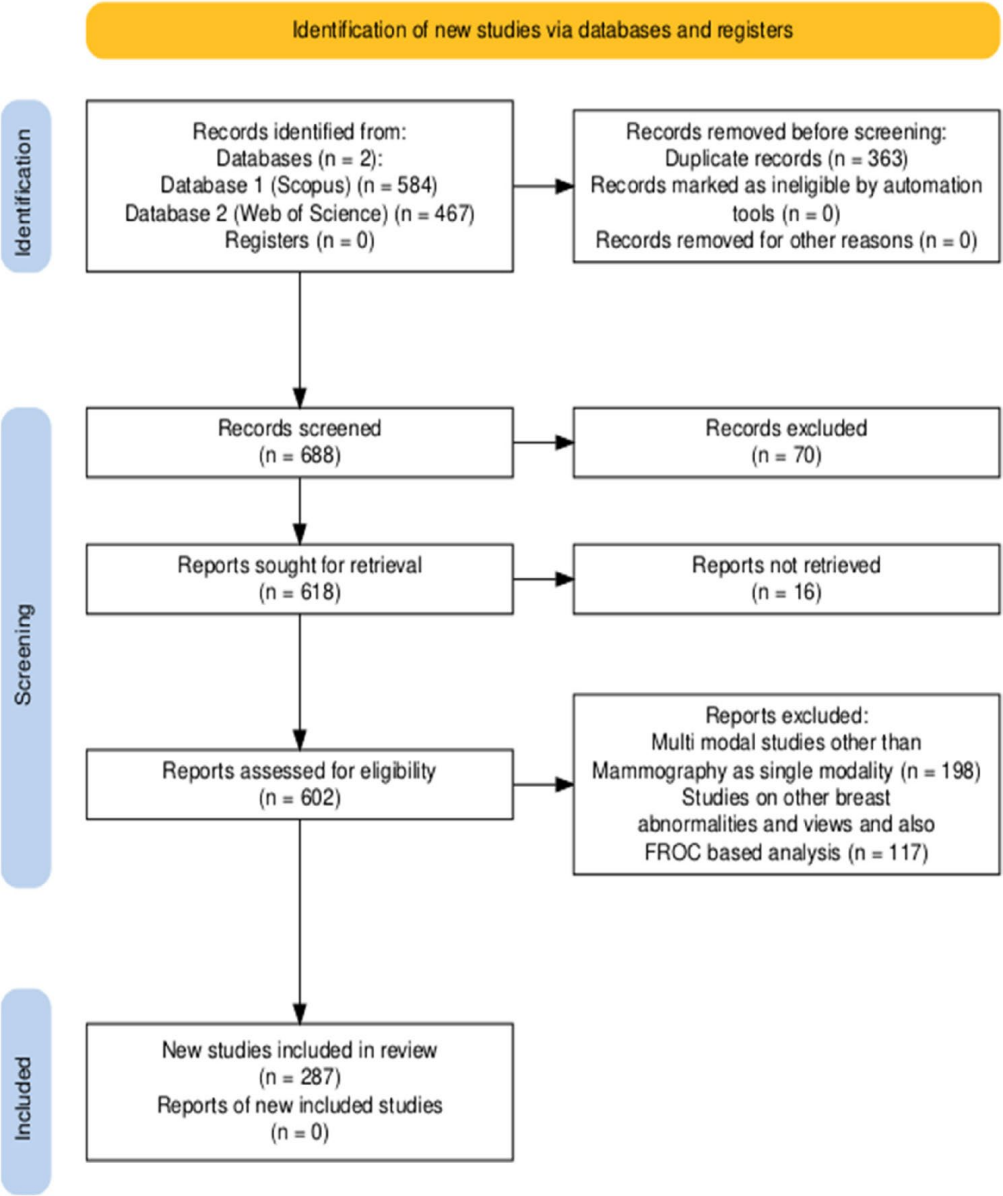
PRISMA flowchart of the proposed SLR

PRISMA checklist was followed throughout the review process, and the study selection workflow is presented in the PRISMA flowchart (Fig. 3). This approach ensures comprehensive and transparent reporting of our systematic review methodology and findings.

### Selection criteria

A comprehensive review was conducted to analyze recent advancements (2018–2023) in DL-based BC detection, segmentation, and classification using mammograms. Relevant studies were retrieved from Scopus and Web of Science using a custom query (Table S1). The timeframe was selected as it captures the evolution from CNNs to advanced architectures like transformers, driving significant advancements in BC diagnosis using mammography. The search process involved applying inclusion/exclusion criteria, duplicate removal, and full-text screening. Tools like JabRef [17] for reference management and VOSviewer for visualization were used. The term ’Asian’ was not included in the search strings because studies often mention specific dataset names or hospital names rather than demographic terms, and this approach allowed us to capture all relevant studies for our global comparison while addressing region-specific challenges through research questions (RQ1-RQ5). The search for relevant papers was conducted using specific search strings and conditions on two databases, Scopus and Web of Science, with a focus on ”Breast Cancer,” ”mammography,” and ”Deep Learning” techniques for detection, segmentation, classification, or identification. The Scopus search resulted in 584 papers, and the Web of Science search yielded 467 results. In total, 1051 papers were extracted from both databases.

### Inclusion and exclusion criteria

Table 1 outlines the inclusion criteria, focusing on original research articles published between January 2018 and November 2023, involving women over 18, and utilizing mammography with DL models for detection, segmentation, feature extraction, and classification. Studies had to report empirical performance metrics, specifically using CC and MLO mammogram views, and be published in English. Exclusions applied to studies involving other populations, imaging modalities, non-empirical data, non-journal articles, and non-English publications. The inclusion and exclusion criteria were strategically designed to ensure comprehensive coverage of our research questions through a two-tier approach. RQ1 (current DL methodologies and advancements) is directly addressed through IC2 (mammography modality), IC3 (DL models for detection/segmentation/classification), IC4 (empirical performance metrics), and IC6 (original research articles), while EC2 and EC4 ensure focus on mammography-specific deep learning approaches. RQ5 (drawbacks and future directions) is captured through IC6 and IC8 (articles answering research questions) combined with EC6 (excluding reviews and abstracts that lack empirical insights). RQ2 (Asian population challenges), RQ3 (mammogram datasets), and RQ4 (preprocessing impact) are addressed through secondary analysis during data extraction, as these specific aspects cannot be reliably identified through title and abstract screening alone. The broad inclusion criteria IC1-IC10 ensure comprehensive study capture while maintaining methodological rigor through IC4 (empirical evidence requirement) and IC5 (standardized mammogram views), enabling targeted analysis of population-specific challenges, dataset characteristics, and preprocessing methodologies during the data synthesis phase.

**Table 1:** Inclusion and Exclusion Criteria for Study Selection

**Criteria**	**Inclusion (IC)**	**Exclusion (EC)**
Population Type	IC1: Women aged *>*18 years	EC1: Studies involving men, animals, or children
Intervention	IC2: Mammography as a modality IC3: Use DL models for detection, segmentation, feature extraction, and classification	EC2: Studies related to other imaging modalities (e.g., MRI, CT, Tomosynthesis, ultrasound) EC3: Studies related to the assessment of CAD systems along with radiologist’s detection accuracy
Outcome	IC4: Studies reporting performance metrics supported by empirical evidence IC5: Study using mammography with only the CC and MLO views	EC4: Studies fail to report relevant diagnostic measures
Study Design	IC6: Original research articles IC7: Articles limited to journal and early-access publications IC8: Articles answering research questions	EC5: Clinical Trials, Case studies, Grey Literature EC6: Conference articles, Abstracts, and Reviews EC7: Articles with no information regarding research questions
Period	IC9: Articles published between January 2018 and 15 November 2023	EC8: Articles prior 2018 and after 15 November 2023
Language	IC10: All articles are written in English	EC9: Articles in non-English languages

### Quality assessment

Studies that met the initial selection criteria underwent detailed quality evaluation using JabRef software to assess their methodology, relevance to research objectives, and evidence reliability. Each article was systematically reviewed and annotated within JabRef, where detailed comments were added to evaluate key methodological aspects, including study design, dataset used, modalities, various deep learning methods used, and type of breast abnormalities studied. For relevance, studies using multiple imaging methods (histopathology, MRI, CT, digital breast tomosynthesis, contrast-enhanced mammography, or ultrasound) alongside mammography were excluded, regardless of their methodological quality. Studies that only performed statistical analysis (FROC) without implementing deep learning models for detection, classification, or segmentation were also removed. Additionally, studies using mammographic views other than standard CC and MLO positions, or those focusing on breast abnormalities beyond masses, calcifications and density, were eliminated to maintain focus on the specific research scope. The initial search yielded 1,051 articles (584 from Scopus, 467 from Web of Science). After removing 363 duplicates, 688 articles were screened.

Following screening, 70 records were excluded (67 review articles, 2 withdrawn, and 1 retracted paper), and 618 reports were sought for retrieval. However, 16 reports could not be retrieved, leaving 602 reports for eligibility assessment. Exclusions during eligibility assessment included 198 multimodality studies and 117 articles that failed abstract screening due to irrelevant content or failure to meet inclusion criteria. Ultimately, 287 articles met all criteria for inclusion in the qualitative synthesis (Fig. 3).

### Supplementary hand search approach

To capture recent developments in this area of study, supplementary hand searching was conducted from January 2024 to June 2025, monitoring high-impact journals. The same search string used in the selection of review articles for the systematic Literature review was employed for this approach; however, only the best articles were selected. This targeted lesion detection, segmentation, classification, and breast density analysis using deep learning. The hand search identified 25 relevant articles revealing trends in transformer architectures, multi-view analysis, multi-modal adaptations, and new datasets confirming the continued relevance of our systematic review findings and proposed research directions.

## Results and discussion

### Data extraction and data synthesis

Data extraction was conducted following the methodology outlined earlier, focusing on two key components: (i) general characteristics of the selected studies and (ii) a classification scheme.

#### General characteristics

**Fig. 4:**
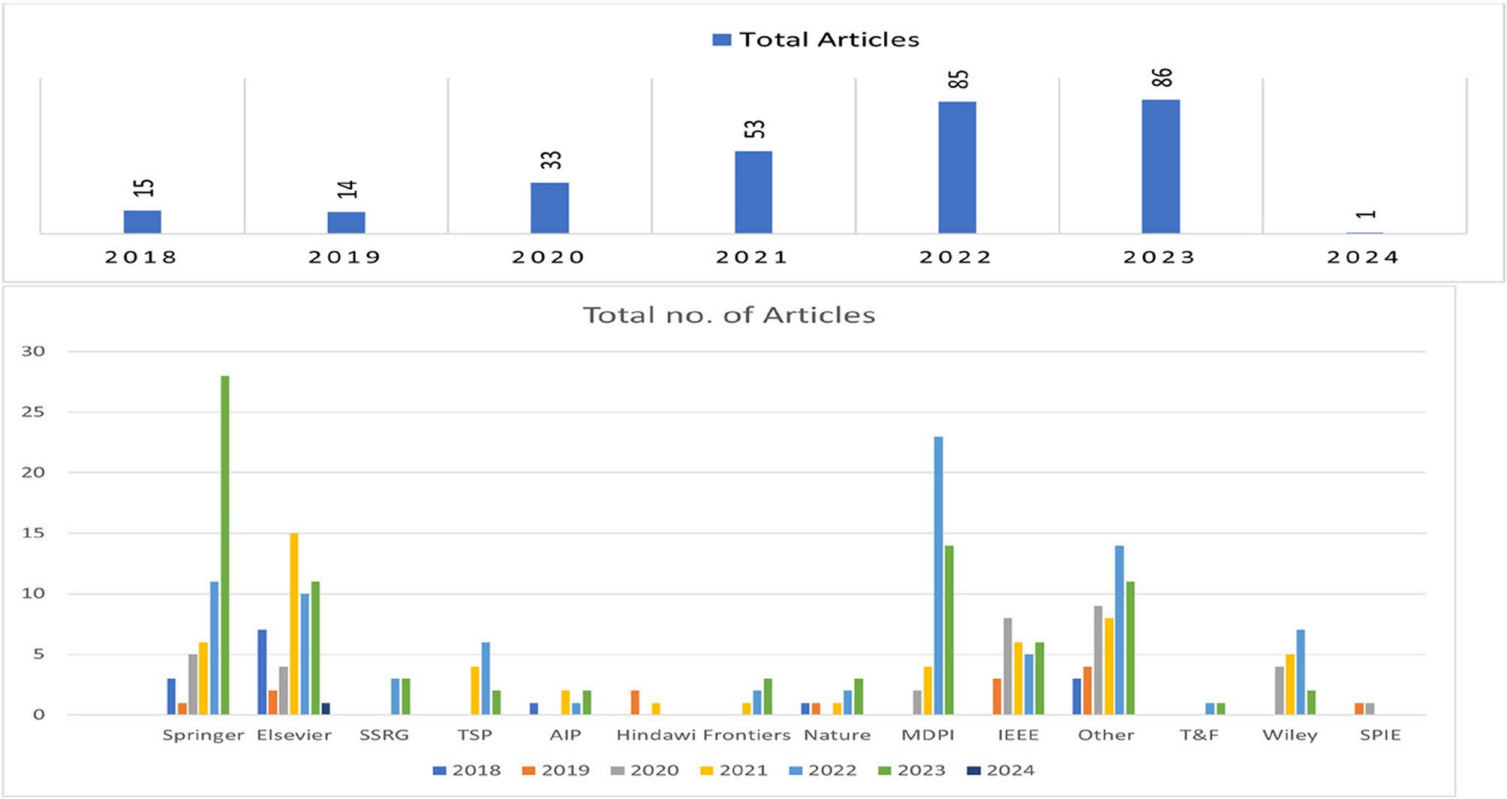
Publication Trend Over Five Years globally (left) and Publisher-wise contribution in publishing the articles over 5 years globally (right). Note: The 2024 entry (1 article) represents an article included based on early access criteria. SSRG (Seventh Sense Research Group), AIP (American Institute of Physics), Tech Science Press (Tech Science Press)

**Fig. 5:**
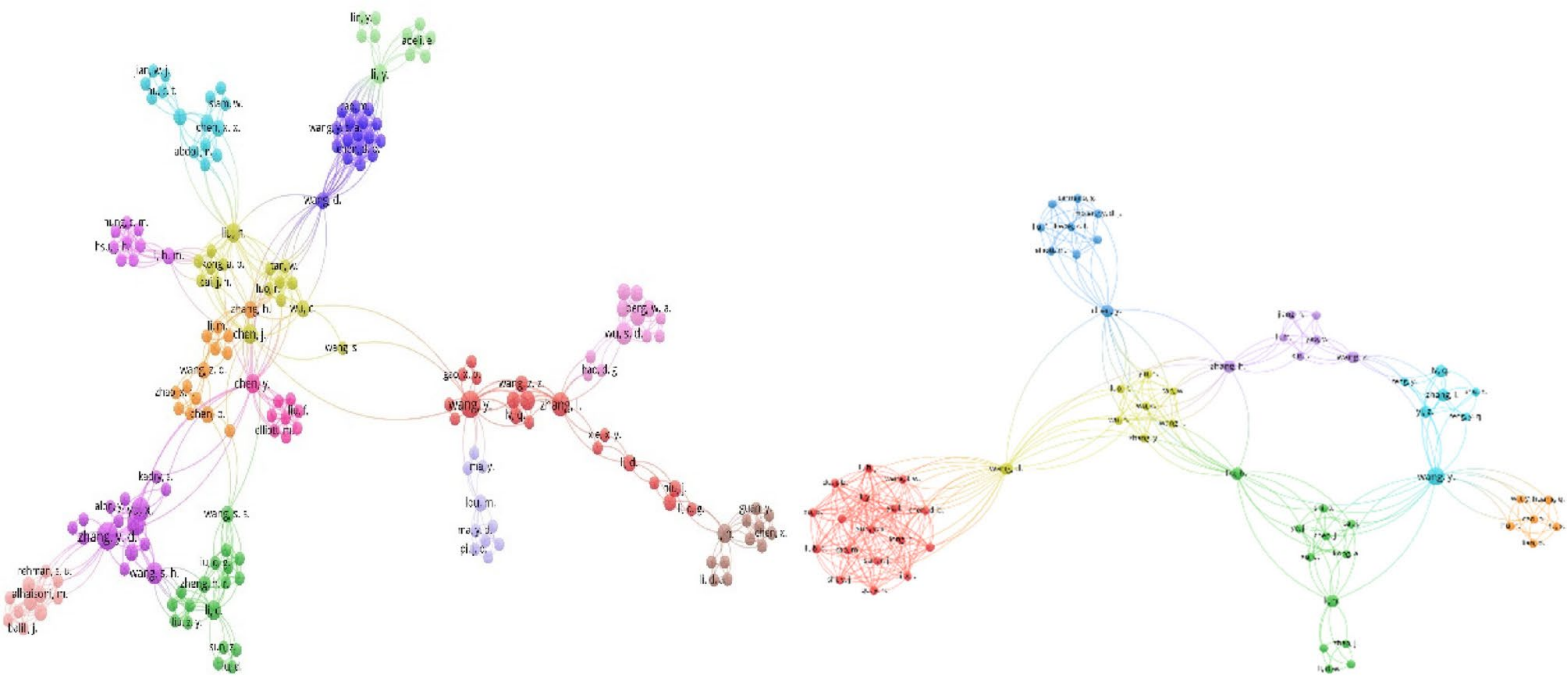
Visualization of Co-Author citation with weights based on the number of articles worldwide (left) and for Asian countries (right)

**Fig. 6:**
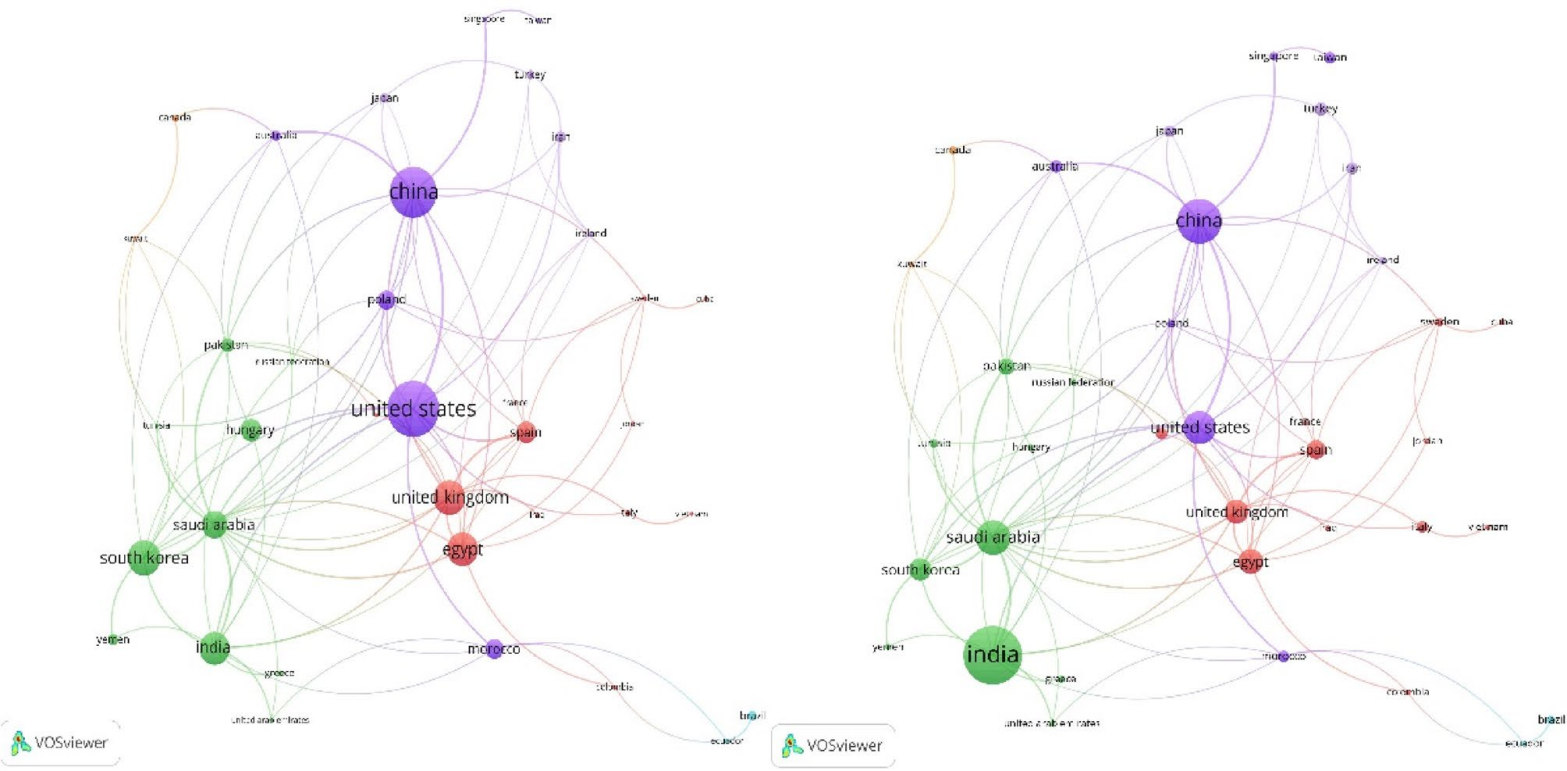
Visualization of country-wise citations with weights based on number of citations (left) and number of articles contributed (right)

**Fig. 7:**
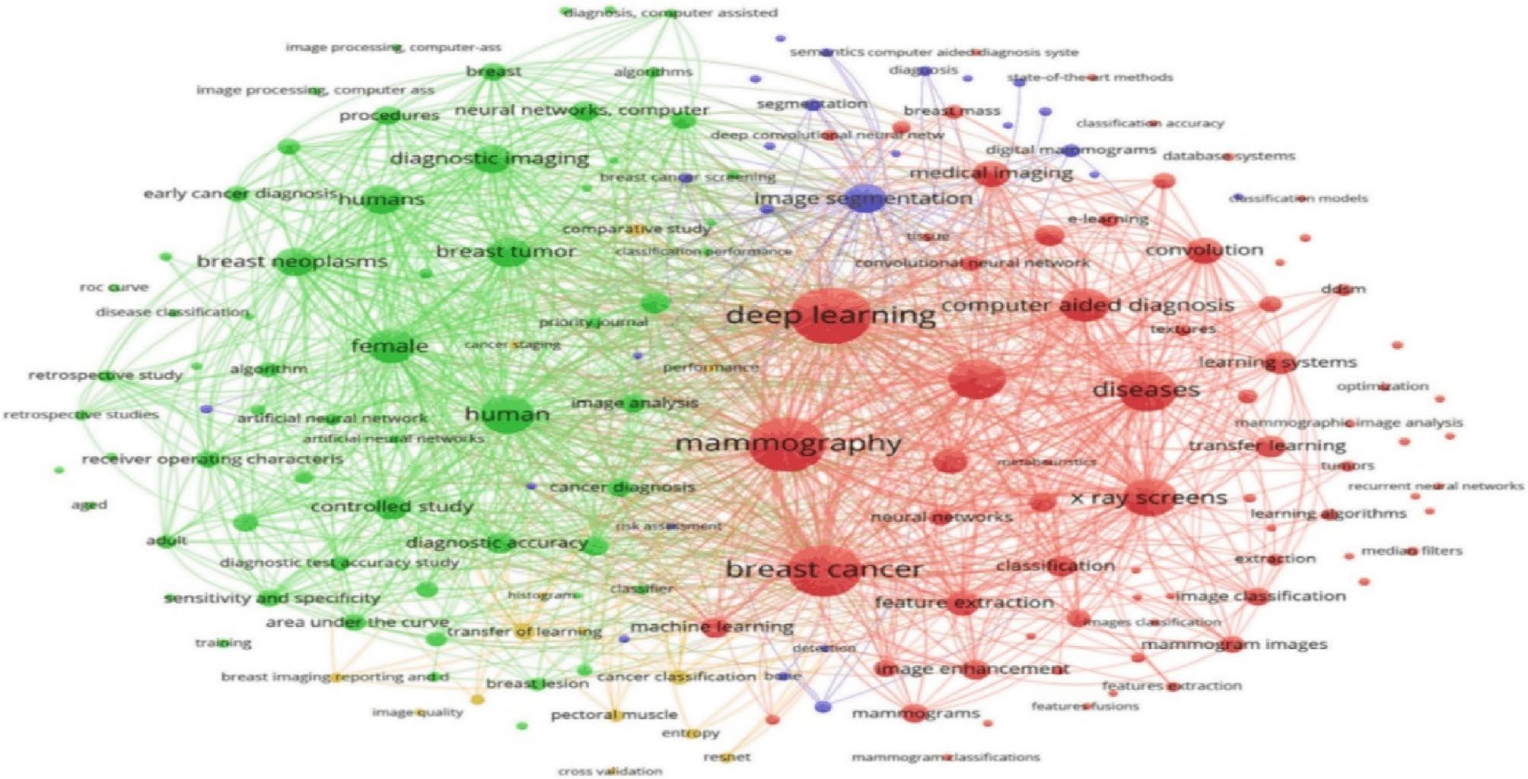
Visualization of frequent Keywords in the research field considered worldwide

This section addresses RQs 1 and 3, examining publication trends, key publishers, leading authors, contributing countries, and frequently used keywords. Visualization tools such as VOSViewer [18] and Microsoft Excel were employed for analysis. The temporal analysis (Fig. 4, left) shows exponential growth from 15 articles in 2018 to 86 in 2023—a six-fold increase indicating the field’s transition from nascent research to mainstream adoption. The acceleration after 2020 coincides with advances in deep learning architectures and increased computational accessibility. The publisher analysis (Fig. 4, right) reveals Springer and Elsevier as dominant traditional publishers, while MDPI’s rapid growth post-2021 reflects the field’s embrace of open-access publishing. This shift enhances research accessibility, particularly for developing countries expanding mammography infrastructure. Co-authorship analysis (Fig. 5) reveals fragmented collaboration patterns with distinct clusters, suggesting missed opportunities for knowledge transfer and methodological standardization. The separate Asian visualization highlights regional collaboration tendencies but indicates need for global collaborative frameworks. Country-wise analysis (Fig. 6) shows research concentration in Asia, North America, and Europe, raising questions about algorithmic generalizability across diverse populations. High-publication countries with lower citation impact suggest emerging research capabilities requiring international collaboration support. Keyword analysis (Fig. 7) demonstrates successful integration between technical methods (deep learning, neural networks) and clinical applications (breast cancer screening, mammography). The transitional terminology mix suggests methodological evolution from traditional to modern approaches, though limited implementation-focused keywords indicate gaps between research and practical adoption. These patterns reveal a technically robust but strategically challenged field requiring consortium-based initiatives for standardization, international partnerships for algorithmic fairness, and enhanced focus on clinical deployment to bridge the research-practice gap.

#### Classification scheme

**Fig. 8:**
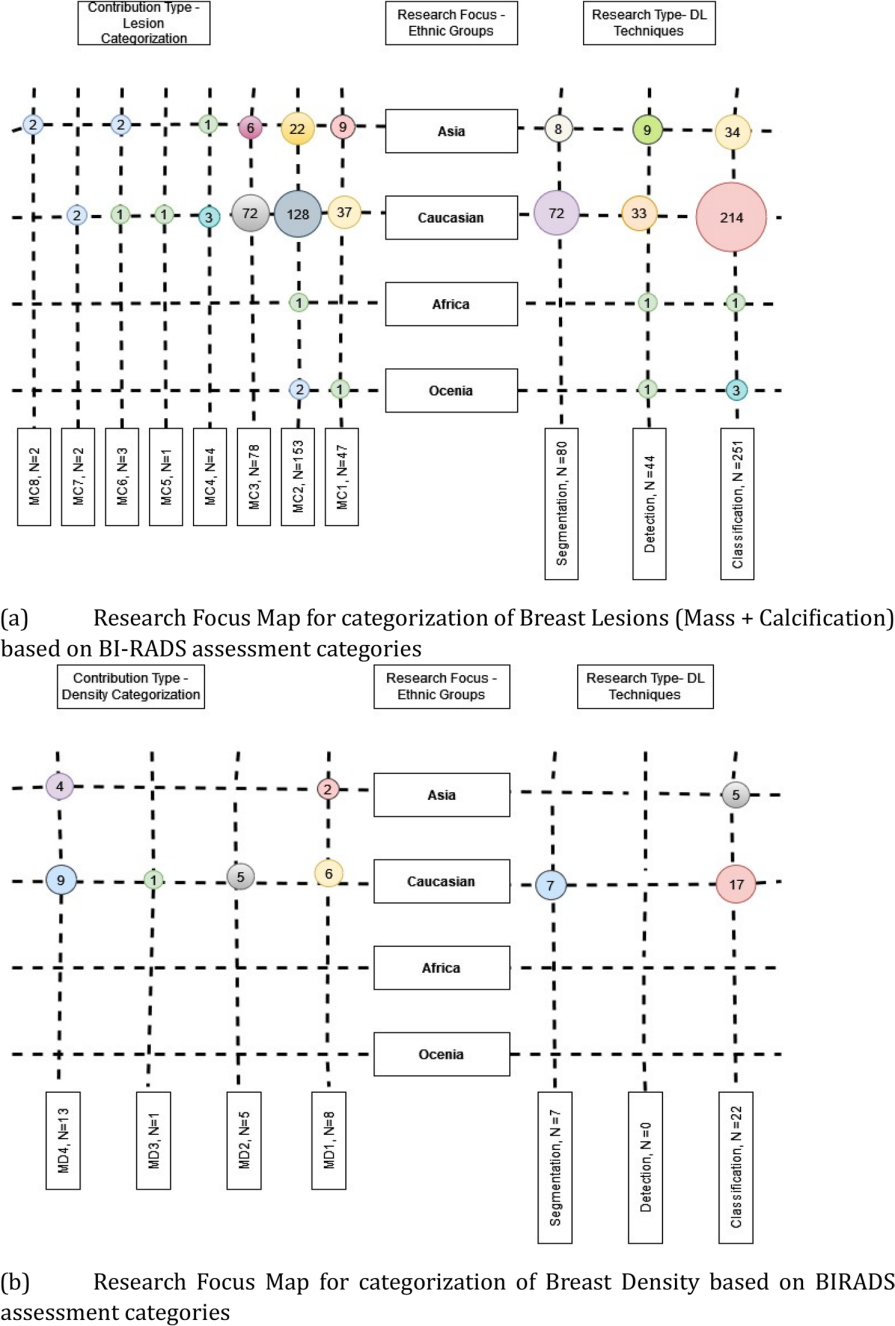
**a** Research Focus Map for Breast Lesions (Mass + Calcification) (**b**) Research Focus Map for Breast Density

A research focus map was created to show research types, focus areas, and contribution types for BI-RADS lesions Fig. 8a and breast density Fig. 8b. Datasets from America and Europe were grouped as Caucasian, while others were categorized as Asian, Oceanian, and African. The map aims to analyze past research and identify gaps for future studies.

The population-based disparities in Asian populations show Limited research coverage, with only 9 detection and 8 segmentation studies for lesions, compared to 72 detection and 33 segmentation studies in Caucasian populations. This represents an 8-fold difference in detection and a 4-fold difference in segmentation research that could impact clinical care quality. The BI-RADS category underrepresentation is concerning. Research heavily skews toward binary categorization (14% Asian, 84% Caucasian studies focus on benign vs. malignant, and 19% Asian, 79% Caucasian studies focus on Normal vs. Abnormal). Critically, very limited studies comprehensively classify all BI-RADS categories (MC8: 2 studies), and higher-complexity classifications (MC5MC7) remain underexplored, with fewer than 6 studies each across all populations. This represents a fundamental mismatch between clinical needs, where radiologists must distinguish between all BI-RADS categories.

Breast density research shows pronounced disparities. African and Oceanian populations have zero dedicated studies, while Asian populations have only 5 studies compared to 24 in Caucasian populations which is a 5-fold difference that may contribute to diagnostic disparities. The absence of research in African and Oceanian regions can be attributed to several factors: limited availability of annotated datasets, fewer researchers from these regions working on breast cancer AI, insufficient healthcare infrastructure for data collection, reduced funding for medical AI research [19–21]. The technical approach imbalances are evident. Detection and segmentation consistently receive less attention than classification, with segmentation particularly underrepresented. In dense Asian breasts, this segmentation research gap is clinically critical. Clinical implications are substantial. Models trained predominantly on Caucasian data may not perform accurately on Asian datasets due to ethnic differences.

in breast tissue density, anatomical structure, and imaging characteristics, leading to reduced performance in underrepresented populations. These findings highlight the critical need for focused research on underrepresented populations and improved coverage of comprehensive BI-RADS categorization to advance fair and clinically useful AI-driven breast cancer detection across diverse demographic groups.

#### Contribution type

**Fig. 9:**
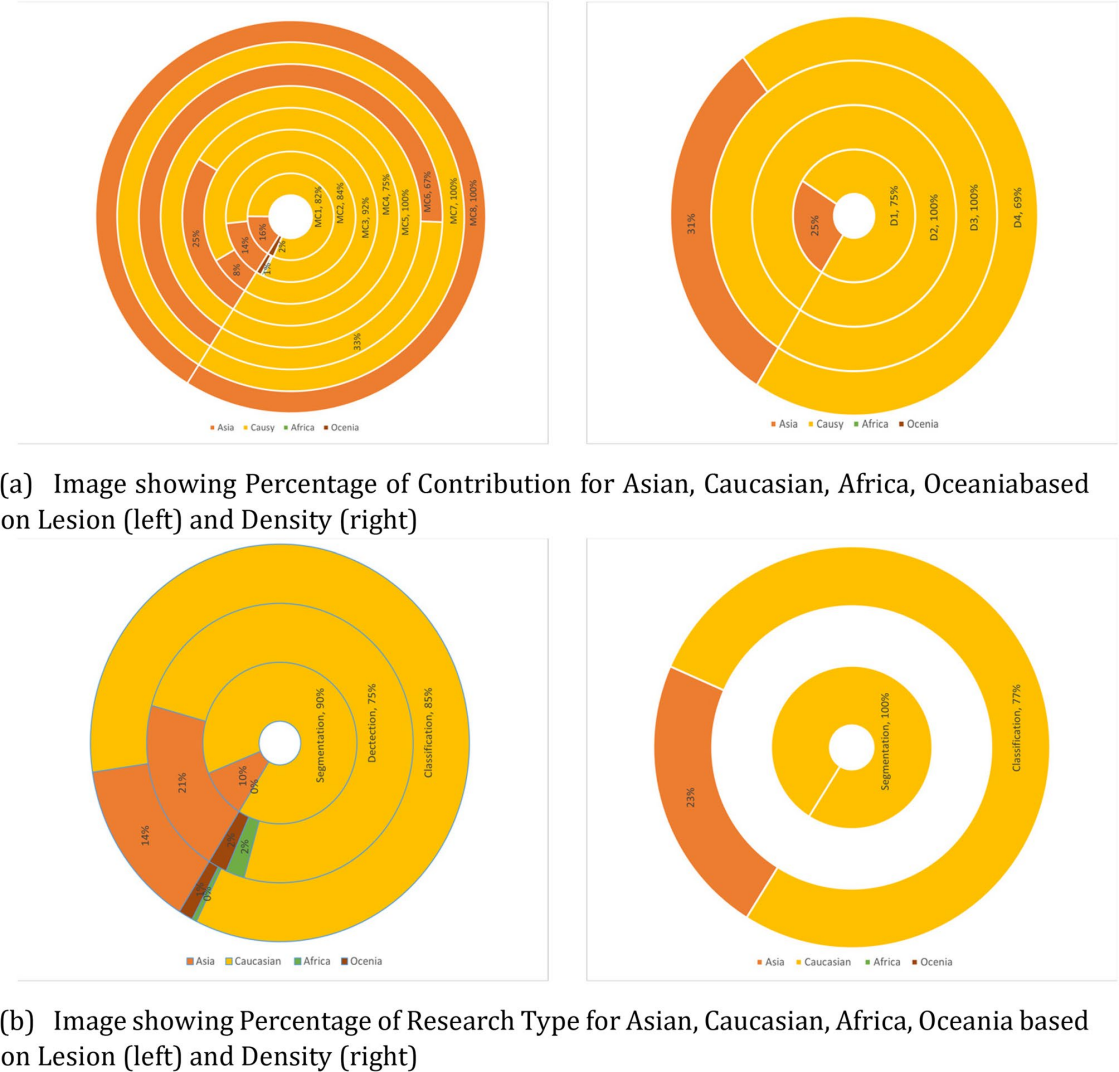
**a **Contribution Percentage based on Lesion and Density (**b**) Research Type Percentage based on Lesion and Density

In a research focus map, ”Contribution Type” defines the key contributions of a study. BI-RADS classes are categorized based on (i) breast density (D1-D4) and (ii) mass and calcifications (MC1-MC8). While mammograms assess multiple factors, this review focuses on breast density, calcifications, and mass, with a separate Focus Map clarifying these contributions (Table 2). Analysis of the Research Focus Map (Fig. 8a) and pie chart (Fig. 9a) indicates that most studies (55% Asian, 53% Caucasian) classify lesions as benign or malignant, with limited research covering all BI-RADS categories. No comprehensive studies classify all BI-RADS categories in Caucasian datasets, and research remains scarce in Africa and Oceania due to limited annotated datasets. Breast density classification is particularly underexplored in Asian datasets, highlighting the need for broader research.

#### Research type

”Research Type” categorizes studies based on their primary goal in a research focus map. This review considers quantitative research using DL-based methods for detection, segmentation, and classification tasks across breast imaging applications.

The three primary research types address distinct clinical needs: classification categorizes lesions or breast density into predefined classes; detection identifies and locates abnormalities; and segmentation provides precise delineation of regions of interest. The pie chart (Fig. 9b) reveals classification studies dominating across all population cohorts, reflecting the clinical priority of distinguishing benign from malignant findings.

However, the scarcity of detection and segmentation studies represents a significant gap, as these methodologies are crucial for automated screening systems. This limitation stems primarily from the lack of comprehensively annotated datasets for nonCaucasian populations. Detection and segmentation require pixel-level annotations and precise region-of-interest delineations, which are significantly more labor-intensive than image-level labels used in classification tasks.

Geographic disparities further compound these issues. Research on breast density remains scarce in Oceania and Africa, creating substantial knowledge gaps that limit the clinical applicability of existing models when deployed in underrepresented regions.

#### Research focus

In a research focus map, ”research focus” highlights key areas of interest within a domain and serves as a critical tool for identifying research gaps and opportunities for future investigation. In this review, our main focus is to analyze the distribution of deep learning studies across classification, detection, and segmentation tasks for different BI-RADS categories of lesions and breast density assessments, while examining the number of studies conducted across various population cohorts. Separate focus maps were created for density (Fig. 8b) and lesions (Fig. 8a).

Studies were categorized by cohort—Asian, Caucasian, African, or Oceanian—to analyze research distribution patterns. The maps reveal that classification, particularly distinguishing benign from malignant lesions, dominates research (53% of lesion classification studies), while segmentation and detection receive less attention.

Notably, more studies have been conducted on Caucasian datasets than Asian ones (Fig. 9b). The focus map also highlights significant research potential in African and Oceanian datasets. This disparity in research distribution significantly affects real-world generalizability and clinical deployment of models trained predominantly on Caucasian populations, potentially limiting their effectiveness across diverse global patient populations.

### RQ1: What are the current methodologies and advancements in DL-based BC and density detection, segmentation, and classification using mammograms into various categories of BI-RADS most extensively researched in the literature in Asia and the rest of the world?

DL has significantly advanced BC image analysis, evolving from traditional ML to CNNs, TL, and attention mechanisms, with recent adoption of vision transformers for classification, detection, and segmentation. Architectural trends show a clear evolution from traditional CNN-based approaches (predominantly ResNet, VGG, and DenseNet variants) to attention-based mechanisms and transformer architectures, with hybrid models combining convolutional and self-attention mechanisms gaining prominence in recent studies. The various techniques used are explained in the Tables. All Asian dataset studies are cited in red colour.

**Table 2:** Contribution Type Details

**Type**	**Categories**	**Description**	**Coding**
Density	Dense Vs Non dense	Based on if breast is dense or not	D1
	2 class	BI-RADS A vs D, B vs D, C vs D, B vs C	D2
	3 class	fatty vs dense vs glandular	D3
	4 class	BI-RADS - A, B, C, D	D4
Lesion (Mass+Calcification)	Normal Vs Abnormal	Based on if mass or calcifications are present or absent	MC1
	Benign Vs Malignant, Negative Vs Positive, Normal Vs Malignant, Normal Vs Positive, BI-RADS 3 Vs 4	BI-RADS {2,3}: benign and BI-RADS {4,5,6}: Malignant / BI-RADS {1,2,3}: Negative and BI-RADS {4,5,6}: Positive / BI-RADS {1,2}: normal and BI-RADS {3,4,5,6}: malignant / Normal and Benign: Benign and rest: Malignant / Normal and Benign: Normal and Malignant: Positive	MC2
	Normal Vs Benign Vs Malignant	BI-RADS {1}: Normal, BI-RADS {2,3}: Benign, and BI-RADS {4,5,6}: Malignant	MC3
	3 class	Benign Vs Benign without callback Vs Malignant / Normal Vs Minor Vs Severe (for calcification) / Calcification, Tumor, and Healthy	MC4
	4 class	BI-RADS {1,2,3,4} or Based on the severity of the calcifications	MC5
	5 class	BI-RADS {2,3,4,5,6} or Type of calcification - diffuse, segmental, regional, grouped, or linear	MC6
	6 class	BI-RADS {2,3,4A,4B,4C,5} or BI-RADS {1,2,3,4,5,6}	MC7
	8 class	BI-RADS {0,1,2,3,4A,4B,4C,5}	MC8

Breast lesion detection relies on DL models such as YOLO and Faster R-CNN, with newer transformer-based approaches; however, only 20% of studies focus on Asian datasets. This underrepresentation in Asian datasets stems from several factors: limited availability of large-scale annotated datasets, resource constraints for comprehensive labelling, regulatory barriers for cross-institutional data sharing, and varying imaging protocols across different healthcare systems. To address these limitations, future research should leverage transfer learning from Western datasets to Asian populations, implement federated learning frameworks that enable collaborative model development while preserving data privacy, and develop domain adaptation techniques that account for population-specific breast tissue characteristics and imaging variations. Methods utilized on Asian datasets include Extreme Learning Machine [22], Active learning [23], Mask-RCNN [24], Faster RCNN [25], Patch-wise based CNN model [26], RetinaNet [27], Graphical Neural Network [28], Reciprocal learning [29], and YOLO Variants [30]. The details of all the models is discussed in Supplementary Table S2

Segmentation methods have progressed from thresholding and morphological operations to U-Net-based models, with recent advancements in attention-based and hierarchical techniques. The architectural evolution demonstrates a shift from traditional edge-detection and region-growing methods to encoder-decoder architectures, with attention mechanisms and transformer-based segmentation models showing increased adoption. Despite this progress, only 10% of segmentation studies use Asian datasets, primarily due to the labour-intensive nature of pixel-level annotation requiring expert radiologist involvement, limited computational resources in many Asian institutions, and challenges in standardizing annotation protocols across diverse healthcare settings. Employed methods include Region Growing [31], ResU-SegNet [32], Connected SegNets [33], Otsu [28], Attention-based Active Learning [34], Hierarchical Gaussian Mixture Model with Expectation Maximization (HGMMEM) [35], Frangi Filter for vessel segmentation [36], and Coarse-to-Fine transformers [37] as discussed in Supplementary Table S3. Strategic approaches to overcome these challenges include semi-supervised learning methods to reduce annotation burden, active learning strategies for efficient sample selection, and cross-domain transfer learning techniques. Breast density segmentation remains largely unexplored due to the scarcity of annotated datasets, with no published work on Asian datasets, highlighting a critical research gap. The other density based segmentation techniques are discussed in Supplementary Table S4.

Lesion classification, a key focus area, categorizes findings into BI-RADS classes using DL-based feature extraction and end-to-end models. Architectural trends reveal a progression from traditional CNN-based feature extractors (VGG, ResNet) to more sophisticated approaches including attention mechanisms, ensemble methods, and transformer-based architectures, with increasing focus on interpretability and clinical applicability. While widely studied, only 13% of classification studies involve Asian datasets. All the Techniques used in lesion based feature extraction, classification are discussed in Supplementary Tables from S5 to S8. The focus map (Fig. 8a) also highlights this disparity. Various techniques used on Asian datasets include DenseNet [38], Extreme Learning Machine [22], CNN models [31, 39, 40], hierarchical fuzzy classifiers [32], EfficientNet [41], Weight-sharing MobileNet [42], ConvNet with SVM [43], Deep Adversarial Domain Adaptation [44], Multi-Scale CNN [45], ResNet [24], TL-optimized ResNet [46], squeeze-and-excitation-based Deep Neural Networks [47], Faster R-CNN [48], Convolutional Autoencoders [49], VGGNet [26], ResNet variants [36, 50, 51], Deep ELM with autoencoders [35, 52], RetinaNet [27], LSTM with Vanilla Siamese Network [53], Graph Convolutional Networks [28, 54], Student-Teacher Inter NRL [29], ensembled pre-trained models [55], Generative Adversarial Networks [56], Ensemble Self-Attention Transformer [57], and YOLO with Adaptive Multiscale Decision Fusion (AMDF) [58]. These studies indicate that while lesion classification has been extensively explored, the representation of Asian datasets remains limited, highlighting a gap for further research.

Mammographic breast density is a vital biomarker for treatment planning and BC prediction. Correctly identifying dense tissues in mammograms is key for efficient classification despite challenges like low contrast and background variations. The architectural landscape for density classification shows dominance of customized CNN architectures, with emerging trends toward multi-view learning, attentionguided approaches, and graph-based methods for capturing spatial relationships in mammographic data. DM plays a significant role in early BC detection and personalized therapy. From the Supplementary Tables from S9 to S12. it can be inferred that customized CNNs are the most common approach for breast density classification, with 22% of studies on Asian data using techniques such as Graph CNN [54], MobileNet [51], Multi-View Attention guided Residual Learning [59], and DenseNet [41] compared to detection and segmentation suggests greater accessibility of density annotations, though standardization across different density assessment protocols remains a challenge.

### RQ2: What are the unique challenges and considerations in applying DL techniques for BC detection using mammography on the Asian population?

BC poses a significant threat to Asian nations, with incidence and mortality rates much higher in developing countries compared to developed ones. When compared to patients in developed Asian and Western countries, a greater percentage of patients with BC are younger in developing Asian nations. Asia has a large population, and up to 25% of BC patients are young, indicating a significant number of cases [60]. Younger patients with BC typically have a poor prognosis due to increased density. One possible explanation for the negative correlation between early age and a poor prognosis for BC is that young people’s breast tissue is denser. Increased density can make identification difficult because it can mask abnormalities on mammograms, delaying diagnosis and treatment from the beginning. Dense breast tissue can obscure abnormalities on mammograms, delaying diagnosis and treatment.

Although DL models have shown promise in diagnosing BC in Caucasian populations, these models may be less effective on mammograms from women with denser breast tissues, such as those in Asian populations. Limited availability of Asian datasets hampers the performance of DL models, leading to biased predictions and challenges in training accurate models. To improve model performance, greater collaboration and sharing of diverse datasets from Asian populations are necessary [41, 51, 54]. In addition, the lack of publicly available Asian datasets hampers the effectiveness of DL models, as limited training data makes it difficult to optimize these models. Without sufficient data diversity, DL models may fail to account for health conditions and visual patterns unique to Asian populations, leading to biased predictions. This makes it challenging to develop universally effective models. Greater collaboration in collecting and sharing diverse datasets from Asian populations is essential to improve model performance and ensure more accurate insights.

### RQ3: What are the mammogram datasets used in the literature and why do Asian data need to be analyzed?

**Fig. 10:**
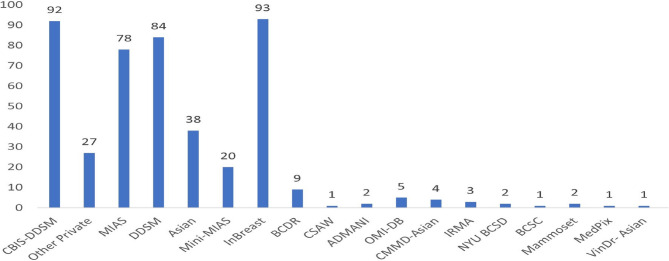
Frequently used Mammogram datasets

**Fig. 11:**
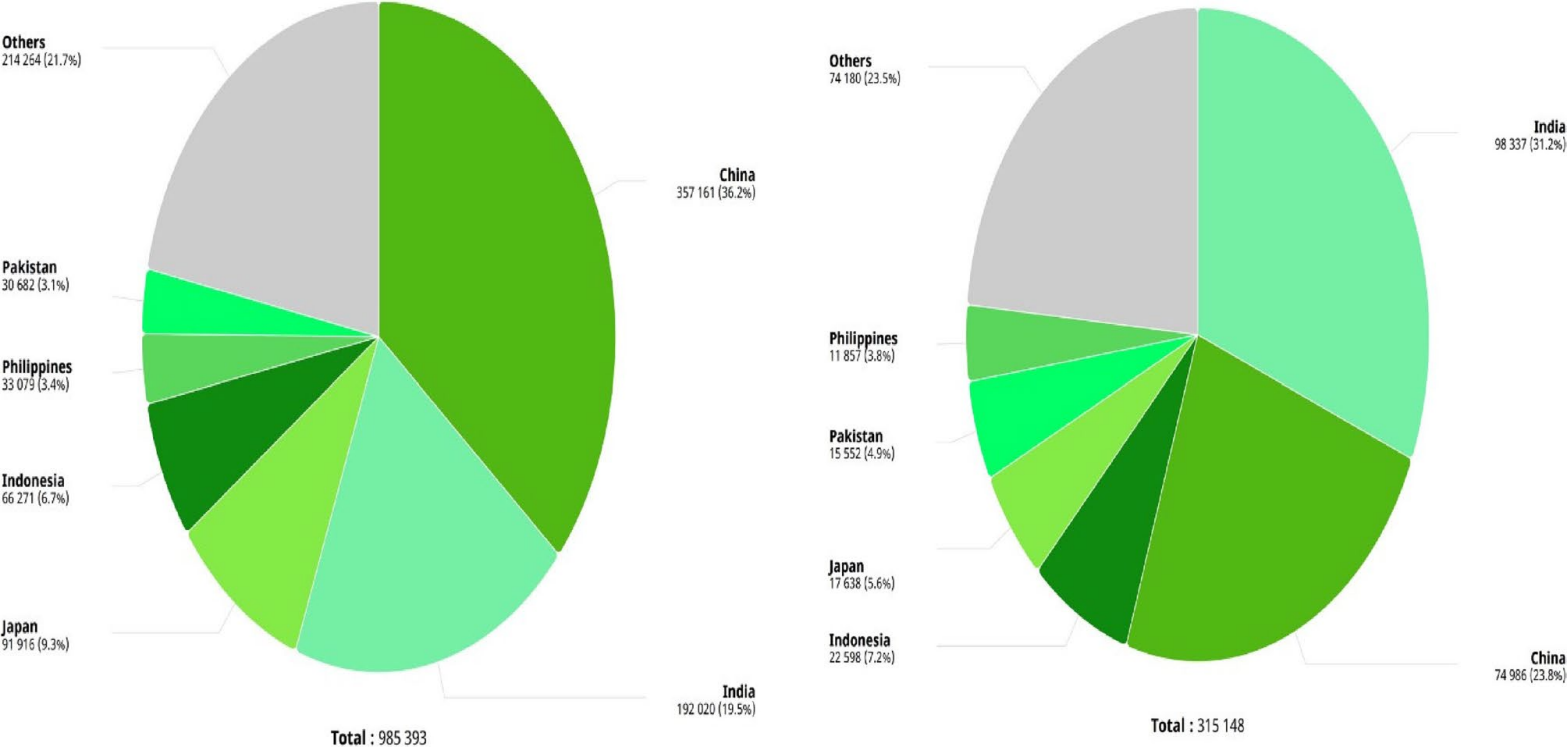
As per Globocan 2022, plot showing Incidence (left) and Mortality (right) rates of the Asian population due to Female BC in 2022 [2]

Figure 10 highlights that InBreast, CBIS-DDSM, and DDSM are the most commonly used datasets in studies, with Caucasian datasets being the most frequent, as shown in Table 3. Despite the leading incidence of BC in China and India, the highest mortality rate is observed in India, possibly due to a lack of awareness, as shown in Fig. 11. CMMD, an Asian cohort dataset, is available for DL model development but lacks annotations for mammogram regions of interest and information on breast density or BI-RADS categories, limiting its utility for lesion detection research. VinDrmammo, a Vietnamese dataset, offers information on breast density and BI-RADS assessment, but lacks molecular, histological, and pathology confirmation data, relying on radiologist expertise [15].

**Table 3.**
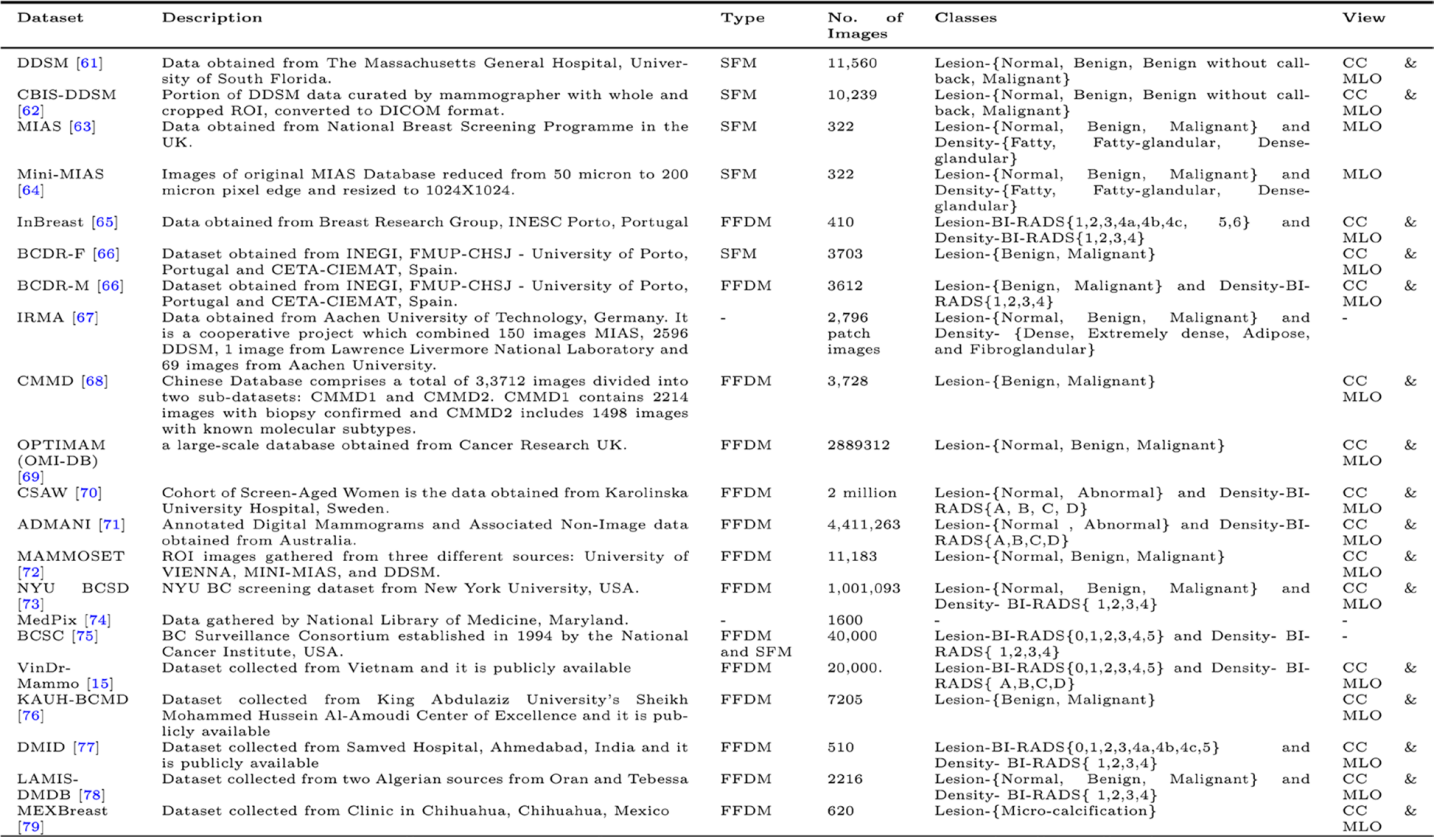
Publicly available breast cancer datasets [61–79]

There is limited work on Asian datasets, due to restricted accessibility and BC screening programs. Challenges such as lack of funding, inadequate infrastructure, and healthcare disparities hinder the establishment of screening programs. Cultural beliefs and financial circumstances also influence BC screening adoption and participation rates in some Asian countries. Raising awareness, encouraging routine screening, and investing in healthcare infrastructure are essential to increasing dataset availability. Partnerships between governments, medical institutions, and international entities can help establish successful screening programs and create large-scale datasets for research.

### RQ4: What is the potential impact of the pre-processing and augmentation techniques play on the robustness of the DL models?

The robustness of DL models can be significantly impacted by pre-processing and augmentation techniques. Pre-processing improves the input data before feeding it into DL models, including techniques like contrast enhancement, normalization, resizing, and noise reduction. However, FFDM images, due to their high resolution, typically require minimal pre-processing, mainly resizing and normalization. Supplementary Tables S13 to S15 provide an overview of noise reduction, image enhancement, artifact removal, and region-of-interest selection techniques. The adaptive median filter is commonly used for noise reduction, while contrast-limited adaptive histogram equalization (CLAHE) is frequently applied for image enhancement. Thresholding and morphological operations are used for artifact removal and region-of-interest selection.

**Fig. 12:**
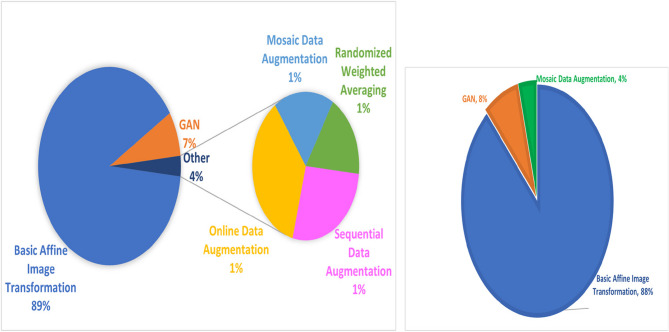
Augmentation Techniques used in the Literature for global datasets (left) and Asian datasets (right)

Data augmentation in medical imaging employs both traditional geometric transformations (rotation, flipping, scaling) and advanced synthetic data generation techniques, particularly GANs, to address data scarcity and improve model robustness. The pie chart in Fig. 12 highlights that while online data augmentation and basic transformations are common, GANs are increasingly used in recent studies [80–82]. While GANs have shown promise in generating realistic medical images, especially for dense breast tissue and rare pathological conditions, their clinical application faces significant challenges including anatomical implausibility, bias amplification, and potential generation of non-existent pathologies. Recent advances in diffusion models offer improved stability and mode coverage compared to traditional GANs [83], while physics-informed architectures incorporate domain knowledge to enhance anatomical realism [84]. However, rigorous clinical validation remains critical, requiring multistage assessment involving quantitative metrics Frechet Inception Distance (FID) and Structural Similarity Index Measure (SSIM), expert radiologist evaluation, and downstream task performance validation to ensure synthetic data quality and clinical applicability. To address the dominance of Caucasian datasets and improve diversity, we propose actionable solutions including crowdsourced annotation platforms for underrepresented populations, semi-supervised learning approaches to leverage unlabeled diverse datasets, and federated learning frameworks that enable collaborative model training across geographically distributed medical centers while preserving patient privacy.

### RQ5: What are the key drawbacks and future directions that should be explored to enhance precise and faster assessment, further improving diagnostic accuracy?

Several factors need to be addressed to enhance the accuracy and speed of BC assessments and improve diagnostic precision in this area. The key drawbacks and potential future directions can be organized into three thematic areas:

#### Data limitations and dataset enhancement

- Limited Publicly Available Asian Datasets: A significant challenge is the scarcity of diverse, comprehensive datasets, particularly from Asian populations with dense breast tissues. Currently, only two publicly available datasets from Vietnamese [15] and Chinese [68] female patients are accessible. As DL models require large training datasets, the limited availability restricts optimal results. Future research should focus on gathering larger, well-annotated datasets from various Asian populations to ensure diverse representation in BC data.
- Imbalanced Datasets: Class imbalance in training datasets (e.g., more benign cases than malignant ones) significantly affects model performance, causing models to be skewed towards the healthy class and slow convergence. Addressing this requires methods such as class weighting, over and under sampling, and synthetic lesion creation to increase malignant sample proportions [85].
- Bridging the Gap for Asian Populations: Many studies focus on datasets primarily consisting of Caucasian individuals, which may limit the applicability of models to Asian populations. Research should prioritize addressing the unique characteristics of Asian BC data, such as different demographic profiles and higher breast density.
- Cross-Population Model Validation: Many studies focus on single population datasets, limiting global applicability. Domain adaptation techniques should be implemented to transfer knowledge across populations with different demographic profiles and breast density characteristics. This includes progressive fine-tuning strategies and cross-cultural validation frameworks.

#### Model architecture and technical improvements

- Lack of Pre-Trained Models on Medical Images: Many DL models are pretrained on non-medical image datasets and lack necessary morphological awareness for medical applications. Future models should incorporate data from a wider range of medical modalities to improve morphological awareness, making them more adaptable to other medical tasks and enhancing accuracy across diverse clinical applications [86]. Technical solutions include: (1) Developing foundation models pretrained on large-scale medical imaging datasets [87], and (2) Self-supervised learning approaches using mammography-specific pretext tasks [88].
- Advanced Data Augmentation: While data augmentation is key for increasing training data diversity, excessive basic augmentation (rotation, flipping) may lead to overfitting. More advanced techniques should be explored, including generative models (GANs) [40, 80, 89], domain adaptation [44], Physics-informed augmentation that maintains anatomical consistency [84], and active learning [23, 34] to create realistic variations representing real-world scenarios.
- Multi-View Integration: Mammograms are typically taken in two views (CC and MLO), yet many studies treat these views separately, ignoring their intrinsic relationship. The MLO view is superior to the CC view for visualizing the lateral aspect of the breast, especially in the upper-outer quadrant, where pathological changes are more common. Although both views are typically interpreted, radiologists often assign a single BI-RADS density category, as CC and MLO views do not significantly differ in breast density assessment. Technical solutions for CC/MLO integration include: (1) Dual-stream CNNs with view-specific feature extraction followed by attention-based fusion, (2) Cross-view attention mechanisms that identify complementary information between views, (3) Late fusion strategies using ensemble methods with view-specific confidence weighting, and (4) Siamese networks for learning view-invariant representations. Recent advances demonstrate that such architectures significantly outperform single-view approaches [34, 53, 59, 73, 90–92].

#### Clinical translation and diagnostic enhancement

- Comprehensive BI-RADS Categorization: Classifying mammograms using only binary (Benign/Malignant) or three-class (Normal, Benign and Malignant) systems may not capture the full complexity involved. Physicians frequently struggle to discern subtle distinctions, such as B2, B3, or the narrow margins separating the B3, B4, and B4 subclasses from the B5 classification. There has not been much research on this topic, which suggests that a more thorough and sophisticated method of classifying mammograms is required to capture the subtle aspects of breast imaging fully. Future research should focus on developing hierarchical classification networks that model BI-RADS subcategory relationships to better capture the ordinal nature and clinical progression of these categories, and multi-task learning frameworks that jointly predict density and lesion characteristics to provide comprehensive assessment aligned with radiological practice.
- Digital Mammography Optimization: While screen-film mammography (SFM) has been replaced by full-field digital mammography (FFDM) in many screening programs, SFM still has some advantages, such as higher contrast resolution for fatty breasts [93]. However, FFDM offers superior results, particularly for younger women with dense breasts [94]. Future studies should explore the benefits of FFDM and the integration of digital mammograms in research.
- Future Risk Assessment: Despite significant advances in mammography-based BC assessments, future risk evaluation remains largely unexplored [53]. Developing reliable models for future risk prediction is crucial for personalized BC care and early intervention, enabling more accurate risk assessments and better clinical outcomes. Future research directions should include longitudinal deep learning models analyzing temporal changes in breast tissue to capture evolving risk patterns, multiinstance learning approaches for identifying subtle risk indicators that may not be apparent in single examinations, survival analysis networks for time-to-event prediction to estimate personalized risk timelines, and federated learning approaches for privacy-preserving risk model development across institutions to leverage diverse patient populations while maintaining data confidentiality.

By addressing these thematic areas systematically, the development of more precise and faster assessment methods in BC diagnosis can be achieved, enhancing diagnostic accuracy and patient outcomes.

### Recent developments (2024–2025)

Recent developments identified through supplementary hand search reveal several emerging trends that validate and extend the findings of this systematic review. These developments demonstrate the continued evolution of deep learning techniques for mammography analysis and confirm the relevance of research directions identified in this systematic analysis.

#### Advancements in detection techniques

Recent advancements in breast imaging have introduced sophisticated deep learning architectures that significantly enhance detection accuracy across multiple tasks. For microcalcification detection, novel approaches include YOLO-v8 with its anchorfree mechanism and decoupled head architecture [95], GravityNet utilizing gravity points as pixel-based anchors for small lesion detection [96], and Pro UNeXt algorithm incorporating micro-calcification learning blocks with fused-MBConv modules [97]. Hybrid Vision Transformers (ViT++) combined with CNNs leverage both contextual and visual features [98], while Deep Dilated Fully Convolutional Neural Networks (DDFCNN) provide one-step solutions for multi-scale anomaly detection [99]. For mass detection, cutting-edge models include RCM-YOLO with Residual Asymmetric Dilated convolution modules [100], Four-View Correlation and Contrastive Joint Learning Networks (FV-Net) for bilateral mammogram analysis [101], and Self-supervised Adversarial Adaptation Networks (SelfAdaptNet) combining SSL techniques with adversarial training to address domain-shift challenges [88].

Multi-view analysis has emerged as a dominant paradigm, with frameworks like BTMuda (Bi-level Multi-source Unsupervised Domain Adaptation) addressing both intra-domain and inter-domain variations through Three-Branch Mixed extractors combining CNNs and Transformers [102]. For breast density assessment, MV-DEFEAT [103] employs Dempster Shafer evidential theory to combine multi-view evidence with calibrated uncertainty, while Local Cross-View Transformers and Global Representation Collaborating (LCVT-GR) models [104] utilize parallel global-local analysis methods. Progressive Transfer Ensemble Learning [105] approaches stack multiple CNN architectures (VGG16, ResNet, EfficientNet, DenseNet, Xception) across multistep diagnostic processes, achieving refined classification results. These state-of-the-art techniques represent a paradigm shift toward automated feature learning, robust multi-view integration, and optimized deployment efficiency, moving beyond traditional handcrafted feature-based methods to address the complex challenges of breast tissue analysis.

#### Advancements in segmentation techniques

Recent developments in breast imaging segmentation have introduced sophisticated deep learning architectures that significantly advance precision in anatomical structure delineation. For mass segmentation, novel approaches include the Att-U-Node [101] architecture for automated breast tumor segmentation, SRMADNet [106] employing Swin ResUnet3 + for comprehensive mammogram segmentation, and hybrid YOLOv5MedSAM frameworks [107] combining object detection with specialized medical segmentation capabilities. Advanced variants like Deep Dilated Fully Convolutional Neural Networks (DDFCNN) [99] incorporate multi-scale feature and dilation modules for simultaneous detection of multiple abnormalities, while Multilevel Semantic Segmentation with GoogleNet and Multi Dilated Convolutions (GN-MDC) [108] integrates encoder-decoder architectures with multi-scale dilated convolutions for enhanced tumor segmentation and pectoral muscle removal. For microcalcification segmentation, the Pro UNeXt [97] algorithm specifically enhances UNeXt architecture with microcalcification detection blocks, fused-MBConv modules, and multiple-lossfunction training combining focal loss, Dice loss, and Hausdorff distance loss for optimal fine-tuning.

Breast density assessment has been revolutionized through sophisticated U-Netbased architectures and their variants, enabling multi-class semantic segmentation of critical anatomical structures including nipple, pectoral muscle, fibroglandular tissue, and fatty tissue. Notable innovations include PEMNet [109] incorporating attention mechanisms for improved pectoral muscle segmentation accuracy, U-Net-GAN hybrid two-stage methods for pectoral muscle shape estimation and refinement, and Automatic SegmenAN [110] employing adversarial neural networks with multi-scale L1 loss functions to capture both local and global spatial features. Advanced optimization techniques such as Bayesian optimization using Tree Parzen Estimator (TPE) [109] algorithms enhance model configuration, while Reinforcement Learning-based Semantic Segmentation (RLSS) [111] algorithms perform intelligent segmentation with and without pectoral muscle consideration to minimize information loss. These state-ofthe-art segmentation techniques represent a paradigm shift toward automated, precise anatomical structure delineation, enabling more accurate breast density assessment and lesion characterization compared to traditional manual segmentation approaches.

#### Advancements in classification techniques

Recent advances in breast imaging classification have introduced sophisticated multiview and cross-view learning architectures that significantly enhance diagnostic accuracy through comprehensive feature integration. Novel approaches include MVDEFEAT [103] employing Dempster-Shafer evidential theory and subjective logic to combine multi-view evidence for BI-RADS classification and malignancy assessment, achieving substantial improvements over traditional multi-view deep learning models. The Four-View Correlation and Contrastive Joint Learning Network (FV-Net) [101] leverages Cross-Mammogram Dual-Pathway Attention Modules for feature matching across all four standard views while incorporating Bilateral-Mammogram Contrastive Joint Learning for associative contrastive learning on positive and negative sample pairs. The LCVT-GR [104] model employs Local Cross-View Transformers for dependency learning between different views, while BRAIxDet [112] addresses incomplete annotations through two-stage semi-supervised learning combining multi-view mammogram classifiers with student-teacher frameworks using pseudo-labeling strategies. Complementing these multi-view approaches [10, 113], multi-modal classification systems integrate mammogram features with patient clinical data to enable comprehensive tumor classification that mirrors real-world clinical decision-making through feature fusion techniques combining CNN-extracted imaging features with traditional machine learning algorithms.

Semi-supervised and contrastive learning paradigms have revolutionized classification robustness and generalization capabilities in mammography analysis. The Attention-based Hybrid View Learning (AHVL) [114] framework incorporates Contrastive Switch Attention modules that integrate pre-trained CLIP language models for category embeddings as anchor points, enhancing feature discriminability especially under missing view conditions. Domain-Invariant Features Learning Framework (DIFLF) [115] employs contrastive learning through Style-Augmentation Modules and Content-Style Disentanglement Modules to extract domain-invariant content features while alleviating domain shifts for single-source domain generalization. Multi-group Similarity-Decoding-based Method (MG-SDM) [116] transforms model ensembling into feature-level fusion processes, calculating unique weights for individual features through similarity-decoding mechanisms. Progressive Transfer Ensemble Learning [105] combines multiple CNN architectures (VGG16, ResNet, EfficientNet, DenseNet, Xception) across multi-step diagnostic processes for refined classification into distinct breast cancer categories. These state-of-the-art classification techniques represent a paradigm shift toward sophisticated multi-view integration, self-supervised learning, and robust feature representation, enabling more accurate and generalizable breast cancer diagnosis compared to traditional single-view approaches.

#### Current landscape and research opportunities in breast cancer research using mammography

The current landscape in breast cancer detection has evolved toward sophisticated deep learning architectures with multi-view, multi-scale feature extraction, federated learning, reinforcement learning and hybrid CNN-Transformer approaches. Detection techniques now emphasize automated feature learning and robust multi-view integration, moving beyond traditional handcrafted feature-based methods to address complex breast tissue analysis challenges. Emerging technologies focus on multi-view and cross-view learning paradigms that integrate information from bilateral mammographic views for comprehensive tissue assessment. Semi-supervised and contrastive learning approaches are gaining traction, particularly for handling incomplete annotations and domain adaptation challenges. Advanced segmentation frameworks now enable simultaneous detection of multiple anatomical structures, while classification systems increasingly incorporate multi-modal approaches combining imaging features with clinical data.

During our hand search, we identified several new datasets that have been recently published, including KAUH-BCMD [76], DMID [77], LAMIS-DMDB [78], and MEXBreast [79], which have been updated in the Table 3. Notably, DMID and KAUHBCMD represent Asian datasets, contributing to regional diversity in breast cancer detection research. However, these datasets present certain limitations: KAUH-BCMD has only 2 lesion classes, which restricts multi-class research capabilities, while DMID, despite offering multiple BI-RADS categories for both lesion and density classification, has a relatively small dataset size that may limit model generalization.

Key research opportunities exist in several critical areas: optimizing sophisticated architectures for real-time clinical deployment and validating their robustness across diverse imaging centers and patient populations. Multi-view analysis requires better solutions for handling view misalignment and developing standardized evaluation metrics for cross-view performance assessment. Segmentation research needs unified frameworks capable of simultaneously handling multiple anatomical structures while maintaining computational efficiency for clinical workflows. Classification approaches present opportunities for developing more interpretable models that can explain decision-making processes to radiologists, standardizing multi-modal integration methods, and establishing comprehensive evaluation frameworks that account for real-world clinical variability and population diversity. The ultimate challenge remains seamless CAD system integration into clinical workflows, requiring interdisciplinary research combining AI innovation, clinical validation, regulatory approval processes, and healthcare provider acceptance strategies.

## Limitations

A few Limitations must be noted in this systematic Literature review. The review was limited to journals indexed in Scopus and Web of Science databases, potentially excluding relevant research available in other databases, conference proceedings, ArXiv preprints, and grey literature. While comprehensive, the search strategy may not have captured all relevant studies due to keyword specificity, though it was sufficient to address the stated research questions. The review was restricted to English-language publications, potentially excluding significant research published in other languages. Additionally, the review comprised papers published from 2018 to 2023, which might not reflect the most current advancements in this rapidly evolving field.

To address the temporal gap between the initial search and review completion, supplementary hand searching was conducted to identify prominent research published during this interval. While results from hand searching were not included in the formal data synthesis to maintain methodological rigor, this process helped ensure that research focus had not significantly shifted and that insights from the review remained current and relevant. This procedure also facilitated the discovery of related reviews and emerging research directions, broadening the scope and maintaining the review’s currency.

Despite rigorous procedures, potential data extraction and synthesis errors cannot be entirely eliminated. The review process may have been influenced by reviewer experience and potential biases, and results may not be broadly applicable to contexts or populations underrepresented in the reviewed studies. Future studies should address these limitations by incorporating multi-database searches, including non-English publications, and extending the temporal scope to provide more comprehensive coverage of the field.

## Conclusion

This systematic literature review, following the PRISMA approach, provides comprehensive insights into the evolving field of deep learning applications in mammography for breast cancer diagnosis, with particular emphasis on global and Asian perspectives. The review demonstrates significant progress in DL-based detection, segmentation, and classification of mammogram images, indicating a paradigm shift in BC diagnostic approaches. However, critical gaps remain that require immediate attention.

The analysis reveals a substantial knowledge void regarding model performance in heterogeneous populations, particularly Asian populations who exhibit distinct breast density characteristics and demographic profiles. Most existing studies utilize datasets from Caucasian populations, creating limited applicability to Asian contexts where higher breast density prevalence and different demographic factors directly impact diagnostic accuracy. This disparity is particularly concerning given that death rates from BC are noticeably higher in low- and middle-income nations, especially in Asia, highlighting the urgent need for early detection solutions tailored to these populations.

Furthermore, the review emphasized the lack of studies addressing the unique challenges globally, and also posed by higher breast density in Asian populations. Model development is further complicated by the scarcity of large-scale, reliable, and balanced datasets for BC diagnosis in this population. To close the current gaps in knowledge, further research should focus on developing stage-specific prediction, fusing data from multi-view images, and improving deep models to handle smaller datasets. Important topics for further research include the importance of domain-specific pre-trained models on medical images, resolving class imbalance problems, and the adaptability of digital images, enhancing the DL models for Asian populations, predicting future risk evaluation, and prospective clinical studies to evaluate these technologies to better understand their real-world efficacy.

In summary, this SLR has not only shed light on the future direction of DL in mammography for BC diagnosis but has also provided a thorough overview of the field’s status. This can set the stage for revolutionary developments in redefining BC diagnosis by addressing the specific challenges faced globally and within the Asian perspective, as well as embracing a broader and more diverse research goal.

## Supplementary Information

Supplementary Material 1.

## Data Availability

All data generated or analysed during this study are included in this published article and its supplementary information files.
