# Supplementary material for: A systematic literature review on mammography: deep learning techniques for breast cancer detection with global and Asian perspectives
Source: BMC Cancer. 2025 Oct 22;25:1627. doi: 10.1186/s12885-025-14876-5 (PMC12542462; doi:10.1186/s12885-025-14876-5)
Supplement: Supplementary file 1 — Supplementary Material 1. [file 12885_2025_14876_MOESM1_ESM.docx]

# Supplementary Material

1. **Supplementary Figures**

**
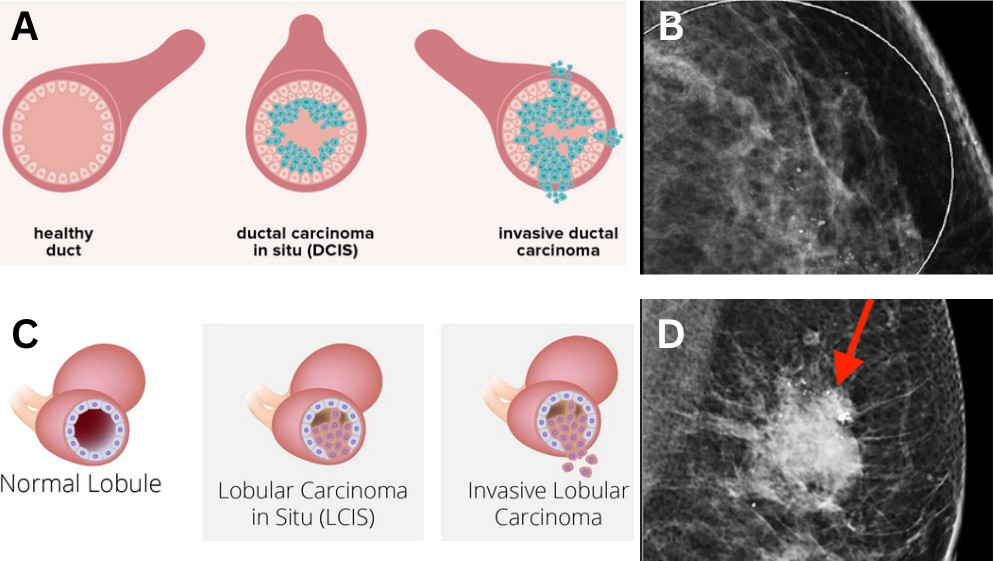
**

Figure S1: Supplementary Figure 1: Images A and B showing Normal, Abnormal, and Invasive duct [[246]](#_bookmark245) and Ductal carcinoma seen in mammogram [[135],](#_bookmark135) and Images C and D showing Normal, Abnormal, and Invasive Lobule [[217]](#_bookmark216) and Lobular carcinoma seen in mammogram [[77].](#_bookmark78)


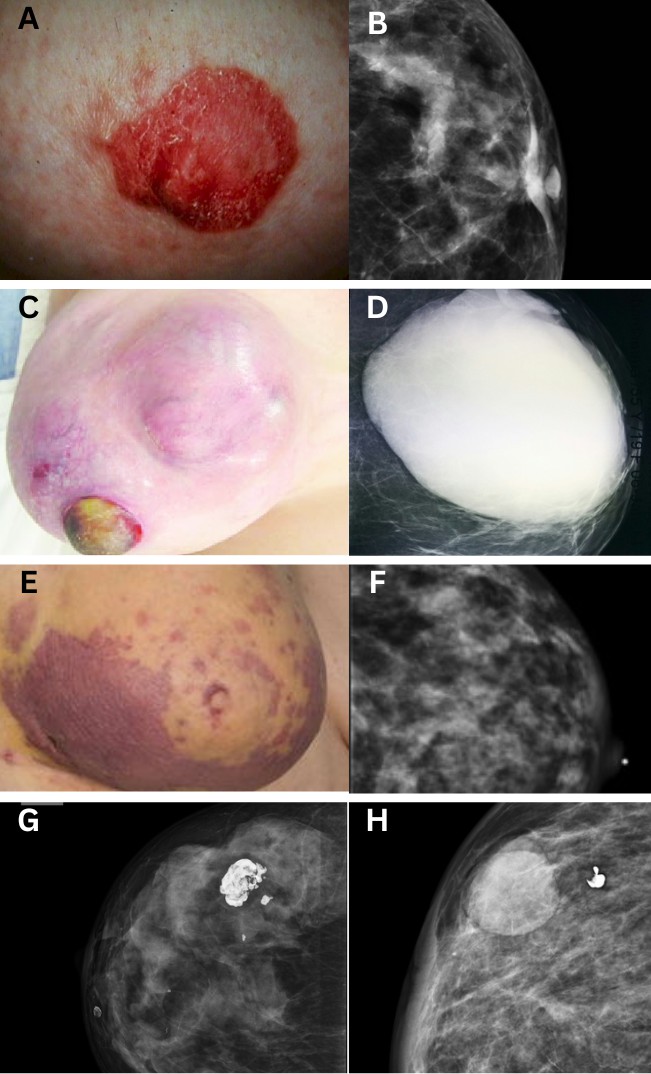


Figure S2: Supplementary Figure 2: Images A and B showing Paget Disease

[[50]](#_bookmark51) and as seen in mammogram [[136];](#_bookmark136) Images C and D showing Phyllodes Tumor [[31]](#_bookmark32) and as seen in mammogram [[131];](#_bookmark131) Images E and F showing Image of Angiosarcoma Tumor [[195]](#_bookmark194) and as seen in mammogram [[68]](#_bookmark69) ; Images G and H showing Fibroadenoma on the left [[74]](#_bookmark75) and Simple Cyst on the right [[134]](#_bookmark134) as seen in the mammogram


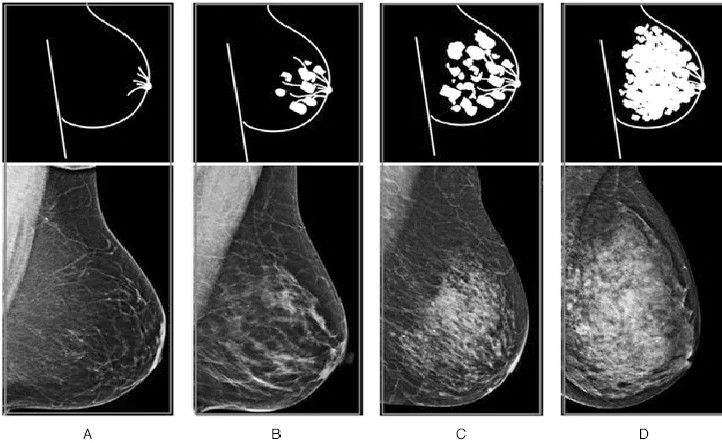


Figure S3: Supplementary Fig 3: Four categories Breast Density. A)Fatty, B) Scattered fibroglandular C)Heterogeneously dense D)Extremely Dense [[166]](#_bookmark166)


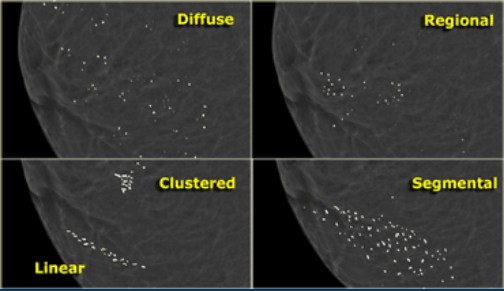


Figure S4: Supplementary Figure 4: Examples of distribution of Calcification

[[245]](#_bookmark244) A)Diffuse B) Regional C)Linear and Clustered D)Segmental


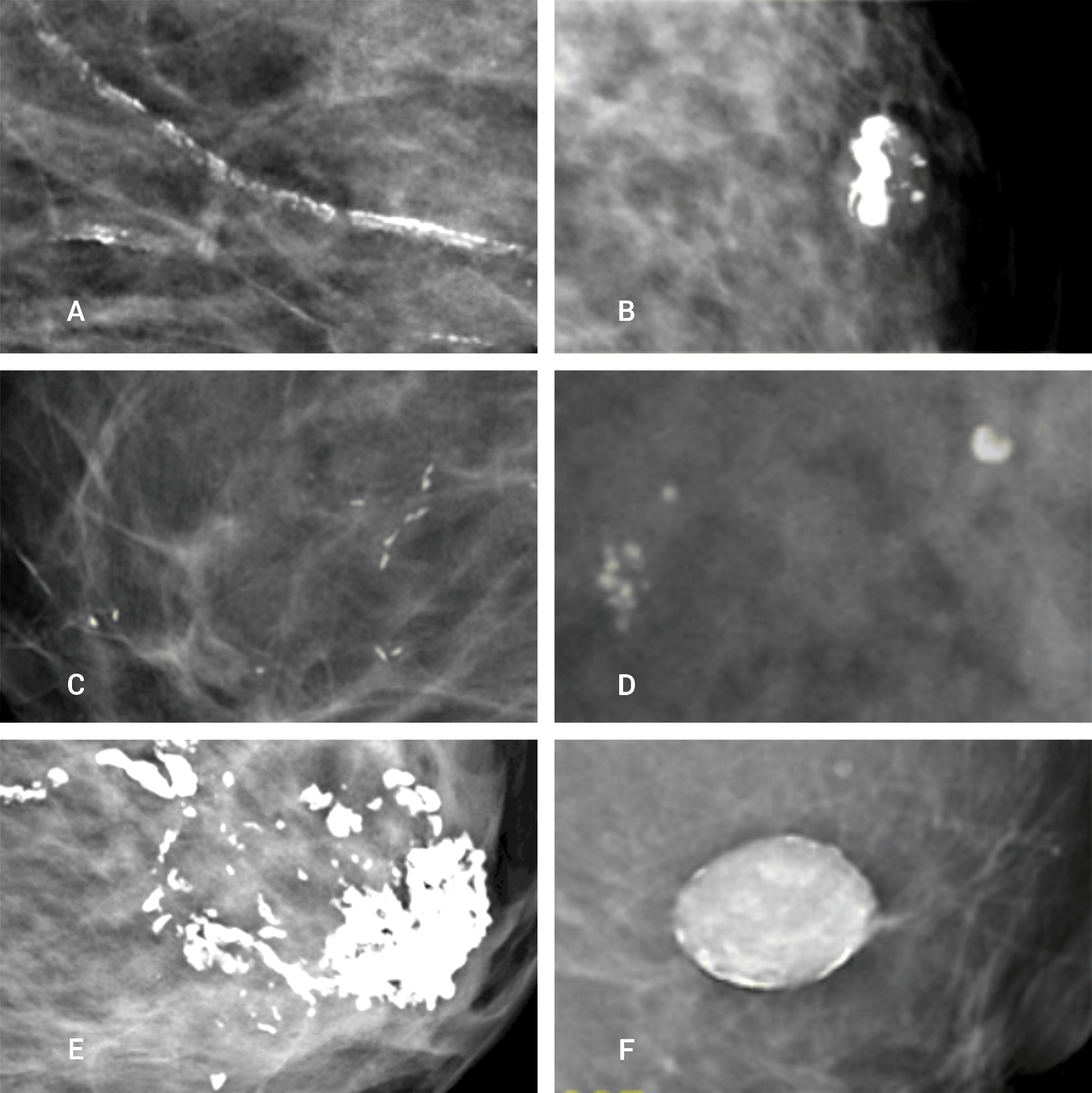


Figure S5: Supplementary Figure 5: Examples of Benign Calcification based on morphology [[245]](#_bookmark244) A)Vascular B) Popcorn-like C)Large rod-like D)Round and punctate E) Dystrophic F)Eggshell or Rim as seen in mammogram


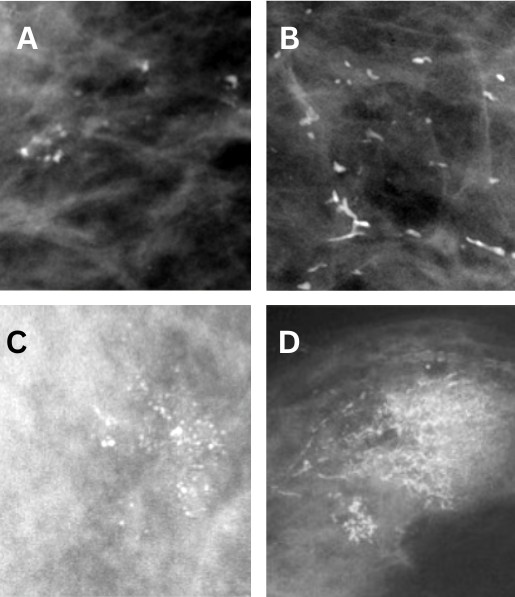


Figure S6: Supplementary Figure 6: Examples of Malignant Calcification based on morphology [[245]](#_bookmark244) A) Amorphous B) Coarse Hetrogenous C) Pleomorphic D) Fine linear branching as seen in mammogram [[245]](#_bookmark244)


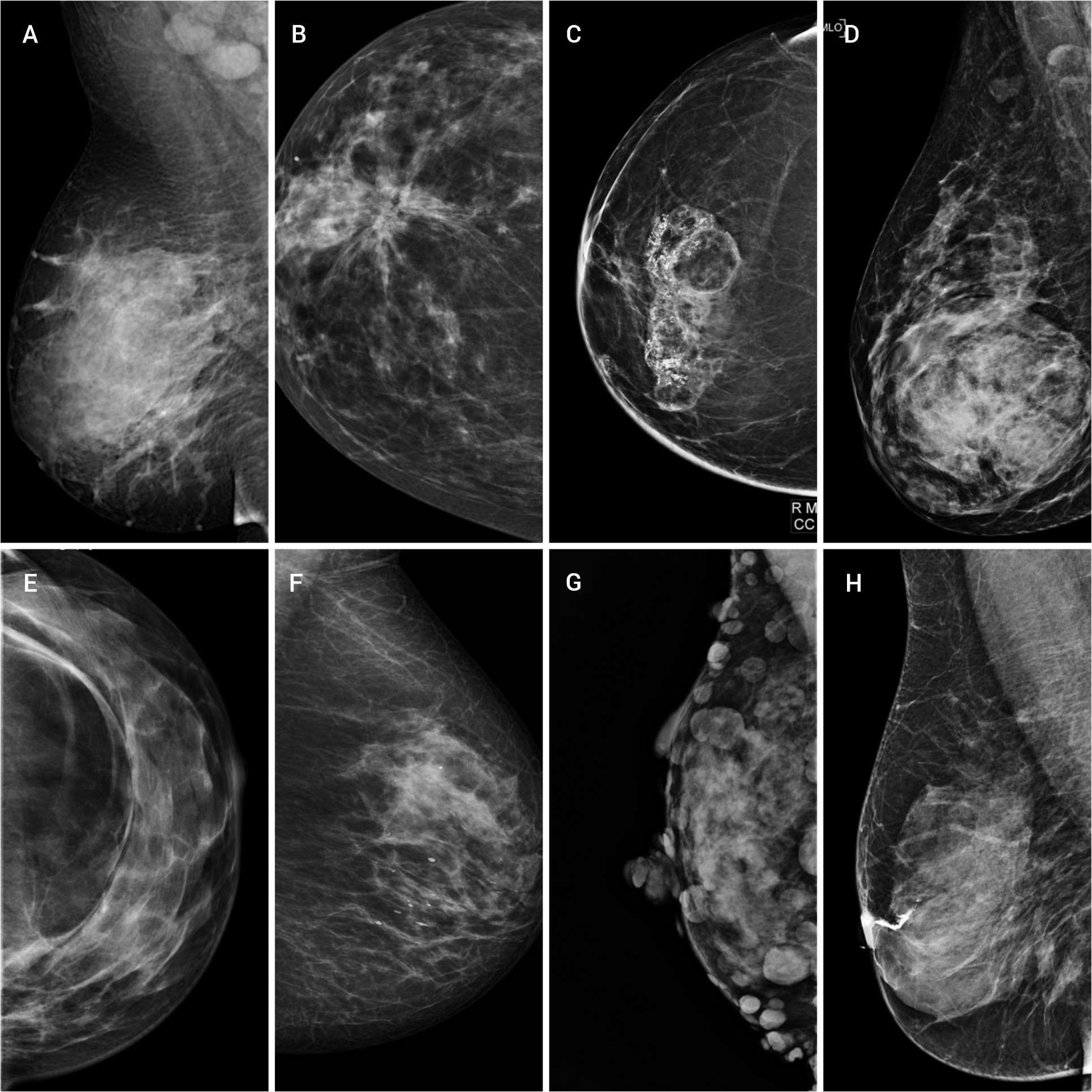


Figure S7: Supplementary Figure 7: Benign Breast mass A)Abscess [[125]](#_bookmark125) B) Sclerosing Adenosis [[293]](#_bookmark292) C)Fat Necrosis [[177]](#_bookmark176) D)Hamartoma [[211]](#_bookmark210) E)Lipoma

[[42]](#_bookmark43) F) Mastitis [[40]](#_bookmark41) G)Neurofibroma [[254]](#_bookmark253) H) Intraductal Papilloma [[178]](#_bookmark177)


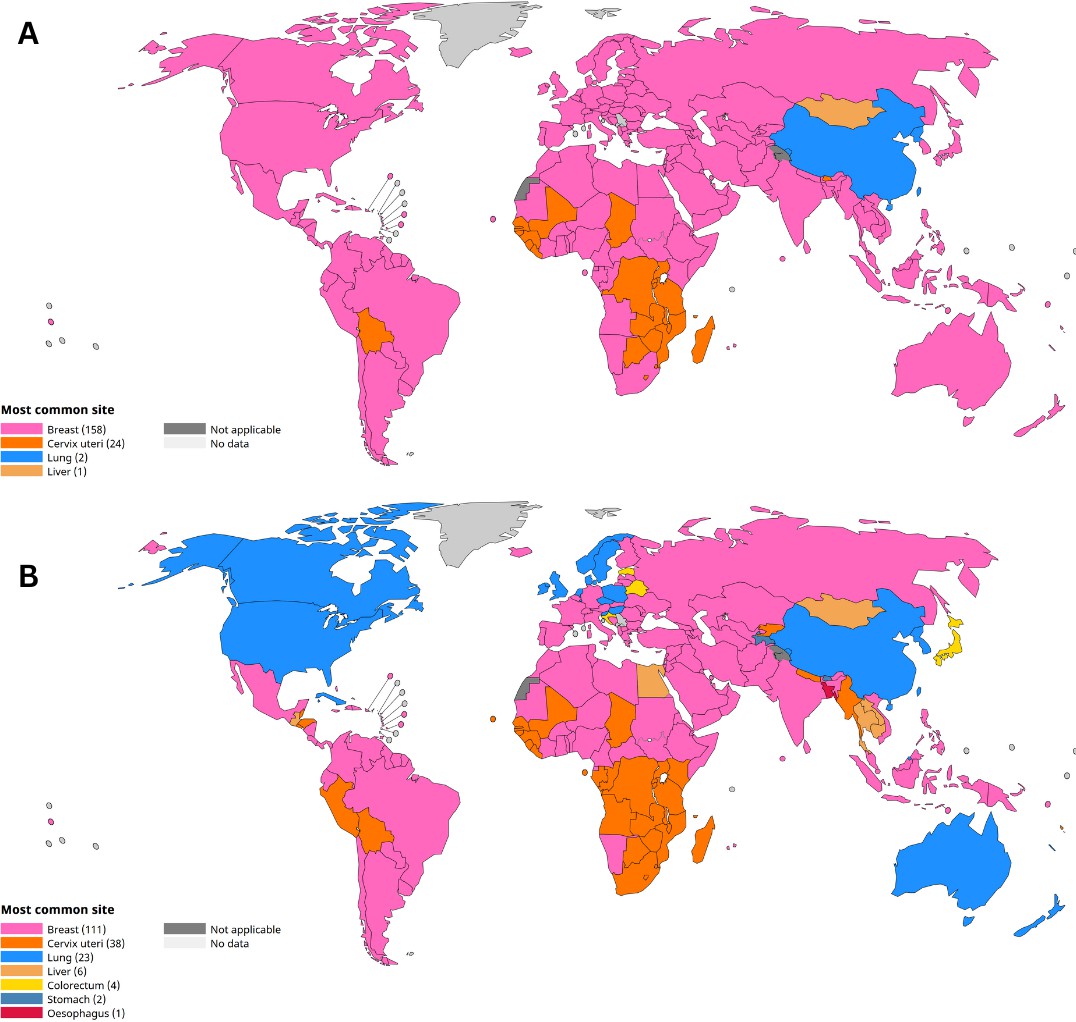


Figure S8: Supplementary Figure 8:Top cancer per country, females, all ages, estimated number of A) Incidence and B) Mortality in 2022, [310 ]


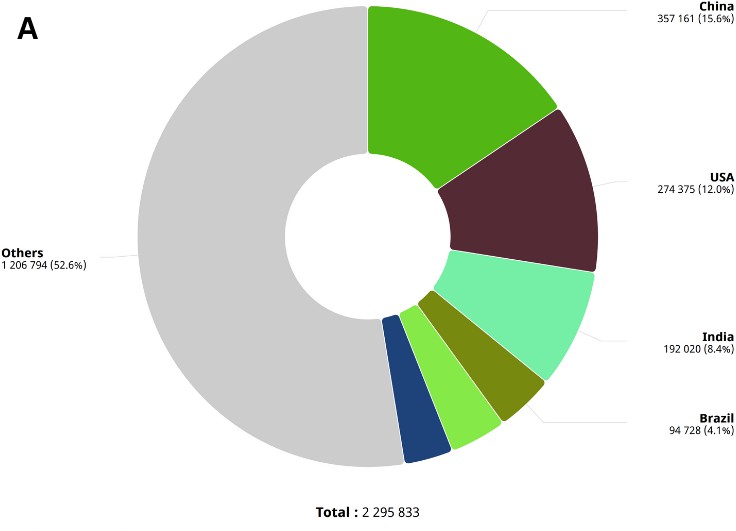

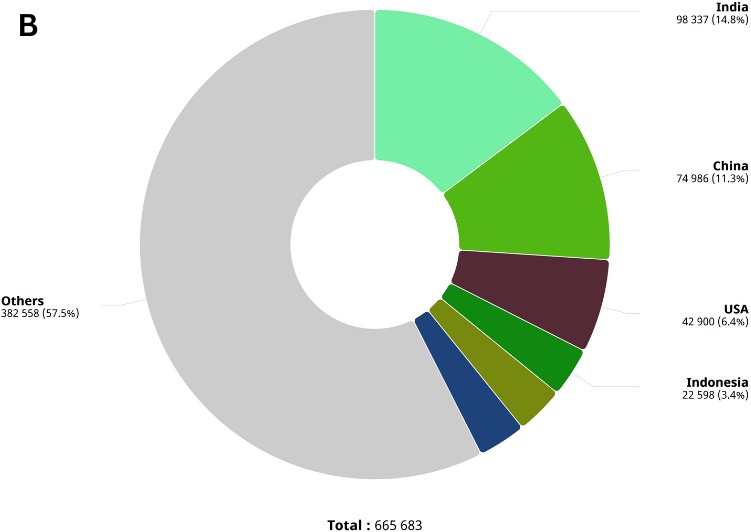


Figure S9: Supplementary Figure 9: Estimated A) Incidence B) Morality in 2022, females, all ages in top 10 ranked countries [310]


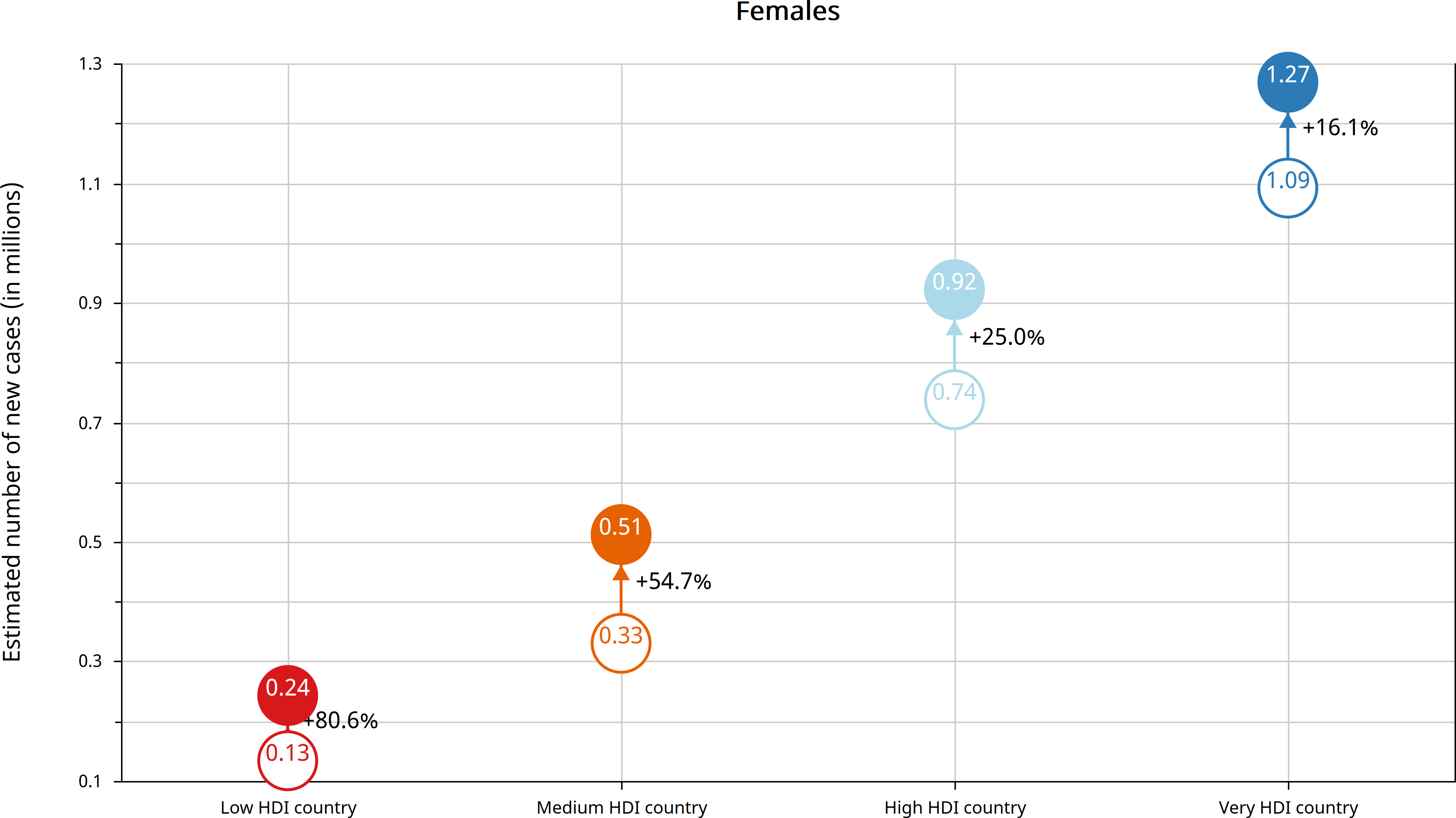


Figure S10: Supplementary Figure 10: Estimated new incident cases in various HDI countries from 2022 to 2040[310]

# Supplementary Tables

All the Asian dataset related studies are cited in red colour in all the Supple- mentary tables from S3 to S16.

Table S1: Supplementary Table S1: Details of search string and total papers extracted

| Database | Search String and Conditions | Search Date | Total Papers |
| --- | --- | --- | --- |
| Scopus | TITLE-ABS-KEY ("Breast Cancer") | 15 November | 584 |
|  | AND TITLE-ABS-KEY ("mammog*") | 2023 |  |
|  | AND TITLE-ABS-KEY ("Deep Learn- |  |  |
|  | ing") AND TITLE-ABS-KEY ("detec- |  |  |
|  | tion" OR "segmentation" OR "clas- |  |  |
|  | sification" OR "identification") AND |  |  |
|  | PUBYEAR *>* 2017 AND PUBYEAR |  |  |
|  | *<* 2024 AND (LIMIT-TO (SRCTYPE, |  |  |
|  | "j")) AND (LIMIT-TO (DOCTYPE, |  |  |
|  | "ar")) AND (LIMIT-TO (LANGUAGE, |  |  |
|  | "English")) |  |  |
| Web of Science | ((TS=("BC") AND TS=("mammog*") | 15 November | 467 |
|  | AND TS=("DL") AND | 2023 |  |
|  | TS=("detection" OR "segmentation" |  |  |
|  | OR "classification" OR "identifica- |  |  |
|  | tion")) AND PY=(2018-2023)) AND |  |  |
|  | LA=(English) and ’document type’ |  |  |
|  | limited to "Article" and "Early Access" |  |  |
|  | papers |  |  |
| Total |  |  | 1051 |

Table S2: Supplementary Table S2: Various DL-based breast lesions detection techniques

## Techniques References Description

YOLO Variants [[200]](#_bookmark199), [[45,](#_bookmark46) [213,](#_bookmark212) [299,](#_bookmark298) [307,](#_bookmark306)

[35,](#_bookmark36) [36,](#_bookmark37) [8,](#_bookmark9) [10,](#_bookmark11) [13,](#_bookmark14) [290,](#_bookmark289)

[291]](#_bookmark290)

Single shot detector for simultaneous detection and classification, using versions YOLOv2, YOLOv3, YOLOv4.

Threshold Methods [[258]](#_bookmark257)[,[9]](#_bookmark10) Includes traditional segmentation using morphology and template

matching based on intensity thresholds.

U-Net Variants [[247,](#_bookmark246) [138]](#_bookmark138) Common for segmentation, extended with SE blocks (Squeeze U-

Net) for enhanced feature learning.

Region Proposal Methods

[[261,](#_bookmark260) [148]](#_bookmark148) [[5,](#_bookmark6) [216,](#_bookmark215) [113,](#_bookmark113)

[139,](#_bookmark139) [285]](#_bookmark284)

Faster R-CNN and Mask R-CNN combine proposal networks with object detection and segmentation capabilities.

Multi-task Learning [[89]](#_bookmark90) Handles detection, segmentation, and classification tasks with customized architectures.

CNN Deep Features + Unsupervised ELM

[[284]](#_bookmark283) Extreme Learning Machine (ELM) is a single hidden layer feed- forward network. Unsupervised ELM training is appropriate for clustering tasks with limited labeled data, combined with CNN deep features for enhanced performance.

OLSTM [[244]](#_bookmark243) Optimized LSTM (OLSTM) reduces vanishing gradient issues

experienced in standard RNNs, enhancing model stability and

performance.

| Anatomy-aware Net- works | [[277]](#_bookmark276) | Combines teacher-student models to enhance segmentation with poor annotations. |
| --- | --- | --- |
| MSER detection + fea- | [[105]](#_bookmark105) | Maximally Stable External Region used to detect breast masses by |
| ture matching |  | matching features extracted from mammogram images |
| Active and Self-paced | [[237]](#_bookmark236) | This ranks unannotated samples using informativeness algo- |
| Learning |  | rithms, while SPL improves robustness by selecting informative |
|  |  | samples from unannotated data. |
| Transformer-based Mod- | [[91,](#_bookmark92) [43,](#_bookmark44) [119,](#_bookmark119) [255]](#_bookmark254) | DEtection TRansformer(DETR) and YOLO-LOGO use anchor- |
| els  Graph-based Ap- | [[292]](#_bookmark291) | free detection with transformers for robust performance.  Graph CNNs represent lesions as nodes, leveraging spatial embed- |
| proaches |  | dings and deep features. |
| Mixed Supervised Meth- | [[263]](#_bookmark262) | Combines self-supervised and weakly supervised learning for im- |
| ods |  | proved abnormality detection. |
| Attention-based Models | [[133]](#_bookmark133) [[17,](#_bookmark18) [276]](#_bookmark275) | Uses attention mechanisms to improve model focus on critical re- |
|  |  | gions in medical images. |
| RetinaNet | [[272]](#_bookmark271) | Uses a DL backbone with FPN and two subnetworks for classifi- |
| cation and bounding box regression. | | |

Table S3: Supplementary Table S3: Various DL-based breast lesion segmentation techniques

## Technique References Description

Multi-task Learning

[[89]](#_bookmark90) Customized multitask learning architecture designed to handle detection, segmentation and classification tasks

FrCN [[8,](#_bookmark9) [44,](#_bookmark45) [45]](#_bookmark46) Full resolution constitutional neural network is used for mass segmentation at the pixel level. FrCN consists of successive encoder and decoder networks. The input retains the essential information while maintaining spatial resolution.

Region Growing [[96]](#_bookmark96)[,[90,](#_bookmark91)

[163,](#_bookmark163) [70,](#_bookmark71)

[219,](#_bookmark218) [1,](#_bookmark2) [185,](#_bookmark184)

[186,](#_bookmark185) [203]](#_bookmark202)

Starting with seed points, it gradually expands regions by incorporating neighboring pix- els based on similarity criteria like intensity or texture. Advanced implementations use optimization techniques like firefly-updated chicken-based Chicken Swarm Optimization and adaptive fuzzy Cmeans for initial seed points. Post-growth, unwanted pixel values are removed using thresholding.

U-Net and Vari- ants

SegNet and Variants

[[62,](#_bookmark63)

[88]](#_bookmark89)[,[252,](#_bookmark251)

[206,](#_bookmark205) [270,](#_bookmark269)

[41,](#_bookmark42) [301,](#_bookmark300) [98,](#_bookmark98)

[94,](#_bookmark94) [26,](#_bookmark27) [309,](#_bookmark308)

[1,](#_bookmark2) [244,](#_bookmark243) [194,](#_bookmark193)

[61,](#_bookmark62) [268,](#_bookmark267)

[283,](#_bookmark282) [151,](#_bookmark151)

[257,](#_bookmark256) [291,](#_bookmark290)

[54,](#_bookmark55) [34,](#_bookmark35) [138]](#_bookmark138) []

[[19,](#_bookmark20) [33,](#_bookmark34) [238]](#_bookmark237)

[[1,](#_bookmark2) [212]](#_bookmark211)

Comprehensive family of architectures including:

- Basic U-Net: U-shaped structure with contracting/expansive paths and skip connections
- Modified/Optimized versions: Using Black Widow Optimization and ResNet-style connections
- U-Net++: Enhanced with nested skip connections
- Attention-based variants: Incorporating attention mechanisms and active learning
- Multi-task U-Net: Including segmentation-specific loss functions
- Connected U-Net: Using additional skip connections between multiple U-Nets
- Squeeze U-Net: Incorporating fire modules for depth manipulation Family of architectures including:
- Basic SegNet: Encoder-decoder with max-pooling indices
- Connected SegNet: Dual encoder-decoder with additional connections
- U-SegNet: Hybrid combining SegNet and U-Net approaches

K-means [[15]](#_bookmark16) K-means clustering divides pixels into K clusters based on similarity to cluster centroids, iteratively updating assignments until convergence.

Thresholding

[[154,](#_bookmark154) [292,](#_bookmark291)

Various thresholding approaches include:

| Based | [52,](#_bookmark53) [66,](#_bookmark67) [106,](#_bookmark106) |  |
| --- | --- | --- |
|  | [241,](#_bookmark240) [213,](#_bookmark212) | - Basic thresholding for pixel classification |
|  | [128,](#_bookmark128) [297]](#_bookmark296) | - Otsu’s method for optimal threshold calculation |
|  |  | - Factorized Otsu for handling imbalanced intensities |
|  |  | - Multilevel thresholding with Kapur’s based Shell Game Optimization (SGO) |
|  |  | - Combined with morphological operations and color space transformations |
| FCN and Vari- ants  DeepLab | [[1,](#_bookmark2) [288,](#_bookmark287) [29]](#_bookmark30)  [[208,](#_bookmark207) [308,](#_bookmark307) | It uses convolutional and deconvolutional layers for encoding/decoding without fully connected layers. It includes the Dilated-Net variant with atrous convolutions for larger receptive fields.  Family of semantic segmentation models using Atrous Convolution and Spatial Pyramid |
|  | [268,](#_bookmark267) [7]](#_bookmark8) | Pooling. Multiple versions (v1-v5) with various backbones and optimizations. |

*Continued on next page*

Table S[3](#_bookmark0) continued

## Technique References Description

Transformer- based

Modern Deep Learning Archi- tectures

[[62]](#_bookmark63),

[[155,](#_bookmark155) [255]](#_bookmark254)

[[248,](#_bookmark247) [7,](#_bookmark8) [159,](#_bookmark159)

[233,](#_bookmark232) [276,](#_bookmark275)

[207]](#_bookmark206)

Modern architectures including:

- Transformer encoder-decoder models
- C2FTrans with cross-scale transformers
- YOLO-LOGO combining transformers with YOLO detection Advanced architectural approaches including:
- Mask R-CNN: Extends Faster R-CNN for instance segmentation, combining object detection, bounding box regression, and pixel-level segmentation
- Dual Contextual Affinity Network (DCANet): Uses lightweight contextual affinity components with global-guided and local-guided modules for efficient encoding-decoding
- CNN from scratch: Custom segmentation networks with grasshopper optimization
- GoogleNet-based: Modified GoogLeNet architecture with specialized top layers for semantic segmentation

Transfer Learn- ing Based

Weakly Su-

pervised Ap- proaches

[[117]](#_bookmark117) Transfer learning approaches include:

- VGG-16 based: Uses pre-trained VGG-16 with updated gradient-based optimization hyperparameters
- Series network integration: Combines feature extraction capabilities with structured layer organization

[[25,](#_bookmark26) [263]](#_bookmark262) Advanced supervision techniques including:

- LatentCADx: Combined classification-segmentation architecture with weakly su- pervised loss function for high-precision boundary detection
- Mixed self-supervised: Two-phase approach using self-supervised reconstruction and weakly supervised detection with image-wise ground truth

| Advanced | Hy- | [[147,](#_bookmark147) [210,](#_bookmark209) | Sophisticated combinations include: |
| --- | --- | --- | --- |
| brid |  | [152,](#_bookmark152) [283,](#_bookmark282) |  |
| Anatomical |  | [234]](#_bookmark233)  [[274]](#_bookmark273), [[260]](#_bookmark259) | - Dual Core Net: Combining LPL and CGL learners - Multi-route CNN: Multiple feature extraction paths - Super-pixel Pooling cGAN: Using super-pixel structure - ESP-Net: Using point-wise convolutions with dilated spatial pyramids Approaches focusing on anatomical structures: |

- - Anatomy-aware weakly-supervised learning with Teacher-Student networks
  - Frangi Filter for blood vessel segmentation

| Traditional Pro- | [[144,](#_bookmark144) [28,](#_bookmark29) [16,](#_bookmark17) | Classical approaches enhanced with modern techniques: |
| --- | --- | --- |
| cessing | [128,](#_bookmark128) [297,](#_bookmark296) |  |
|  | [127,](#_bookmark127) [84]](#_bookmark85) | - Chan-Vese Level set with Sobel filter |
|  | | - Adaptively regularized kernel-based fuzzy c-means |
|  |  | - Gaussian Mixture Model filtering |
|  |  | - Multilevel thresholding with optimization |
|  |  | - Polynomial curve fitting |
|  |  | - Tsallis entropy: Measures image uncertainty/disorder for segmentation, derived |

from Shannon entropy and based on likelihood distribution of image state

Table S4: Supplementary Table S4: Various DL based breast density segmentation techniques

## Techniques References Description

Entirely CNN (ECNN)

Weighted Adap- tive multi-

task learn-

ing approach (MTLSegNet) Confusion Ma- trix Y-Net

[[190]](#_bookmark189) A Regression Architecture ECNN improves classical CNN networks by continuously rebuilding a probabilistic dense tissue mask, resulting in a loss function that maximizes the DICE score.

[[99]](#_bookmark99) MTLSegNet represents a deep-learning architecture used to segment mammograms and estimate breast density. It uses a weight-adaptive multitask learning approach that segments the breast area and dense tissues while estimating breast percentage density.

[[143]](#_bookmark143) CM-YNet is a fully automated DL-based method for estimating breast density. It is a hybrid threshold-based and mask-based method that uses noisy labels to determine the density-tissue mask and segmentation criteria.

U-Net [[267]](#_bookmark266) Explained in Table [4](#_bookmark0)

cGAN [[221]](#_bookmark220) The cGAN is made up of a generator network as well as a discriminator network,

learns adversarially, with the generator aiming to generate masks distinct from ground truth and the discriminator aiming to classify the generated mask accurately.

Otsu [[179,](#_bookmark178) [275]](#_bookmark274) Otsu’s technique employs the grayscale histogram of an image to identify the

optimum threshold level that distinguishes two regions with the highest inter-class variance.

Table S5: Supplementary Table S5: Various DL based breast lesion feature extraction techniques

## Category References Description

Modern CNN Architectures

**ResNet**: [[160,](#_bookmark160) [272]](#_bookmark271), [[48,](#_bookmark49) [81,](#_bookmark82) [132,](#_bookmark132)

[149,](#_bookmark149) [214,](#_bookmark213) [222,](#_bookmark221) [226,](#_bookmark225) [27,](#_bookmark28) [49,](#_bookmark50) [57,](#_bookmark58)

[106,](#_bookmark106) [208,](#_bookmark207) [230,](#_bookmark229) [281,](#_bookmark280) [280]](#_bookmark279)

**Inception Family**: [[160]](#_bookmark160), [[81,](#_bookmark82)

[101,](#_bookmark101) [29,](#_bookmark30) [175,](#_bookmark174) [208](#_bookmark207), [176,](#_bookmark175) [140,](#_bookmark140) [27,](#_bookmark28)

[49]](#_bookmark50)

**DenseNet**: [[160]](#_bookmark160), [[141,](#_bookmark141) [302,](#_bookmark301) [49,](#_bookmark50)

[57,](#_bookmark58) [219,](#_bookmark218) [164]](#_bookmark164)

Modern deep learning architectures:

- ResNet: Uses residual connections to handle vanishing gradients
- Inception variants: Employs parallel convolutions at multiple scales
- DenseNet: Features dense connections between layers

*Continued on next page*

Table S[5](#_bookmark1) continued

## Category References Description

Lightweight Ar- chitectures

**MobileNet**: [[160]](#_bookmark160), [[20,](#_bookmark21) [70,](#_bookmark71) [300,](#_bookmark299)

[49,](#_bookmark50) [208,](#_bookmark207) [285,](#_bookmark284) [164]](#_bookmark164)

**SqueezeNet**: [[41,](#_bookmark42) [219]](#_bookmark218)

**EfficientNet**: [[48,](#_bookmark49) [115,](#_bookmark115) [29,](#_bookmark30) [49,](#_bookmark50)

[57]](#_bookmark58)

**ShuffleNet**: [[208]](#_bookmark207)

Efficiency-optimized architectures:

- MobileNet: Uses depthwise separable convolutions
- SqueezeNet: Employs 1x1 convolutions
- EfficientNet: Optimizes network dimensions
- ShuffleNet: Uses channel shuffling

Classic CNNs **AlexNet**: [[304]](#_bookmark303), [[81,](#_bookmark82) [163,](#_bookmark163) [224,](#_bookmark223)

[228,](#_bookmark227) [227,](#_bookmark226) [241,](#_bookmark240) [6,](#_bookmark7) [27,](#_bookmark28) [106]](#_bookmark106)

**VGG**: [[160]](#_bookmark160), [[121,](#_bookmark121) [196,](#_bookmark195) [222,](#_bookmark221) [226,](#_bookmark225)

[228,](#_bookmark227) [227,](#_bookmark226) [295,](#_bookmark294) [27,](#_bookmark28) [49,](#_bookmark50) [57,](#_bookmark58) [175,](#_bookmark174)

[208]](#_bookmark207)

**GoogleNet/Inception V1**: [[190,](#_bookmark189) [222,](#_bookmark221) [228,](#_bookmark227) [227,](#_bookmark226) [27,](#_bookmark28) [208](#_bookmark207)]

Pioneering CNN architectures:

- AlexNet: First GPU-accelerated CNN, ImageNet 2012 winner
- VGG: made up of stacks of layers of convolution with small 3x3 filters, which provide a systematic approach to feature extraction.
- GoogleNet: Introduced inception modules

Advanced CNN Variants

Transform- Based Methods

Neural Network Variants

**Xception**: [[112]](#_bookmark112), [[73,](#_bookmark74) [208]](#_bookmark207)

**Inception-ResNet**: [[140,](#_bookmark140) [27,](#_bookmark28)

[49]](#_bookmark50)

**DarkNet**: [[13]](#_bookmark14)

**Highway-network CNN**: [[65]](#_bookmark66)

**Discrete Wavelet**: [[52,](#_bookmark53) [142,](#_bookmark142)

[214,](#_bookmark213) [240,](#_bookmark239) [180]](#_bookmark179)

**Gabor Wavelet**: [[116,](#_bookmark116) [171]](#_bookmark170)

**Fast Discrete Curvelet**: [[232]](#_bookmark231)

**PCNN**: [[22]](#_bookmark23)

**DWNN**: [[72]](#_bookmark73)

**CapsNet**: [[128,](#_bookmark128) [29]](#_bookmark30)

**NasNet**: [[300]](#_bookmark299)

**CAE**: [[183]](#_bookmark182)

Enhanced architectures:

- Xception: Uses depthwise separable convolutions
- Inception-ResNet: Combines inception modules with residual connections
- DarkNet: Optimized for real-time processing
- Highway-network: Dynamic feature gathering with bypass mechanisms

Multiple transform approaches:

- Wavelet transforms: Multi-scale frequency decomposition
- Gabor filters: Texture and pattern capture
- Curvelet transform: Complex geometric structure analysis Specialized neural architectures:
- PCNN: Uses synchronized pulses to emphasize regions with distinct patterns in images.
- DWNN: Obtains image approximations and details by capturing low-frequency and high-frequency components such as edges
- CapsNet: Hierarchical feature relationships, overcomes the drawbacks of conventional convolutional neural networks by capturing hierarchical associations among features.
- NasNet: Employs scalable convolutional cells derived from data.
- CAE: Unsupervised feature learning via autoencoding

*Continued on next page*

Table S[5](#_bookmark1) continued

## Category References Description

Gradient-Based Methods

**HOG**: [[222,](#_bookmark221) [249]](#_bookmark248)

**PHOG**: [[163]](#_bookmark163)

**GFCF**: [[298]](#_bookmark297)

Gradient-based feature extraction:

- HOG: Gradient orientation histograms,depicts the distribution of gradient orientations, emphasizing edges and patterns.
- PHOG: Pyramid structure enhanced HOG, enhances HOG by adding a pyramid structure for better scale invariance and improved shape representation.
- GFCF: Gradient field convergence analysis, pixel A can be stated as the gradient field’s convergence within a circular region centered on A and having a radius of r and N A neighborhood pixels.

| Traditional Fea- | **Texture and Color**: [[33,](#_bookmark34) [161,](#_bookmark161) | Classical image analysis: |
| --- | --- | --- |
| ture Descriptors | [238,](#_bookmark237) [284,](#_bookmark283) [304]](#_bookmark303), [[15,](#_bookmark16) [52,](#_bookmark53) [59,](#_bookmark60) [90,](#_bookmark91) [93,](#_bookmark93) |  |
|  | [121,](#_bookmark121) [142,](#_bookmark142) [162,](#_bookmark162) [171,](#_bookmark170) [185,](#_bookmark184) [186,](#_bookmark185) [213,](#_bookmark212) | - Texture: GLCM, GLRLM, LBP features, and color distribution |
|  | [222,](#_bookmark221) [240,](#_bookmark239) [28,](#_bookmark29) [65,](#_bookmark66) [66,](#_bookmark67) [207,](#_bookmark206) [243,](#_bookmark242) |  |
|  | [209,](#_bookmark208) [249,](#_bookmark248) [298,](#_bookmark297) [82,](#_bookmark83) [109]](#_bookmark109) | - Statistical: Histogram and moment-based |

Dimensionality Reduction

Hybrid Ap- proaches

Scale-Invariant Methods

**Statistical**: [[33]](#_bookmark34), [[213,](#_bookmark212) [28,](#_bookmark29) [65]](#_bookmark66)

**Morphological**: [[161,](#_bookmark161) [238,](#_bookmark237) [284,](#_bookmark283)

[304]](#_bookmark303), [[15,](#_bookmark16) [213,](#_bookmark212) [28,](#_bookmark29) [66,](#_bookmark67) [207,](#_bookmark206) [243,](#_bookmark242)

[298,](#_bookmark297) [109]](#_bookmark109)

**Density**: [[161,](#_bookmark161) [284]](#_bookmark283)

**PCA**: [[198]](#_bookmark197)

**NMF**: [[198]](#_bookmark197)

**STDA**: [[198]](#_bookmark197)

**YOLO+AMDF**: [[60]](#_bookmark61)

**FPN**: [[272]](#_bookmark271)

**Taxonomic indexes**: [[65]](#_bookmark66)

**Fuzzy entropy**: [[69]](#_bookmark70)

**LPL+CGL**: [[147]](#_bookmark147)

**SIDNMFE**: [[124,](#_bookmark124) [162]](#_bookmark162)

**SIFT-based**: [[124,](#_bookmark124) [162]](#_bookmark162)

- Morphological: Shape and structure
- Density: Spatial arrangement-based Feature reduction methods:
- PCA: Principal component analysis, a linear technique for determining the principal components or directions of maximum variance in data.
- NMF: Non-negative matrix factorization, commonly used for extracting interpretable and nonnegative features.
- STDA: Spatial-temporal discriminant analysis, extracts discriminative spatial-temporal features by maximizing differences between classes and minimizing variations within classes.

Combined techniques:

- YOLO+AMDF: Detection with multiscale fusion, combination of morphology and distribution features.
- FPN: Feature pyramid networks, pyramidal hierarchy of deep CNN to build feature pyramids at a low cost.
- Taxonomic: Phylogenetic tree approach providing significant insight into texture characteristics.
- Fuzzy entropy: Probability-based features for development of probability-based prevalent information set features.
- LPL+CGL: Hierarchical and geometric feature learning Scale-invariant feature extraction:
- SIDNMFE: Robust feature identification using SIFT concepts
- Combines morphological, texture, and density features
- Optimized for minimal processing time

Table S6: Supplementary Table S6: Various DL based classification techniques for breast lesion using TL models

| **Model Family** | **References** | **Description** |
| --- | --- | --- |
| ResNet Fam- | [[274,](#_bookmark273) [38,](#_bookmark39) [79,](#_bookmark80) [126,](#_bookmark126) [183,](#_bookmark182) [150,](#_bookmark150) [148,](#_bookmark148) [161,](#_bookmark161) [282,](#_bookmark281) [292,](#_bookmark291) [47]](#_bookmark48), | Utilizes skip connections and residual learning. |
| ily (ResNet, | [[270,](#_bookmark269) [129,](#_bookmark129) [7,](#_bookmark8) [86,](#_bookmark87) [87,](#_bookmark88) [230,](#_bookmark229) [58,](#_bookmark59) [10,](#_bookmark11) [12,](#_bookmark13) [21,](#_bookmark22) [23,](#_bookmark24) [22,](#_bookmark23) [54,](#_bookmark55) | ResNeXt adds cardinality for enhanced feature |
| ResNeXt) | [14,](#_bookmark15) [39,](#_bookmark40) [45,](#_bookmark46) [44,](#_bookmark45) [158,](#_bookmark158) [220,](#_bookmark219) [64,](#_bookmark65) [85,](#_bookmark86) [116,](#_bookmark116) [102,](#_bookmark102) [153,](#_bookmark153) [171,](#_bookmark170) | extraction through grouped convolutions |
|  | [173,](#_bookmark172) [130,](#_bookmark130) [181,](#_bookmark180) [197,](#_bookmark196) [203,](#_bookmark202) [297,](#_bookmark296) [94,](#_bookmark94) [234,](#_bookmark233) [303,](#_bookmark302) [4,](#_bookmark5) [26,](#_bookmark27) |  |
|  | [32,](#_bookmark33) [37,](#_bookmark38) [55,](#_bookmark56) [83,](#_bookmark84) [71,](#_bookmark72) [114,](#_bookmark114) [165,](#_bookmark165) [225,](#_bookmark224) [229,](#_bookmark228) [236,](#_bookmark235) [239,](#_bookmark238) |  |
|  | [248,](#_bookmark247) [265,](#_bookmark264) [298,](#_bookmark297) [267,](#_bookmark266) [108,](#_bookmark108) [187,](#_bookmark186) [107,](#_bookmark107) [168,](#_bookmark167) [76]](#_bookmark77) |  |
| Inception Fam- | [[38,](#_bookmark39) [183,](#_bookmark182) [150,](#_bookmark150) [161,](#_bookmark161) [181,](#_bookmark180) [292,](#_bookmark291) [47]](#_bookmark48), [[12,](#_bookmark13) [22,](#_bookmark23) [14,](#_bookmark15) [39,](#_bookmark40) | Progressive evolution from basic inception mod- |
| ily (GoogleNet, | [105,](#_bookmark105) [162,](#_bookmark162) [129,](#_bookmark129) [203,](#_bookmark202) [205,](#_bookmark204) [55,](#_bookmark56) [71,](#_bookmark72) [165,](#_bookmark165) [229,](#_bookmark228) [294,](#_bookmark293) [23,](#_bookmark24) | ules to hybrid architectures. Xception extends |
| Inception v1-v4, | [45,](#_bookmark46) [44,](#_bookmark45) [64,](#_bookmark65) [103,](#_bookmark103) [153,](#_bookmark153) [297,](#_bookmark296) [130,](#_bookmark130) [26,](#_bookmark27) [32,](#_bookmark33) [114,](#_bookmark114) [225,](#_bookmark224) | this with depthwise separable convolutions for |
| Inception- | [298,](#_bookmark297) [108,](#_bookmark108) [158,](#_bookmark158) [220,](#_bookmark219) [10,](#_bookmark11) [46,](#_bookmark47) [298,](#_bookmark297) [85,](#_bookmark86) [76]](#_bookmark77) | improved efficiency. |
| ResNet, Xcep- |  |  |
| tion) |  |  |
| Lightweight | [[156]](#_bookmark156), [[4,](#_bookmark5) [111,](#_bookmark111) [21,](#_bookmark22) [45,](#_bookmark46) [44,](#_bookmark45) [102,](#_bookmark102) [130,](#_bookmark130) [32,](#_bookmark33) [71,](#_bookmark72) [114,](#_bookmark114) | Optimized for mobile/edge devices using depth- |
| Networks | [225,](#_bookmark224) [262,](#_bookmark261) [168]](#_bookmark167) | wise separable convolutions and channel shuffling |
| (MobileNet, |  |  |
| SqueezeNet, |  |  |
| ShuffleNet) |  |  |
| EfficientNet | [[79,](#_bookmark80) [181,](#_bookmark180) [256]](#_bookmark255), [[147,](#_bookmark147) [218,](#_bookmark217) [154,](#_bookmark154) [197,](#_bookmark196) [297,](#_bookmark296) [26,](#_bookmark27) [32,](#_bookmark33) [46,](#_bookmark47) | Compound scaling method that uniformly scales |
|  | [114,](#_bookmark114) [117]](#_bookmark117) and others | network depth, width, and resolution |
| Classic CNNs | [[96,](#_bookmark96) [183,](#_bookmark182) [150,](#_bookmark150) [47,](#_bookmark48) [37,](#_bookmark38) [38,](#_bookmark39) [96,](#_bookmark96) [133,](#_bookmark133) [161,](#_bookmark161) [181,](#_bookmark180) [292]](#_bookmark291), | Traditional architectures that established funda- |
| (AlexNet, VGG, | [[231,](#_bookmark230) [22,](#_bookmark23) [45,](#_bookmark46) [44,](#_bookmark45) [105,](#_bookmark105) [127,](#_bookmark127) [153,](#_bookmark153) [174,](#_bookmark173) [241,](#_bookmark240) [273,](#_bookmark272) [191,](#_bookmark190) | mental CNN principles using stacked convolu- |
| LeNet) | [3,](#_bookmark4) [203,](#_bookmark202) [204,](#_bookmark203) [205,](#_bookmark204) [4,](#_bookmark5) [55,](#_bookmark56) [76,](#_bookmark77) [111,](#_bookmark111) [229,](#_bookmark228) [248,](#_bookmark247) [107,](#_bookmark107) [12,](#_bookmark13) | tional layers |
|  | [23,](#_bookmark24) [22,](#_bookmark23) [14,](#_bookmark15) [45,](#_bookmark46) [44,](#_bookmark45) [158,](#_bookmark158) [220,](#_bookmark219) [64,](#_bookmark65) [85,](#_bookmark86) [116,](#_bookmark116) [127,](#_bookmark127) [129,](#_bookmark129) |  |
|  | [297,](#_bookmark296) [94,](#_bookmark94) [303,](#_bookmark302) [4,](#_bookmark5) [26,](#_bookmark27) [32,](#_bookmark33) [39,](#_bookmark40) [55,](#_bookmark56) [83,](#_bookmark84) [114,](#_bookmark114) [118,](#_bookmark118) [165,](#_bookmark165) |  |
|  | [172,](#_bookmark171) [225,](#_bookmark224) [236,](#_bookmark235) [248,](#_bookmark247) [298,](#_bookmark297) [267,](#_bookmark266) [108,](#_bookmark108) [168]](#_bookmark167) |  |
| Dense Ar- | [[150,](#_bookmark150) [256]](#_bookmark255), [[12,](#_bookmark13) [23,](#_bookmark24) [14,](#_bookmark15) [45,](#_bookmark46) [44,](#_bookmark45) [144,](#_bookmark144) [199,](#_bookmark198) [297,](#_bookmark296) [130,](#_bookmark130) | Features dense connectivity pattern where each |
| chitectures | [4,](#_bookmark5) [26,](#_bookmark27) [32,](#_bookmark33) [111,](#_bookmark111) [114,](#_bookmark114) [225,](#_bookmark224) [229,](#_bookmark228) [248,](#_bookmark247) [298,](#_bookmark297) [294,](#_bookmark293) [107]](#_bookmark107) | layer connects to every other layer in a feed- |
| (DenseNet, | and others | forward fashion |
| DenseNet-II) |  |  |
| Modern Ar- | [[272]](#_bookmark271), [[251,](#_bookmark250) [21,](#_bookmark22) [100]](#_bookmark100) | Advanced architectures using capsules, neural ar- |
| chitectures |  | chitecture search, and focal loss, respectively |
| (CapsNet, Nas- |  |  |
| Net, RetinaNet) |  |  |

Table S7: Supplementary Table S7: Various DL-based classification techniques for breast lesions using DL models

| **Model Family** | **References** | **Description** |
| --- | --- | --- |
| Custom CNNs | [[96,](#_bookmark96) [122,](#_bookmark122) [269]](#_bookmark268), [[97,](#_bookmark97) [278,](#_bookmark277) | Various CNN architectures optimized for breast lesion classification, including |
|  | [28,](#_bookmark29) [66,](#_bookmark67) [8,](#_bookmark9) [142,](#_bookmark142) [266,](#_bookmark265) [15,](#_bookmark16) | dual-view, multi-task, cascaded approaches, Channel Boosted CNN, Local Binary |
|  | [90,](#_bookmark91) [127,](#_bookmark127) [116,](#_bookmark116) [17](#_bookmark18), [129,](#_bookmark129) | CNN (LBCNN), and backpropagation neural networks. Includes shallow CNNs |
|  | [180,](#_bookmark179) [129,](#_bookmark129) [139,](#_bookmark139) [75,](#_bookmark76) [80,](#_bookmark81) [98,](#_bookmark98) | for reduced complexity and deep variants for hierarchical feature learning |
|  | [264,](#_bookmark263) [24,](#_bookmark25) [140,](#_bookmark140) [44,](#_bookmark45) [78,](#_bookmark79) [73,](#_bookmark74) |  |
|  | [89,](#_bookmark90) [110,](#_bookmark110) [185,](#_bookmark184) [223,](#_bookmark222) [202,](#_bookmark201) |  |
|  | [253,](#_bookmark252) [16,](#_bookmark17) [27,](#_bookmark28) [209](#_bookmark208), [281,](#_bookmark280) |  |
|  | [285,](#_bookmark284) [286,](#_bookmark285) [306,](#_bookmark305) [305,](#_bookmark304) [210,](#_bookmark209) |  |
|  | [71,](#_bookmark72) [128,](#_bookmark128) [2,](#_bookmark3) [76]](#_bookmark77) |  |
| Object Detec- | [[242]](#_bookmark241), [[13,](#_bookmark14) [35,](#_bookmark36) [307,](#_bookmark306) [5,](#_bookmark6) | Networks like YOLO and Faster R-CNN combine region proposal with clas- |
| tion Networks | [113,](#_bookmark113) [216,](#_bookmark215) [239,](#_bookmark238) [13,](#_bookmark14) [36,](#_bookmark37) | sification. Faster R-CNN uses RPN for efficient region proposal |
|  | [307]](#_bookmark306) |  |
| Sequence Mod- | [[219,](#_bookmark218) [234,](#_bookmark233) [30,](#_bookmark31) [141,](#_bookmark141) [149,](#_bookmark149) | LSTM, Bi-LSTM, and RNN variants that process sequential data, capturing tem- |
| els | [185,](#_bookmark184) [186,](#_bookmark185) [223,](#_bookmark222) [29]](#_bookmark30) | poral dependencies in both forward and backward directions |
| Autoencoder- | [[33,](#_bookmark34) [161]](#_bookmark161), [[70,](#_bookmark71) [101]](#_bookmark101) | Various autoencoder architectures, including variational, deep extreme learning, |
| based |  | and standard autoencoders for feature learning and dimensionality reduction |
| Attention-based | [[11,](#_bookmark12) [250,](#_bookmark249) [282]](#_bookmark281)[[259,](#_bookmark258) [108,](#_bookmark108) | Networks incorporating attention mechanisms, including channel attention, multi- |
| Models | [302]](#_bookmark301) | frequency attention, and squeeze-excitation blocks for feature emphasis |

*Continued on next page*

Table S7 – continued from previous page

| **Model Family** | **References** | **Description** |
| --- | --- | --- |
| Transformer | [[287]](#_bookmark286), [[197,](#_bookmark196) [63]](#_bookmark64) | Visual Transformers (ViT) that process image patches as sequences, capturing |
| Models |  | both global and local features through self-attention mechanisms |
| Specialized Net- | [[238]](#_bookmark237), [[232,](#_bookmark231) [303,](#_bookmark302) [25,](#_bookmark26) [295]](#_bookmark294) | Purpose-built architectures like DCSNN (spiking neural networks), HFC (fuzzy |
| works |  | classifiers), FCE-Net (fine-grained cascade), Latent CAD X, and Deep random |
|  |  | vector functional link network (dRVFLN) |
| Graph-based | [[79,](#_bookmark80) [292]](#_bookmark291), [[306]](#_bookmark305) | Graph CNNs that process data as nodes and edges, effectively capturing relation- |
| Models |  | ships and dependencies in structured data |

Siamese Net- works

[[37]](#_bookmark38), [[290]](#_bookmark289) Architectures using twin networks with shared weights for similarity learning and feature comparison across images

Hybrid Models [[277]](#_bookmark276), [[137,](#_bookmark137) [252,](#_bookmark251) [138,](#_bookmark138) [18,](#_bookmark19)

[262]](#_bookmark261)

Combined architectures like Squeeze-U-Net, student-teacher networks, and federated learning approaches

Adversarial Models

[[184,](#_bookmark183) [279]](#_bookmark278), [[194,](#_bookmark193) [240]](#_bookmark239) GANs and domain adaptation networks that use adversarial training for improved feature learning and classification

Hybrid Models [[277]](#_bookmark276), [[137,](#_bookmark137) [252,](#_bookmark251) [138,](#_bookmark138) [18,](#_bookmark19)

[262,](#_bookmark261) [165]](#_bookmark165)

Combined architectures like Squeeze-U-Net, student-teacher networks, federated learning approaches, and Transferable texture CNN (TTCNN)

Deep Belief Net- works

Deep Residual Networks

Semi- Supervised Models

Wavelet Neural Networks Parasitic Metric Learning

[[9,](#_bookmark10) [41,](#_bookmark42) [59,](#_bookmark60) [235,](#_bookmark234) [164]](#_bookmark164) Multilayer neural networks with visible and hidden layers using restricted Boltz- mann machine (RBM) to train hierarchical representations based on the input data

[[192]](#_bookmark191) Networks with residual connections for learning residual mappings rather than desired underlying mapping, using a novel architecture with residual blocks

[[53,](#_bookmark54) [263]](#_bookmark262) Approaches combining labeled and unlabeled data training, including mixed self- and weakly supervised learning for improved understanding of dataset structure

[[84]](#_bookmark85) Networks combining wavelet analysis with neural networks for time-frequency feature extraction and pattern learning

[[120]](#_bookmark120) CNNs enhanced with metric learning layers for discriminative tissue description, utilizing the parasitic relationship between metric learning layers and traditional CNN structures

Table S8: Supplementary Table S8: Various ML-based breast lesion classification techniques

## Model Family References Description

**Multi-layer Per-** ceptron (MLP) / Fully Connected Network (FCN) Support Vector Machine (SVM)

[[62]](#_bookmark63) [[20,](#_bookmark21) [10,](#_bookmark11) [48,](#_bookmark49) [117,](#_bookmark117)

[205,](#_bookmark204) [201]](#_bookmark200)

[[160,](#_bookmark160) [304]](#_bookmark303) [[72,](#_bookmark73) [81,](#_bookmark82)

[115,](#_bookmark115) [106,](#_bookmark106) [228,](#_bookmark227) [227,](#_bookmark226)

[230,](#_bookmark229) [207]](#_bookmark206)

A deep neural network with multiple layers, where each neuron connects to all neurons in the next layer. Used for learning hierarchical features and classification.

A supervised learning model that finds an optimal hyperplane for classification in high-dimensional space. Works for binary and multi-class problems.

Random Forest [[304]](#_bookmark303) [[72,](#_bookmark73) [93,](#_bookmark93) [171,](#_bookmark170)

[222,](#_bookmark221) [228,](#_bookmark227) [227]](#_bookmark226)

Decision Tree [[72,](#_bookmark73) [228,](#_bookmark227) [227,](#_bookmark226) [300,](#_bookmark299)

[176]](#_bookmark175)

An ensemble learning technique that constructs multiple decision trees and combines their outputs to improve accuracy.

A tree-like model that splits data at each node based on feature values to arrive at a final classification.

## K-Nearest Neigh- bor (KNN)

[[96]](#_bookmark96) [[132,](#_bookmark132) [196,](#_bookmark195) [115,](#_bookmark115) A non-parametric classifier that assigns a data point to the most common class [171,](#_bookmark170) [199,](#_bookmark198) [228,](#_bookmark227) [227]](#_bookmark226) among its k-nearest neighbors.

## Logistic Regres- sion

Boosting Algo- rithms (Gradient Boost, XGBoost)

[[304]](#_bookmark303) [[146]](#_bookmark146) A statistical method used for binary classification based on a logistic function that models probabilities.

[[112]](#_bookmark112) [[222,](#_bookmark221) [249]](#_bookmark248) Ensemble methods that iteratively combine weak models (e.g., decision trees) to create a strong predictive model. XGBoost is an optimized version with better scalability and efficiency.

## Extreme Learning Machine (ELM)

[[284]](#_bookmark283) [[229,](#_bookmark228) [241,](#_bookmark240) [57,](#_bookmark58) A single hidden-layer neural network with randomly assigned weights, known for [109]](#_bookmark109) fast training and simplicity.

Naïve Bayes [[228,](#_bookmark227) [227,](#_bookmark226) [300]](#_bookmark299) A probabilistic classifier based on Bayes’ theorem, assuming feature independence

to simplify computations.

## Hannan Trans- form Classifier

[[69]](#_bookmark70) A classifier that employs entropy functions to evaluate and classify data with adaptive thresholding.

*Continued on next page*

Table S8 – continued from the previous page

**Techniques References Description**

Deep Feature- Spatially Local- ized Ensemble Sparse Analysis (DF-SLESA)

Rank R Feed- forward Neural Network (Rank-R FNN)

Neural Network

+ Logistic Re- gression Meta Learner

Adaptive Neuro- Fuzzy Classifier

[[104]](#_bookmark104) A method that uses sparse deep feature maps for classification, approximating unknown samples by dictionary learning.

[[271]](#_bookmark270) Uses tensor decomposition to efficiently capture spatial relationships in image data for classification.

[[175]](#_bookmark174) A hybrid model that combines neural networks and logistic regression to improve generalization across tasks.

[[208]](#_bookmark207) A model integrating fuzzy logic with neural networks to handle uncertainty and learn complex relationships in data.

Table S9: Supplementary Table S9: Various DL based breast density feature extraction techniques

| **Techniques** | **References** | **Description** |
| --- | --- | --- |
| Transfer Learning Models | [[51,](#_bookmark52) [214,](#_bookmark213) [285]](#_bookmark284) | Includes ResNet, Mo- |
|  |  | bileNet, EfficientNet, |
|  |  | and DenseNet architec- |
|  |  | tures |
| Traditional Feature Descriptors | [[66,](#_bookmark67) [213]](#_bookmark212) | Includes texture |
|  |  | (GLCM), statistical |
|  |  | (Histogram), morpho- |
|  |  | logical, shape, and color |
|  |  | features |
| Wavelet-based Features | [[214]](#_bookmark213) | Uses wavelet scattering |
|  |  | for feature extraction |

Table S10: Supplementary Table S10: Various DL based classification techniques for breast density using TL models

| **Techniques** | **References** | **Description** |
| --- | --- | --- |
| ResNet/EfficientNet Family | [[79,](#_bookmark80) [102,](#_bookmark102) [123,](#_bookmark123) [256]](#_bookmark255) | Includes ResNet and Ef- |
|  |  | ficientNet variants |
| MobileNet/DenseNet Family | [[102,](#_bookmark102) [123,](#_bookmark123) [256]](#_bookmark255) [[188]](#_bookmark187) | Includes MobileNet and |
|  |  | DenseNet architectures |
| Inception Family | [[123]](#_bookmark123) | Includes |
|  |  | GoogleNet/Inception V1 |
|  |  | and Inception-ResNet |
| Classic CNNs | [[102,](#_bookmark102) [123]](#_bookmark123) [[169,](#_bookmark168) [170]](#_bookmark169) | Includes AlexNet, VGG |
|  |  | Net, Xception, and Nas- |
|  |  | Net |

Table S11: Supplementary Table S11: Various DL based classification techniques for breast density using DL models

| **Techniques** | **References** | **Description** |
| --- | --- | --- |
| Custom CNN | [[145]](#_bookmark145), [[221,](#_bookmark220) [179,](#_bookmark178) [67,](#_bookmark68) [169,](#_bookmark168) [170](#_bookmark169), [66,](#_bookmark67) | Includes custom architectures for feature extraction and |
| Architectures | [75,](#_bookmark76) [51,](#_bookmark52) [188]](#_bookmark187) | classification, using convolutional layers for spatial fea- |
|  |  | tures, pooling layers for downsampling, and fully con- |
|  |  | nected layers for classification. Recent advances include |
|  |  | dual view CNN approaches [[51,](#_bookmark52) [188](#_bookmark187), [145].](#_bookmark145) |
| Advanced CNN | [[79,](#_bookmark80) [145]](#_bookmark145) [[157]](#_bookmark157) | Encompasses specialized architectures including: Graph |
| Variants |  | CNN (GCN) for graph-structured data processing, Di- |
|  |  | lated and Attention guided residual learning for enhanced |
|  |  | contextual information capture, and Confusion Matrix |
|  |  | CNN (CM-CNN) for handling multiple viewpoint evalua- |
|  |  | tions through transfer learning. |
| Weakly Super- | [[103,](#_bookmark103) [285]](#_bookmark284) | Training approach for ML models using noisy or partially |
| vised Learning |  | labeled data, enabling prediction with limited labeling |
|  |  | data while addressing the challenges of incomplete anno- |
|  |  | tations. |

Table S12: Supplementary Table S12: Various DL based classification techniques for breast density using ML models

| **Techniques** | **References** | **Description** |
| --- | --- | --- |
| Neural Network | [[267]](#_bookmark266) | Multi-layer Perceptron (MLP) and Fully Connected Net- |
| Approaches |  | work (FCN). |
| Traditional ML | [[213,](#_bookmark212) [275,](#_bookmark274) [214,](#_bookmark213) [267]](#_bookmark266) | Includes Support Vector Machine (SVM) and Random |
| Classifiers |  | Forest classifiers. |
| Nearest Neigh- | [[213,](#_bookmark212) [267]](#_bookmark266) | K-nearest Neighbor (KNN) classification based on major- |
| bor Methods |  | ity group of k-nearest neighbors. Includes weighted KNN |
|  |  | variants using kernel functions for point weighting [[213].](#_bookmark212) |
| Fuzzy Systems | [[275]](#_bookmark274) | Fuzzy inference systems like Fuzzy Takagi-Sugeno model |
|  |  | for mathematical analysis and classification, particularly |
|  |  | useful for image-derived inputs. Uses algorithmic input |
|  |  | calculation for increased precision without manual selec- |
|  |  | tion. |

Table S13: Supplementary Table S13: Image Pre-processing techniques used in the literature for noise reduction

| **Techniques** | **References** |
| --- | --- |
| Median Filter Family | [[83,](#_bookmark84) [60,](#_bookmark61) [104,](#_bookmark104) [1,](#_bookmark2) [6,](#_bookmark7) [16,](#_bookmark17) [41,](#_bookmark42) [46,](#_bookmark47) [48,](#_bookmark49) [80,](#_bookmark81) [117,](#_bookmark117) [160,](#_bookmark160) [58,](#_bookmark59) [230,](#_bookmark229) [163,](#_bookmark163) [186,](#_bookmark185) [220,](#_bookmark219) [219,](#_bookmark218) [233,](#_bookmark232) [234,](#_bookmark233) |
|  | [240,](#_bookmark239) [244,](#_bookmark243) [308,](#_bookmark307) [306,](#_bookmark305) [304,](#_bookmark303) [66,](#_bookmark67) [101,](#_bookmark101) [128]](#_bookmark128) |
| Gaussian/Mean Filter Family | [[183,](#_bookmark182) [143,](#_bookmark143) [160,](#_bookmark160) [161,](#_bookmark161) [220,](#_bookmark219) [49,](#_bookmark50) [48,](#_bookmark49) [104,](#_bookmark104) [84,](#_bookmark85) [89,](#_bookmark90) [116,](#_bookmark116) [98,](#_bookmark98) [112,](#_bookmark112) [305,](#_bookmark304) [65,](#_bookmark66) [155,](#_bookmark155) [162,](#_bookmark162) [284,](#_bookmark283) |
|  | [76,](#_bookmark77) [121]](#_bookmark121) |
| Advanced Filters | [[15,](#_bookmark16) [33,](#_bookmark34) [49,](#_bookmark50) [46,](#_bookmark47) [48,](#_bookmark49) [70,](#_bookmark71) [129,](#_bookmark129) [27,](#_bookmark28) [161,](#_bookmark161) [220,](#_bookmark219) [219,](#_bookmark218) [231,](#_bookmark230) [302,](#_bookmark301) [193,](#_bookmark192) [253,](#_bookmark252) [224]](#_bookmark223) |
| Specialized Techniques | [[52,](#_bookmark53) [59,](#_bookmark60) [230]](#_bookmark229) |

Table S14: Supplementary Table S14: Image Pre-processing techniques used in the literature for image enhancement

## Techniques References

Histogram-based Methods [[19,](#_bookmark20) [1,](#_bookmark2) [29,](#_bookmark30) [37,](#_bookmark38) [46,](#_bookmark47) [154,](#_bookmark154) [155,](#_bookmark155) [160,](#_bookmark160) [161,](#_bookmark161) [163,](#_bookmark163) [165,](#_bookmark165) [54,](#_bookmark55) [55,](#_bookmark56) [83,](#_bookmark84) [80,](#_bookmark81) [85,](#_bookmark86) [93,](#_bookmark93) [94,](#_bookmark94) [96,](#_bookmark96) [104,](#_bookmark104)

[172,](#_bookmark171) [111,](#_bookmark111) [118,](#_bookmark118) [127,](#_bookmark127) [218,](#_bookmark217) [6,](#_bookmark7) [16,](#_bookmark17) [27,](#_bookmark28) [33,](#_bookmark34) [36,](#_bookmark37) [76,](#_bookmark77) [103,](#_bookmark103) [112,](#_bookmark112) [114,](#_bookmark114) [183,](#_bookmark182) [143,](#_bookmark143) [169,](#_bookmark168) [244,](#_bookmark243) [219,](#_bookmark218)

[222,](#_bookmark221) [228,](#_bookmark227) [230,](#_bookmark229) [56,](#_bookmark57) [73]](#_bookmark74)

Contrast Enhancement Methods [[20,](#_bookmark21) [13,](#_bookmark14) [52,](#_bookmark53) [174,](#_bookmark173) [179,](#_bookmark178) [180,](#_bookmark179) [182,](#_bookmark181) [188,](#_bookmark187) [203,](#_bookmark202) [204,](#_bookmark203) [212,](#_bookmark211) [128,](#_bookmark128) [87]](#_bookmark88)

Intensity Transformation [[60,](#_bookmark61) [151,](#_bookmark151) [172,](#_bookmark171) [189,](#_bookmark188) [64,](#_bookmark65) [98]](#_bookmark98)

Advanced Enhancement Meth- ods

[[66,](#_bookmark67) [95,](#_bookmark95) [209,](#_bookmark208) [104,](#_bookmark104) [105,](#_bookmark105) [115,](#_bookmark115) [187,](#_bookmark186) [210,](#_bookmark209) [213,](#_bookmark212) [215,](#_bookmark214) [299,](#_bookmark298) [306,](#_bookmark305) [304]](#_bookmark303)

Table S15: Supplementary Table S15: Image Pre-processing techniques used in the literature for artifacts removal and region of interest selection

## Techniques References

Thresholding Methods [[306,](#_bookmark305) [304,](#_bookmark303) [56,](#_bookmark57) [58,](#_bookmark59) [264,](#_bookmark263) [121,](#_bookmark121) [205,](#_bookmark204) [243,](#_bookmark242) [9,](#_bookmark10) [182,](#_bookmark181) [48,](#_bookmark49) [219,](#_bookmark218) [257,](#_bookmark256) [160,](#_bookmark160) [119,](#_bookmark119) [276]](#_bookmark275)

Morphological Operations [[244,](#_bookmark243) [253,](#_bookmark252) [7,](#_bookmark8) [9,](#_bookmark10) [96,](#_bookmark96) [76,](#_bookmark77) [86,](#_bookmark87) [118,](#_bookmark118) [219,](#_bookmark218) [214,](#_bookmark213) [220,](#_bookmark219) [119,](#_bookmark119) [160,](#_bookmark160) [172,](#_bookmark171) [11,](#_bookmark12) [30,](#_bookmark31) [60]](#_bookmark61)

Region-based Methods [[6,](#_bookmark7) [98,](#_bookmark98) [143,](#_bookmark143) [151,](#_bookmark151) [157,](#_bookmark157) [172,](#_bookmark171) [214,](#_bookmark213) [221,](#_bookmark220) [96,](#_bookmark96) [306,](#_bookmark305) [304]](#_bookmark303)

ROI Extraction Methods [[301,](#_bookmark300) [130,](#_bookmark130) [255,](#_bookmark254) [263,](#_bookmark262) [278,](#_bookmark277) [283,](#_bookmark282) [89,](#_bookmark90) [91,](#_bookmark92) [297,](#_bookmark296) [20,](#_bookmark21) [14,](#_bookmark15) [30,](#_bookmark31) [37,](#_bookmark38) [61,](#_bookmark62) [75,](#_bookmark76) [289,](#_bookmark288) [251,](#_bookmark250) [261,](#_bookmark260) [296,](#_bookmark295)

[297]](#_bookmark296)

Edge Detection and Feature Methods

[[229,](#_bookmark228) [253,](#_bookmark252) [121,](#_bookmark121) [155,](#_bookmark155) [79,](#_bookmark80) [127](#_bookmark127)]

Specialized Filters [[6,](#_bookmark7) [29,](#_bookmark30) [46,](#_bookmark47) [87,](#_bookmark88) [51]](#_bookmark52)

# References

1. Dina Abdelhafiz, Jinbo Bi, Reda Ammar, Clifford Yang, and Sheida Nabavi. Convolutional neural network for automated mass segmentation in mammography. *BMC bioinformatics*, 21(1):1–19, 2020.
2. Hadeel Abdulmajeed and Mohammad Shkoukani. Mammogram image classification using local binary convolutional neural network. *Journal of Theoretical and Applied Information Technology*, 100(2):380 – 390, 2022.
3. Sarah S Aboutalib, Aly A Mohamed, Wendie A Berg, Margarita L Zuley, Jules H Sumkin, and Shandong Wu. Deep learning to distinguish recalled but benign mammography images in breast cancer screening. *Clinical Cancer Research*, 24(23):5902–5909, 2018.
4. Adeyinka P Adedigba, Steve A Adeshina, and Abiodun M Aibinu. Performance evaluation of deep learning models on mammogram classification using small dataset. *Bioengineering*, 9(4):161, 2022.
5. Richa Agarwal, Oliver Diaz, Moi Hoon Yap, Xavier Lladó, and Robert Marti. Deep learning for mass detection in full field digital mammograms. *Computers in biology and medicine*, 121:103774, 2020.
6. Jawad Ahmad, Sheeraz Akram, Arfan Jaffar, Muhammad Rashid, and Sohail Masood Bhatti. Breast cancer detection using deep learning: An investigation using the ddsm dataset and a customized alexnet and support vector machine. *IEEE Access*, 2023.
7. Luqman Ahmed, Muhammad Munwar Iqbal, Hamza Aldabbas, Shehzad Khalid, Yasir Saleem, and Saqib Saeed. Images data practices for semantic segmentation of breast cancer using deep neural network. *Journal of Ambient Intelligence and Humanized Computing*, pages 1–17, 2020.
8. Mugahed A Al-Antari, Mohammed A Al-Masni, Mun-Taek Choi, Seung-Moo Han, and Tae- Seong Kim. A fully integrated computer-aided diagnosis system for digital x-ray mammograms via deep learning detection, segmentation, and classification. *International journal of medical informatics*, 117:44–54, 2018.
9. Mugahed A Al-Antari, Mohammed A Al-Masni, Sung-Un Park, JunHyeok Park, Mohamed K Metwally, Yasser M Kadah, Seung-Moo Han, and Tae-Seong Kim. An automatic computer- aided diagnosis system for breast cancer in digital mammograms via deep belief network. *Journal of Medical and Biological Engineering*, 38:443–456, 2018.
10. Mugahed A Al-Antari, Seung-Moo Han, and Tae-Seong Kim. Evaluation of deep learning detection and classification towards computer-aided diagnosis of breast lesions in digital x- ray mammograms. *Computer methods and programs in biomedicine*, 196:105584, 2020.
11. Aymen M Al-Hejri, Riyadh M Al-Tam, Muneer Fazea, Archana Harsing Sable, Soojeong Lee, and Mugahed A Al-Antari. Etecadx: Ensemble self-attention transformer encoder for breast cancer diagnosis using full-field digital x-ray breast images. *Diagnostics*, 13(1):89, 2022.
12. Ebtihal Al-Mansour, Muhammad Hussain, and Hatim A Aboalsamh. An efficient method for breast mass classification using pre-trained deep convolutional networks. *Mathematics*, 10(14):2539, 2022.
13. Mohammed A Al-Masni, Mugahed A Al-Antari, Jeong-Min Park, Geon Gi, Tae-Yeon Kim, Patricio Rivera, Edwin Valarezo, Mun-Taek Choi, Seung-Moo Han, and Tae-Seong Kim. Si- multaneous detection and classification of breast masses in digital mammograms via a deep learning yolo-based cad system. *Computer methods and programs in biomedicine*, 157:85–94, 2018.
14. Riyadh M Al-Tam, Aymen M Al-Hejri, Sachin M Narangale, Nagwan Abdel Samee, Noha F Mahmoud, Mohammed A Al-Masni, and Mugahed A Al-Antari. A hybrid workflow of residual convolutional transformer encoder for breast cancer classification using digital x-ray mammo- grams. *Biomedicines*, 10(11):2971, 2022.
15. Umar Albalawi, S Manimurugan, and R Varatharajan. Classification of breast cancer mam- mogram images using convolution neural network. *Concurrency and computation: Practice and experience*, 34(13):e5803, 2022.
16. Mona Alfifi, Mohamad Shady Alrahhal, Samir Bataineh, and Mohammad Mezher. Enhanced artificial intelligence system for diagnosing and predicting breast cancer using deep learning. *International Journal of Advanced Computer Science and Applications*, 11(7):1–17, 2020.
17. Manal AlGhamdi and Mohamed Abdel-Mottaleb. Dv-dcnn: Dual-view deep convolutional neural network for matching detected masses in mammograms. *Computer methods and pro- grams in biomedicine*, 207:106152, 2021.
18. Manal AlGhamdi, Mohamed Abdel-Mottaleb, and Fernando Collado-Mesa. Du-net: Convolu- tional network for the detection of arterial calcifications in mammograms. *IEEE transactions on medical imaging*, 39(10):3240–3249, 2020.
19. Mohammad Alkhaleefah, Tan-Hsu Tan, Chuan-Hsun Chang, Tzu-Chuan Wang, Shang-Chih Ma, Lena Chang, and Yang-Lang Chang. Connected-segnets: A deep learning model for breast tumor segmentation from x-ray images. *Cancers*, 14(16):4030, 2022.
20. Ahad Alloqmani, Yoosef B Abushark, and Asif Irshad Khan. Anomaly detection of breast cancer using deep learning. *Arabian Journal for Science and Engineering*, pages 1–26, 2023.
21. Madallah Alruwaili and Walaa Gouda. Automated breast cancer detection models based on transfer learning. *Sensors*, 22(3):876, 2022.
22. Meteb M Altaf. A hybrid deep learning model for breast cancer diagnosis based on transfer learning and pulse-coupled neural networks. *Mathematical Biosciences and Engineering*, 18(5):5029–5046, 2021.
23. Ayman Altameem, Chandrakanta Mahanty, Ramesh Chandra Poonia, Abdul Khader Jilani Saudagar, and Raghvendra Kumar. Breast cancer detection in mammography images using deep convolutional neural networks and fuzzy ensemble modeling techniques. *Diagnostics*, 12(8):1812, 2022.
24. Gokhan Altan. Deep learning-based mammogram classification for breast cancer. *Interna- tional Journal of Intelligent Systems and Applications in Engineering*, 8(4):171–176, 2020.
25. Ulzee An, Ankit Bhardwaj, Khader Shameer, and Lakshminarayanan Subramanian. High precision mammography lesion identification from imprecise medical annotations. *Frontiers in big Data*, 4:742779, 2021.
26. Andres Anaya-Isaza, Leonel Mera-Jimenez, Johan Manuel Cabrera-Chavarro, Lorena Guachi- Guachi, Diego Peluffo-Ordonez, and Jorge Ivan Rios-Patino. Comparison of current deep convolutional neural networks for the segmentation of breast masses in mammograms. *IEEE Access*, 9:152206–152225, 2021.
27. Ridhi Arora, Prateek Kumar Rai, and Balasubramanian Raman. Deep feature–based au- tomatic classification of mammograms. *Medical & biological engineering & computing*, 58:1199–1211, 2020.
28. Charles Arputham, Krishnaraj Nagappan, Lenin Babu Russeliah, and AdalineSuji Russeliah. Mammographic image classification using deep neural network for computer-aided diagnosis. *Intelligent Automation & Soft Computing*, 27(3), 2021.
29. P Ashwini, N Suguna, and N Vadivelan. Improved bald eagle search optimization with entropy-based deep feature fusion model for breast cancer diagnosis on digital mammograms. *Multimedia Tools and Applications*, pages 1–19, 2023.
30. Muhammet Fatih Aslan. A hybrid end-to-end learning approach for breast cancer diagnosis: convolutional recurrent network. *Computers and Electrical Engineering*, 105:108562, 2023.
31. Alexander Augustyn, Sunati Sahoo, and Rachel D. Wooldridge. Large malignant phyllodes tumor of the breast with metastases to the lungs. *Rare Tumors*, 7(2), 2015.
32. Farnoosh Azour and Azzedine Boukerche. An efficient transfer and ensemble learning based computer aided breast abnormality diagnosis system. *IEEE Access*, 11:21199–21209, 2022.
33. Anu Babu and S Albert Jerome. Automatic breast cancer detection using hgmmem algorithm with delma classification. *Multimedia Tools and Applications*, pages 1–25, 2022.
34. Asma Baccouche, Begonya Garcia-Zapirain, Cristian Castillo Olea, and Adel S Elmaghraby. Connected-unets: a deep learning architecture for breast mass segmentation. *NPJ Breast Cancer*, 7(1):151, 2021.
35. Asma Baccouche, Begonya Garcia-Zapirain, Cristian Castillo Olea, and Adel S Elmaghraby. Breast lesions detection and classification via yolo-based fusion models. *Computers, Materials & Continua*, 69(1), 2021.
36. Asma Baccouche, Begonya Garcia-Zapirain, Yufeng Zheng, and Adel S Elmaghraby. Early detection and classification of abnormality in prior mammograms using image-to-image trans- lation and yolo techniques. *Computer Methods and Programs in Biomedicine*, 221:106884, 2022.
37. Jun Bai, Annie Jin, Tianyu Wang, Clifford Yang, and Sheida Nabavi. Feature fusion siamese network for breast cancer detection comparing current and prior mammograms. *Medical Physics*, 49(6):3654–3669, 2022.
38. Satish Babu Bandaru, Natarajasivan Deivarajan, and Rama Mohan Babu Gatram. An opti- mized deep learning techniques for analysing mammograms. *International Journal of Engi- neering Trends and Technology*, 70(7):388–398, 2022.
39. Satish Babu Bandaru, Natarajasivan Deivarajan, and Rama Mohan Babu Gatram. Investi- gations on deep learning techniques for analysing mammograms. *Revue d’Intelligence Arti- ficielle*, 36(3), 2022.
40. Peter John Bandura. Mastitis: Radiology reference article, Aug 2022. Accessed December 20, 2023.
41. Mohammed Basheri. Intelligent breast mass classification approach using archimedes opti- mization algorithm with deep learning on digital mammograms. *Biomimetics*, 8(6):463, 2023.
42. Daniel J Bell. Breast lipoma: Radiology reference article, Jun 2023. Accessed December 20, 2023.
43. Amparo S Betancourt Tarifa, Claudio Marrocco, Mario Molinara, Francesco Tortorella, and Alessandro Bria. Transformer-based mass detection in digital mammograms. *Journal of Ambient Intelligence and Humanized Computing*, 14(3):2723–2737, 2023.
44. Hemanta Kumar Bhuyan and Vinayakumar Ravi. An integrated framework with deep learning for segmentation and classification of cancer disease. *INTERNATIONAL JOURNAL ON ARTIFICIAL INTELLIGENCE TOOLS*, 32(02):2340002, 2023.
45. Hemanta Kumar Bhuyan, A Vijayaraj, and Vinayakumar Ravi. Diagnosis system for cancer disease using a single setting approach. *Multimedia Tools and Applications*, pages 1–27, 2023.
46. Saida Sarra Boudouh and Mustapha Bouakkaz. Breast cancer: new mammography dual-view classification approach based on pre-processing and transfer learning techniques. *Multimedia Tools and Applications*, pages 1–23, 2023.
47. Saida Sarra Boudouh and Mustapha Bouakkaz. Breast cancer: toward an accurate breast tumor detection model in mammography using transfer learning techniques. *Multimedia Tools and Applications*, pages 1–24, 2023.
48. Saida Sarra Boudouh and Mustapha Bouakkaz. Enhanced breast mass mammography clas- sification approach based on pre-processing and hybridization of transfer learning models. *Journal of Cancer Research and Clinical Oncology*, pages 1–16, 2023.
49. Saida Sarra Boudouh and Mustapha Bouakkaz. New enhanced breast tumor detection ap- proach in mammogram scans based on pre-processing and deep transfer learning techniques. *Multimedia Tools and Applications*, pages 1–22, 2023.
50. Brook Associates. Paget’s disease, n.d. Accessed December 22, 2023.
51. Mariam Busaleh, Muhammad Hussain, Hatim A Aboalsamh, and Sarah A Al Sultan. Twoviewdensitynet: Two-view mammographic breast density classification based on deep con- volutional neural network. *Mathematics*, 10(23):4610, 2022.
52. Xiuzhen Cai, Xia Li, Navid Razmjooy, Noradin Ghadimi, et al. Breast cancer diagnosis by convolutional neural network and advanced thermal exchange optimization algorithm. *Com- putational and Mathematical Methods in Medicine*, 2021, 2021.
53. Saul Calderon-Ramirez, Diego Murillo-Hernandez, Kevin Rojas-Salazar, David Elizondo, Shengxiang Yang, Armaghan Moemeni, and Miguel Molina-Cabello. A real use case of semi- supervised learning for mammogram classification in a local clinic of costa rica. *Medical & biological engineering & computing*, 60(4):1159–1175, 2022.
54. Haichao Cao, Shiliang Pu, Wenming Tan, Junyan Tong, and Di Zhang. Multi-tasking u-shaped network for benign and malignant classification of breast masses. *IEEE Access*, 8:223396– 223404, 2020.
55. Salvador Castro-Tapia, Celina Lizeth Castañeda-Miranda, Carlos Alberto Olvera-Olvera, Hec- tor A Guerrero-Osuna, José Manuel Ortiz-Rodriguez, Ma del Rosario Martinez-Blanco, Ger- mán Díaz-Florez, Jorge Domingo Mendiola-Santibañez, and Luis Octavio Solís-Sánchez. Clas- sification of breast cancer in mammograms with deep learning adding a fifth class. *Applied Sciences*, 11(23):11398, 2021.
56. SR Sannasi Chakravarthy, N Bharanidharan, and H Rajaguru. Deep learning-based meta- heuristic weighted k-nearest neighbor algorithm for the severity classification of breast cancer. *IRBM*, 44(3):100749, 2023.
57. SR Sannasi Chakravarthy, N Bharanidharan, and Harikumar Rajaguru. Processing of dig- ital mammogram images using optimized elm with deep transfer learning for breast cancer diagnosis. *Multimedia Tools and Applications*, pages 1–25, 2023.
58. SR Sannasi Chakravarthy and H Rajaguru. Automatic detection and classification of mam- mograms using improved extreme learning machine with deep learning. *Irbm*, 43(1):49–61, 2022.
59. Thirumarai Selvi Chandraraju and Amudha Jeyaprakash. Categorization of breast masses based on deep belief network parameters optimized using chaotic krill herd optimization al- gorithm for frequent diagnosis of breast abnormalities. *International Journal of Imaging Systems and Technology*, 32(5):1561–1576, 2022.
60. Jian-Ling Chen, Lan-Hsin Cheng, Jane Wang, Tun-Wei Hsu, Chin-Yu Chen, Ling-Ming Tseng, and Shu-Mei Guo. A yolo-based ai system for classifying calcifications on spot magnification mammograms. *BioMedical Engineering OnLine*, 22(1):54, 2023.
61. Juan Chen, Liangyong Chen, Shengsheng Wang, and Peng Chen. A novel multi-scale adver- sarial networks for precise segmentation of x-ray breast mass. *IEEE Access*, 8:103772–103781, 2020.
62. Qian-qian Chen, Shu-ting Lin, Jia-yi Ye, Yun-fei Tong, Shu Lin, and Si-qing Cai. Diagnos- tic value of mammography density of breast masses by using deep learning. *Frontiers in Oncology*, 13:1110657, 2023.
63. Xuxin Chen, Ke Zhang, Neman Abdoli, Patrik W Gilley, Ximin Wang, Hong Liu, Bin Zheng, and Yuchen Qiu. Transformers improve breast cancer diagnosis from unregistered multi-view mammograms. *Diagnostics*, 12(7):1549, 2022.
64. Hiba Chougrad, Hamid Zouaki, and Omar Alheyane. Deep convolutional neural networks for breast cancer screening. *Computer methods and programs in biomedicine*, 157:19–30, 2018.
65. Naveed Chouhan, Asifullah Khan, Jehan Zeb Shah, Mazhar Hussnain, and Muham- mad Waleed Khan. Deep convolutional neural network and emotional learning based breast cancer detection using digital mammography. *Computers in Biology and Medicine*, 132:104318, 2021.
66. Shaila Chugh, Sachin Goyal, Anjana Pandey, and Sunil Joshi. Morphological and otsu’s technique based mammography mass detection and deep neural network classifier based pre- diction. *Traitement du Signal*, 39(4), 2022.
67. Alexander Ciritsis, Cristina Rossi, Ilaria Vittoria De Martini, Matthias Eberhard, Magda Marcon, Anton S Becker, Nicole Berger, and Andreas Boss. Determination of mammographic breast density using a deep convolutional neural network. *The British journal of radiology*, 92(1093):20180691, 2019.
68. R. B. Cohen-Hallaleh, H. G. Smith, R. C. Smith, G. F. Stamp, O. Al-Muderis, K. Thway,

A. Miah, K. Khabra, I. Judson, R. Jones, and et al. Radiation induced angiosarcoma of the breast: Outcomes from a retrospective case series. *Clinical Sarcoma Research*, 7(1), 2017.

1. Jyoti Dabass, M Hanmandlu, and Rekha Vig. Formulation of probability-based pervasive in- formation set features and hanman transform classifier for the categorization of mammograms. *SN Applied Sciences*, 3(6):610, 2021.
2. J Dafni Rose, K VijayaKumar, Laxman Singh, and Sudhir Kumar Sharma. Computer-aided diagnosis for breast cancer detection and classification using optimal region growing segmen- tation with mobilenet model. *Concurrent Engineering*, 30(2):181–189, 2022.
3. Himanish Shekhar Das, Akalpita Das, Anupal Neog, Saurav Mallik, Kangkana Bora, and Zhongming Zhao. Breast cancer detection: Shallow convolutional neural network against deep convolutional neural networks based approach. *Frontiers in Genetics*, 13:1097207, 2023.
4. Maíra Araújo de Santana and Wellington Pinheiro dos Santos. A deep-wavelet neural network to detect and classify lesions in mammographic images. *Research on Biomedical Engineering*, 38(4):1051–1066, 2022.
5. Sagar Deep Deb, Aqhlaqur Rahman, and Rajib Kumar Jha. Breast cancer diagnosis using modified xception and stacked generalization ensemble classifier. *Research on Biomedical Engineering*, pages 1–11, 2023.
6. Mellanie Deborah. Fibroadenoma: Radiology case, Nov 2022. Accessed February 22, 2024.
7. Joao Otávio Bandeira Diniz, Pedro Henrique Bandeira Diniz, Thales Levi Azevedo Valente, Aristófanes Corrêa Silva, Anselmo Cardoso de Paiva, and Marcelo Gattass. Detection of mass regions in mammograms by bilateral analysis adapted to breast density using similarity indexes and convolutional neural networks. *Computer methods and programs in biomedicine*, 156:191–207, 2018.
8. M Diwakaran and D Surendran. Breast cancer prognosis based on transfer learning techniques in deep neural networks. *Information Technology and Control*, 52(2):381–396, 2023.
9. Bartosz Dolega-Kozierowski, Michal Lis, Hanna Marszalska-Jacak, Mateusz Koziej, Marcin Celer, Malgorzata Bandyk, Piotr Kasprzak, Bartlomiej Szynglarewicz, and Rafal Matkowski. Multimodality imaging in lobular breast cancer: Differences in mammography, ultrasound, and mri in the assessment of local tumor extent and correlation with molecular characteristics. *Frontiers in Oncology*, 12, 2022.
10. Andrea Duggento, Marco Aiello, Carlo Cavaliere, Giuseppe L Cascella, Davide Cascella, Gio- vanni Conte, Maria Guerrisi, Nicola Toschi, et al. An ad hoc random initialization deep neural network architecture for discriminating malignant breast cancer lesions in mammographic im- ages. *Contrast media & molecular imaging*, 2019, 2019.
11. Linh T Duong, Cong Q Chu, Phuong T Nguyen, Son T Nguyen, and Binh Q Tran. Edge detection and graph neural networks to classify mammograms: A case study with a dataset from vietnamese patients. *Applied Soft Computing*, 134:109974, 2023.
12. Enas MF El Houby and Nisreen IR Yassin. Malignant and nonmalignant classification of breast lesions in mammograms using convolutional neural networks. *Biomedical Signal Pro- cessing and Control*, 70:102954, 2021.
13. Ahmed S Elkorany and Zeinab F Elsharkawy. Efficient breast cancer mammograms diagnosis using three deep neural networks and term variance. *Scientific Reports*, 13(1):2663, 2023.
14. Abdelali Elmoufidi. Deep multiple instance learning for automatic breast cancer assessment using digital mammography. *IEEE transactions on instrumentation and measurement*, 71:1– 13, 2022.
15. Duygu Çelik Ertuğrul and Soona Ahmed Abdullah. A decision-making tool for early detection of breast cancer on mammographic images. *Tehnički vjesnik*, 29(5):1528–1536, 2022.
16. José Escorcia-Gutierrez, Romany F Mansour, Kelvin Beleño, Javier Jiménez-Cabas, Meglys Pérez, Natasha Madera, and Kevin Velasquez. Automated deep learning empowered breast cancer diagnosis using biomedical mammogram images. *Computers, Materials and Continua*, 71(3):3–4221, 2022.
17. Lenin G Falconi, Maria Perez, Wilbert G Aguilar, and Aura Conci. Transfer learning and fine tuning in breast mammogram abnormalities classification on cbis-ddsm database. *Adv. Sci. Technol. Eng. Syst. J*, 5(2):154–165, 2020.
18. Wael E Fathy and Amr S Ghoneim. A deep learning approach for breast cancer mass detection.

*International Journal of Advanced Computer Science and Applications*, 10(1), 2019.

1. Helen ML Frazer, Alex K Qin, Hong Pan, and Peter Brotchie. Evaluation of deep learning- based artificial intelligence techniques for breast cancer detection on mammograms: Results from a retrospective study using a breastscreen victoria dataset. *Journal of medical imaging and radiation oncology*, 65(5):529–537, 2021.
2. Xianjun Fu, Hao Cao, Hexuan Hu, Bobo Lian, Yansong Wang, Qian Huang, and Yirui Wu. Attention-based active learning framework for segmentation of breast cancer in mammograms. *Applied Sciences*, 13(2):852, 2023.
3. Fei Gao, Hyunsoo Yoon, Teresa Wu, and Xianghua Chu. A feature transfer enabled multi- task deep learning model on medical imaging. *Expert Systems with Applications*, 143:112957, 2020.
4. Norhène Gargouri, Raouia Mokni, Alima Damak, Dorra Sellami, and Riadh Abid. An auto- matic breast computer-aided diagnosis scheme based on a weighted fusion of relevant features and a deep cnn classifier. *IET Image Processing*, 16(12):3394–3406, 2022.
5. Lidia Garrucho, Kaisar Kushibar, Socayna Jouide, Oliver Diaz, Laura Igual, and Karim Lekadir. Domain generalization in deep learning based mass detection in mammography: A large-scale multi-center study. *Artificial Intelligence in Medicine*, 132:102386, 2022.
6. Lidia Garrucho, Kaisar Kushibar, Richard Osuala, Oliver Diaz, Alessandro Catanese, Javier Del Riego, Maciej Bobowicz, Fredrik Strand, Laura Igual, and Karim Lekadir. High-resolution synthesis of high-density breast mammograms: Application to improved fairness in deep learn- ing based mass detection. *Frontiers in Oncology*, 12:1044496, 2023.
7. Jayesh George Melekoodappattu, Anto Sahaya Dhas, Binil Kumar K, and KS Adarsh. Ma- lignancy detection on mammograms by integrating modified convolutional neural network classifier and texture features. *International Journal of Imaging systems and technology*, 32(2):564–574, 2022.
8. Alessia Gerbasi, Greta Clementi, Fabio Corsi, Sara Albasini, Alberto Malovini, Silvana Quaglini, and Riccardo Bellazzi. Deepmica: Automatic segmentation and classification of breast microcalcifications from mammograms. *Computer Methods and Programs in Biomedicine*, 235:107483, 2023.
9. Swarup Kr Ghosh and Anupam Ghosh. A novel hyperbolic intuitionistic fuzzy divergence measure based mammogram enhancement for visual elucidation of breast lesions. *Biomedical Signal Processing and Control*, 75:103586, 2022.
10. Vaira Suganthi Gnanasekaran, Sutha Joypaul, Parvathy Meenakshi Sundaram, and Durga Devi Chairman. Deep learning algorithm for breast masses classification in mam- mograms. *IET Image Processing*, 14(12):2860–2868, 2020.
11. Shuyue Guan and Murray Loew. Breast cancer detection using synthetic mammograms from generative adversarial networks in convolutional neural networks. *Journal of Medical Imag- ing*, 6(3):031411–031411, 2019.
12. Yuanfang Guan, Xueqing Wang, Hongyang Li, Zhenning Zhang, Xianghao Chen, Omer Sid- diqui, Sara Nehring, and Xiuzhen Huang. Detecting asymmetric patterns and localizing cancers on mammograms. *Patterns*, 1(7), 2020.
13. Naga Raju Gudhe, Hamid Behravan, Mazen Sudah, Hidemi Okuma, Ritva Vanninen, Veli- Matti Kosma, and Arto Mannermaa. Area-based breast percentage density estimation in mammograms using weight-adaptive multitask learning. *Scientific reports*, 12(1):12060, 2022.
14. Zeyad Q. Habeeb, Branislav Vuksanovic, and Imad Q. Al-Zaydi. Breast cancer detection using image processing and machine learning. *Journal of Image and Graphics*, 11(1):1–8, 2023.
15. Manar Ahmed Hamza. Hyperparameter tuned deep hybrid denoising autoencoder breast cancer classification on digital mammograms. *Intelligent Automation & Soft Computing*, 36(3), 2023.
16. Tengku Muhammad Hanis, Nur Intan Raihana Ruhaiyem, Wan Nor Arifin, Juhara Haron, Wan Faiziah Wan Abdul Rahman, Rosni Abdullah, and Kamarul Imran Musa. Developing a supplementary diagnostic tool for breast cancer risk estimation using ensemble transfer learning. *Diagnostics*, 13(10):1780, 2023.
17. Degan Hao, Lei Zhang, Jules Sumkin, Aly Mohamed, and Shandong Wu. Inaccurate labels in weakly-supervised deep learning: Automatic identification and correction and their impact on classification performance. *IEEE journal of biomedical and health informatics*, 24(9):2701– 2710, 2020.
18. Chelsea Harris, Uchenna Okorie, and Sokratis Makrogiannis. Spatially localized sparse ap- proximations of deep features for breast mass characterization. *Mathematical Biosciences and Engineering*, 20(9):15859–15882, 2023.
19. Shayma’a A Hassan, Mohammed S Sayed, Mahmoud I Abdalla, and Mohsen A Rashwan. Breast cancer masses classification using deep convolutional neural networks and transfer learning. *Multimedia Tools and Applications*, 79:30735–30768, 2020.
20. Asmaa A Hekal, Ahmed Elnakib, and Hossam El-Din Moustafa. Automated early breast cancer detection and classification system. *Signal, Image and Video Processing*, 15:1497– 1505, 2021.
21. Asmaa A Hekal, Hossam El-Din Moustafa, and Ahmed Elnakib. Ensemble deep learning system for early breast cancer detection. *Evolutionary Intelligence*, pages 1–10, 2022.
22. Iftikhar Hina, Shahid Ahmad Raza, Raza Basit, and Khan Hasan. Multi-view attention-based late fusion (mvalf) cadx system for breast cancer using deep learning. *Machine Graphics and Vision*, 29(1/4):55–78, 2020.
23. Hanan A Hosni Mahmoud, Amal H Alharbi, and Norah S Alghamdi. Breast cancer detection through feature clustering and deep learning. *Intelligent Automation & Soft Computing*, 31(2), 2022.
24. Essam H Houssein, Marwa M Emam, and Abdelmgeid A Ali. An optimized deep learning architecture for breast cancer diagnosis based on improved marine predators algorithm. *Neural Computing and Applications*, 34(20):18015–18033, 2022.
25. Mei-Ling Huang and Ting-Yu Lin. Considering breast density for the classification of benign and malignant mammograms. *Biomedical Signal Processing and Control*, 67:102564, 2021.
26. Nehad M Ibrahim, Batoola Ali, Fatimah Al Jawad, Majd Al Qanbar, Raghad I Aleisa, Sukainah A Alhmmad, Khadeejah R Alhindi, Mona Altassan, Afnan F Al-Muhanna, Hanoof M Algofari, et al. Breast cancer detection in the equivocal mammograms by aman method. *Applied Sciences*, 13(12):7183, 2023.
27. Bunyodbek Ibrokhimov and Justin-Youngwook Kang. Two-stage deep learning method for breast cancer detection using high-resolution mammogram images. *Applied Sciences*, 12(9):4616, 2022.
28. Adam Jaamour, Craig Myles, Ashay Patel, Shuen-Jen Chen, Lewis McMillan, and David Harris-Birtill. A divide and conquer approach to maximise deep learning mammography classification accuracies. *Plos one*, 18(5):e0280841, 2023.
29. Kiran Jabeen, Muhammad Attique Khan, Jamel Balili, Majed Alhaisoni, Nouf Abdullah Almujally, Huda Alrashidi, Usman Tariq, and Jae-Hyuk Cha. Bc2netrf: breast cancer classi- fication from mammogram images using enhanced deep learning features and equilibrium-jaya controlled regula falsi-based features selection. *Diagnostics*, 13(7):1238, 2023.
30. Saeid Jafarzadeh Ghoushchi, Ramin Ranjbarzadeh, Saeed Aghasoleimani Najafabadi, Elnaz Osgooei, and Erfan Babaee Tirkolaee. An extended approach to the diagnosis of tumour lo- cation in breast cancer using deep learning. *Journal of Ambient Intelligence and Humanized Computing*, pages 1–11, 2021.
31. Gul Shaira Banu Jahangeer and T Dhiliphan Rajkumar. Early detection of breast cancer using hybrid of series network and vgg-16. *Multimedia Tools and Applications*, 80:7853–7886, 2021.
32. G Jayandhi, JS Jasmine, and S Mary Joans. Mammogram learning system for breast cancer diagnosis using deep learning svm. *Computer Systems Science & Engineering*, 40(2), 2022.
33. Jiale Jiang, Junchuan Peng, Chuting Hu, Wenjing Jian, Xianming Wang, and Weixiang Liu. Breast cancer detection and classification in mammogram using a three-stage deep learning framework based on paa algorithm. *Artificial Intelligence in Medicine*, 134:102419, 2022.
34. Zhicheng Jiao, Xinbo Gao, Ying Wang, and Jie Li. A parasitic metric learning net for breast mass classification based on mammography. *Pattern Recognition*, 75:292–301, 2018.
35. Meredith A Jones, Negar Sadeghipour, Xuxin Chen, Warid Islam, and Bin Zheng. A multi- stage fusion framework to classify breast lesions using deep learning and radiomics features computed from four-view mammograms. *Medical Physics*, 2023.
36. Annie Julie Joseph, Priyansh Dwivedi, Jiffy Joseph, Seenia Francis, PN Pournami, PB Ja- yaraj, Ashna V Shamsu, and Praveen Sankaran. Prior-guided generative adversarial network for mammogram synthesis. *Biomedical Signal Processing and Control*, 87:105456, 2024.
37. Eman Justaniah, Ghadah Aldabbagh, Areej Alhothali, and Nesreen Abourokbah. Classify- ing breast density from mammogram with pretrained cnns and weighted average ensembles. *Applied Sciences*, 12(11):5599, 2022.
38. Sai Krishna K and Grace Kanmani P. Scale invariant deep neural multiple feature learning based boosted support vector entropy classification for breast cancer diagnosis using mammo- grams. *International Journal of Electrical and Electronics Engineering*, 10(8):79–88, Sep 2023.
39. Ayla Al Kabbani. Breast abscess: Radiology case, Nov 2022. Accessed December 20, 2023.
40. Mahmoud Shiri Kahnouei, Masoumeh Giti, Mohammad Ali Akhaee, and Ali Ameri. Micro- calcification detection in mammograms using deep learning. *Iranian Journal of Radiology*, 19(1), 2022.
41. R Karthiga, K Narasimhan, and Rengarajan Amirtharajan. Diagnosis of breast cancer for modern mammography using artificial intelligence. *Mathematics and Computers in Simula- tion*, 202:316–330, 2022.
42. T Kavitha, Paul P Mathai, C Karthikeyan, M Ashok, Rachna Kohar, J Avanija, and S Nee- lakandan. Deep learning based capsule neural network model for breast cancer diagnosis using mammogram images. *Interdisciplinary Sciences: Computational Life Sciences*, pages 1–17, 2021.
43. Hasan Nasir Khan, Ahmad Raza Shahid, Basit Raza, Amir Hanif Dar, and Hani Alquhayz. Multi-view feature fusion based four views model for mammogram classification using convo- lutional neural network. *IEEE Access*, 7:165724–165733, 2019.
44. Rimsha Khan and Giovanni Luca Masala. Detecting breast arterial calcifications in mammo- grams with transfer learning. *Electronics*, 12(1):231, 2023.
45. Rohan Khandelwal. Phyllodes tumor, Apr 2017. Accessed December 22, 2024.
46. Priyanka Khanna, Mridu Sahu, Bikesh Kumar Singh, and Vikrant Bhateja. Combining mod- ified hyper learning binary dragonfly algorithm and deep learning for bi-rads classification of breast masses in mammograms. *Expert Systems*, page e13200, 2022.
47. Young Jae Kim and Kwang Gi Kim. Detection and weak segmentation of masses in gray- scale breast mammogram images using deep learning. *Yonsei Medical Journal*, 63(Suppl):S63, 2022.
48. Henry Knipe. Simple breast cyst: Radiology case, Nov 2022. Accessed February 22, 2024.
49. Miglena K. Komforti and Bryan E. Harmon. Educational case: Ductal carcinoma in situ (dcis). *Academic Pathology*, 6:2374289519888727, 2019.
50. Garth Kruger. Paget disease of the breast: Radiology case, Nov 2022. Accessed December 22, 2023.
51. Sujata Kulkarni and Rinku Rabidas. Fully convolutional network for automated detection and diagnosis of mammographic masses. *Multimedia Tools and Applications*, pages 1–22, 2023.
52. Sujata Kulkarni and Rinku Rabidas. Squeezeu-net-based detection and diagnosis of micro- calcification in mammograms. *Signal, Image and Video Processing*, 17(2):435–443, 2023.
53. Indrajeet Kumar and Rashmi Gudur. Machine learning approaches for automatic lesion detec- tion in mammography images. *International Journal of Intelligent Systems and Applications in Engineering*, 11(7s):91–96, 2023.
54. Koushlendra Kumar Singh, Suraj Kumar, Marios Antonakakis, Konstantina Moirogiorgou, Anirudh Deep, Kanchan Lata Kashyap, Manish Kumar Bajpai, and Michalis Zervakis. Deep learning capabilities for the categorization of microcalcification. *International Journal of Environmental Research and Public Health*, 19(4):2159, 2022.
55. Savita Kumbhare, Atul B Kathole, and Swati Shinde. Federated learning aided breast can- cer detection with intelligent heuristic-based deep learning framework. *Biomedical Signal Processing and Control*, 86:105080, 2023.
56. Jarosław Kurek, Bartosz Świderski, Stanisław Osowski, Michał Kruk, and Walid Barhoumi. Deep learning versus classical neural approach to mammogram recognition. *Bulletin of the Polish Academy of Sciences, Technical Sciences*, 66(6), 2018.
57. Andrés Larroza, Francisco Javier Pérez-Benito, Juan-Carlos Perez-Cortes, Marta Román, Marina Pollán, Beatriz Pérez-Gómez, Dolores Salas-Trejo, María Casals, and Rafael Llobet. Breast dense tissue segmentation with noisy labels: A hybrid threshold-based and mask-based approach. *Diagnostics*, 12(8):1822, 2022.
58. Rebecca Sawyer Lee, Jared A Dunnmon, Ann He, Siyi Tang, Christopher Re, and Daniel L Rubin. Comparison of segmentation-free and segmentation-dependent computer-aided diag- nosis of breast masses on a public mammography dataset. *Journal of biomedical informatics*, 113:103656, 2021.
59. Cheng Li, Jingxu Xu, Qiegen Liu, Yongjin Zhou, Lisha Mou, Zuhui Pu, Yong Xia, Hairong Zheng, and Shanshan Wang. Multi-view mammographic density classification by dilated and attention-guided residual learning. *IEEE/ACM transactions on computational biology and bioinformatics*, 18(3):1003–1013, 2020.
60. Dong Li, Lei Zhang, Jianwei Zhang, and Xingyu Xie. Convolutional feature descriptor selec- tion for mammogram classification. *IEEE Journal of Biomedical and Health Informatics*, 27(3):1467–1476, 2023.
61. Heyi Li, Dongdong Chen, William H Nailon, Mike E Davies, and David I Laurenson. Dual convolutional neural networks for breast mass segmentation and diagnosis in mammography. *IEEE Transactions on Medical Imaging*, 41(1):3–13, 2021.
62. Hongmei Li, Jing Ye, Hao Liu, Yichuan Wang, Binbin Shi, Juan Chen, Aiping Kong, Qing Xu, and Junhui Cai. Application of deep learning in the detection of breast lesions with four different breast densities. *Cancer Medicine*, 10(14):4994–5000, 2021.
63. Hua Li, Jing Niu, Dengao Li, and Chen Zhang. Classification of breast mass in two-view mammograms via deep learning. *IET Image Processing*, 15(2):454–467, 2021.
64. Hua Li, Shasha Zhuang, Deng-ao Li, Jumin Zhao, and Yanyun Ma. Benign and malignant classification of mammogram images based on deep learning. *Biomedical Signal Processing and Control*, 51:347–354, 2019.
65. Shuyi Li, Min Dong, Guangming Du, and Xiaomin Mu. Attention dense-u-net for automatic breast mass segmentation in digital mammogram. *IEEE Access*, 7:59037–59047, 2019.
66. Yamei Li, Guohua Zhao, Qian Zhang, Yusong Lin, and Meiyun Wang. Sap-cgan: Adversarial learning for breast mass segmentation in digital mammogram based on superpixel average pooling. *Medical Physics*, 48(3):1157–1167, 2021.
67. Rong-Ho Lin, Benjamin Kofi Kujabi, Chun-Ling Chuang, Ching-Shun Lin, and Chun-Jen Chiu. Application of deep learning to construct breast cancer diagnosis model. *Applied Sciences*, 12(4):1957, 2022.
68. Vandana Lingampally and Radhika Kavuri. Combining super resolution and efficient net models to reduce false positives and false negatives in breast cancer detection. *International Journal of Engineering Trends and Technology*, 71(5):386–401, May 2023.
69. Dongdong Liu, Bo Wu, Changbo Li, Zheng Sun, and Nan Zhang. Trend: A transformer-based encoder-decoder model with adaptive patch embedding for mass segmentation in mammo- grams. *Medical Physics*, 50(5):2884–2899, 2023.
70. Huanhuan Liu, Yanhong Chen, Yuzhen Zhang, Lijun Wang, Ran Luo, Haoting Wu, Chenqing Wu, Huiling Zhang, Weixiong Tan, Hongkun Yin, et al. A deep learning model integrating mammography and clinical factors facilitates the malignancy prediction of bi-rads 4 micro- calcifications in breast cancer screening. *European Radiology*, 31:5902–5912, 2021.
71. Hector Lopez-Almazan, Francisco Javier Pérez-Benito, Andrés Larroza, Juan-Carlos Perez- Cortes, Marina Pollan, Beatriz Perez-Gomez, Dolores Salas Trejo, María Casals, and Rafael Llobet. A deep learning framework to classify breast density with noisy labels regularization. *Computer Methods and Programs in Biomedicine*, 221:106885, 2022.
72. José Daniel López-Cabrera, Luis Alberto López Rodríguez, and Marlén Pérez-Díaz. Classifica- tion of breast cancer from digital mammography using deep learning. *Inteligencia Artificial*, 23(65):56–66, 2020.
73. Meng Lou, Yunliang Qi, Jie Meng, Chunbo Xu, Yiming Wang, Jiande Pi, and Yide Ma. Dcanet: Dual contextual affinity network for mass segmentation in whole mammograms. *Medical Physics*, 48(8):4291–4303, 2021.
74. Tariq Mahmood, Jianqiang Li, Yan Pei, and Faheem Akhtar. An automated in-depth fea- ture learning algorithm for breast abnormality prognosis and robust characterization from mammography images using deep transfer learning. *Biology*, 10(9):859, 2021.
75. Tariq Mahmood, Jianqiang Li, Yan Pei, Faheem Akhtar, Mujeeb Ur Rehman, and Shah- baz Hassan Wasti. Breast lesions classifications of mammographic images using a deep con- volutional neural network-based approach. *Plos one*, 17(1):e0263126, 2022.
76. Hanan A Hosni Mahmoud, Amal H Alharbi, and Doaa S Khafga. Breast cancer classification using deep convolution neural network with transfer learning. *Intelligent Automation & Soft Computing*, 29(3), 2021.
77. P Malathi and G Latha. Classification of multi-view digital mammogram images using smo- wknn. *Computer Systems Science & Engineering*, 46(2), 2023.
78. Areej A Malibari, Mohamed K Nour, Amal S Mehanna, Manar Ahmed Hamza, Abu Sarwar Zamani, Ishfaq Yaseen, Abdelwahed Motwakel, et al. Gaussian optimized deep learning-based belief classification model for breast cancer detection. *Computers, Materials & Continua*, 73(2), 2022.
79. Sarmad Maqsood, Robertas Damaševičius, and Rytis Maskeliu¯nas. Ttcnn: A breast cancer detection and classification towards computer-aided diagnosis using digital mammography in early stages. *Applied Sciences*, 12(7):3273, 2022.
80. Mayo Clinic. Dense breast tissue may increase the risk of breast cancer, n.d. Accessed December 22, 2023.
81. Medjeded Merati, Saïd Mahmoudi, Abdelkader Chenine, and Mohamed A Chikh. A new triplet convolutional neural network for classification of lesions on mammograms. *Revue d’Intelligence Artificielle*, 33(3), 2019.
82. Nada Mobark, Safwat Hamad, and SZ Rida. Coronet: Deep neural network-based end-to-end training for breast cancer diagnosis. *Applied Sciences*, 12(14):7080, 2022.
83. Aly A Mohamed, Wendie A Berg, Hong Peng, Yahong Luo, Rachel C Jankowitz, and Shan- dong Wu. A deep learning method for classifying mammographic breast density categories. *Medical physics*, 45(1):314–321, 2018.
84. Aly A Mohamed, Yahong Luo, Hong Peng, Rachel C Jankowitz, and Shandong Wu. Un- derstanding clinical mammographic breast density assessment: a deep learning perspective. *Journal of digital imaging*, 31:387–392, 2018.
85. Raouia Mokni and Mariem Haoues. Cadnet157 model: fine-tuned resnet152 model for breast cancer diagnosis from mammography images. *Neural Computing and Applications*, 34(24):22023–22046, 2022.
86. Sidratul Montaha, Sami Azam, Abul Kalam Muhammad Rakibul Haque Rafid, Pronab Ghosh, Md Zahid Hasan, Mirjam Jonkman, and Friso De Boer. Breastnet18: A high accuracy fine- tuned vgg16 model evaluated using ablation study for diagnosing breast cancer from enhanced mammography images. *Biology*, 10(12):1347, 2021.
87. Vicky Mudeng, Jin-woo Jeong, and Se-woon Choe. Simply fine-tuned deep learning-based classification for breast cancer with mammograms. *Computers, Materials & Continua*, 73(3), 2022.
88. K Lakshmi Narayanan, R Santhana Krishnan, and Y Harold Robinson. A hybrid deep learning based assist system for detection and classification of breast cancer from mammogram images. *International Arab Journal of Information Technology*, 19(6), 2022.
89. Varsha Nemade, Sunil Pathak, and Ashutosh Kumar Dubey. Deep learning-based ensemble model for classification of breast cancer. *Microsystem Technologies*, pages 1–15, 2023.
90. Varsha Nemade, Sunil Pathak, and Ashutosh Kumar Dubey. Hybrid deep convolutional neural network approach for detecting breast cancer in mammography images. *International Journal of Electrical and Electronics Engineering*, 10(5):102–119, 2023.
91. Mohammadtaghi Niknejad. Fat necrosis of the breast: Radiology case, Nov 2022. Accessed December 20, 2023.
92. Mohammadtaghi Niknejad. Intraductal papilloma of breast: Radiology reference article, Nov 2022. Accessed December 20, 2023.
93. R Nithya and B Santhi. Mammogram density classification using deep convolutional neural network. *Journal of Instrumentation*, 16(01):P01019, 2021.
94. Olaide N Oyelade and Absalom E Ezugwu. A novel wavelet decomposition and transformation convolutional neural network with data augmentation for breast cancer detection using digital mammogram. *Scientific Reports*, 12(1):5913, 2022.
95. Parita Oza, Paawan Sharma, and Samir Patel. Deep ensemble transfer learning-based frame- work for mammographic image classification. *The Journal of Supercomputing*, 79(7):8048– 8069, 2023.
96. Parita Rajiv Oza, Paawan Sharma, and Samir Patel. Transfer learning assisted classification of artefacts removed and contrast improved digital mammograms. *Scalable Computing: Practice and Experience*, 23(3):115–127, 2022.
97. Pratheep Kumar P and Mary Amala V. Breast cancer detection on mammographic im- ages using hyper parameter tuning and optimization: A convolutional neural network and transfer learning approach. *International Journal of Engineering Trends and Technology*, 70(9):79–92, Sep 2022.
98. Seungju Park, Kyung Hwa Lee, Beomseok Ko, and Namkug Kim. Unsupervised anomaly de- tection with generative adversarial networks in mammography. *Scientific Reports*, 13(1):2925, 2023.
99. Rajeshwari S Patil and Nagashettappa Biradar. Automated mammogram breast cancer de- tection using the optimized combination of convolutional and recurrent neural network. *Evo- lutionary intelligence*, 14:1459–1474, 2021.
100. Rajeshwari S Patil, Nagashettappa Biradar, and Rashmi Pawar. A new automated seg- mentation and classification of mammogram images. *Multimedia Tools and Applications*, 81(6):7783–7816, 2022.
101. Satyabrata Patro, Jyotirmaya Mishra, and Bhavani Sankar Panda. Hybrid convolutional neural network with residual neural network for breast cancer prediction using mammography images. *International Journal of Intelligent Engineering & Systems*, 16(1), 2023.
102. Shivaji D Pawar, Kamal K Sharma, Suhas G Sapate, Geetanjali Y Yadav, Roobaea Alroobaea, Sabah M Alzahrani, and Mustapha Hedabou. Multichannel densenet architecture for clas- sification of mammographic breast density for breast cancer detection. *Frontiers in Public Health*, 10:885212, 2022.
103. Shivaji D Pawar, Kamal Kr Sharma, Suhas G Sapate, and Geetanjali Y Yadav. Segmentation of pectoral muscle from digital mammograms with depth-first search algorithm towards breast density classification. *Biocybernetics and Biomedical Engineering*, 41(3):1224–1241, 2021.
104. Francisco Javier Pérez-Benito, François Signol, Juan-Carlos Perez-Cortes, Alejandro Fuster- Baggetto, Marina Pollan, Beatriz Pérez-Gómez, Dolores Salas-Trejo, Maria Casals, Inmacu- lada Martínez, and Rafael LLobet. A deep learning system to obtain the optimal parameters for a threshold-based breast and dense tissue segmentation. *Computer Methods and Programs in Biomedicine*, 195:105668, 2020.
105. Filippo Pesapane, Chiara Trentin, Federica Ferrari, Giulia Signorelli, Priyan Tantrige, Marta Montesano, Crispino Cicala, Roberto Virgoli, Silvia D’Acquisto, Luca Nicosia, et al. Deep learning performance for detection and classification of microcalcifications on mammography. *European Radiology Experimental*, 7(1):69, 2023.
106. Rajasree PM, Anand Jatti, and Dr Divya Santosh. An improved transfer learning approach towards breast cancer classification on deep residual network. *Indian Journal of Computer Science and Engineering*, 12(4):1136–1148, 2021.
107. Anitha Ponraj and R Aroul Canessane. Deep learning with histogram of oriented gradients- based computer-aided diagnosis for breast cancer detection and classification. In *2023 3rd International Conference on Smart Data Intelligence (ICSMDI)*, pages 527–532. IEEE, 2023.
108. Anitha Ponraj and R Aroul Canessane. Generative adversarial networks with modified wasp swarm algorithm-based early-stage breast cancer detection techniques. *Soft Computing*, pages 1–13, 2023.
109. Nikolaos Poulianitis. Primary breast angiosarcoma: Case report. *Primary breast angiosar- coma: Case report*, 2016. Accessed December 23, 2023.
110. Payel Pramanik, Souradeep Mukhopadhyay, Seyedali Mirjalili, and Ram Sarkar. Deep fea- ture selection using local search embedded social ski-driver optimization algorithm for breast cancer detection in mammograms. *Neural Computing and Applications*, 35(7):5479–5499, 2023.
111. Marcel Prodan, Elena Paraschiv, and Alexandru Stanciu. Applying deep learning methods for mammography analysis and breast cancer detection. *Applied Sciences*, 13(7):4272, 2023.
112. Y Qiu, G Zhou, Q Zhao, and A Cichocki. Comparative study on the classification methods for breast cancer diagnosis. *Bulletin of the Polish Academy of Sciences. Technical Sciences*, 66(6), 2018.
113. Jinrong Qu, Xuran Zhao, Peng Chen, Zhaoqi Wang, Zhenzhen Liu, Bailin Yang, and Hailiang Li. Deep learning on digital mammography for expert-level diagnosis accuracy in breast cancer detection. *Multimedia Systems*, pages 1–12, 2022.
114. Alejandro Ernesto Quiñones-Espín, Marlen Perez-Diaz, Rafaela Mayelín Espín-Coto, Deijany Rodriguez-Linares, and José Daniel Lopez-Cabrera. Automatic detection of breast masses using deep learning with yolo approach. *Health and Technology*, pages 1–9, 2023.
115. Gonzalo Iñaki Quintana, Zhijin Li, Laurence Vancamberg, Mathilde Mougeot, Agnès Desol- neux, and Serge Muller. Exploiting patch sizes and resolutions for multi-scale deep learning in mammogram image classification. *Bioengineering*, 10(5):534, 2023.
116. R Sathesh Raaj. Breast cancer detection and diagnosis using hybrid deep learning architec- ture. *Biomedical Signal Processing and Control*, 82:104558, 2023.
117. Dina A Ragab, Omneya Attallah, Maha Sharkas, Jinchang Ren, and Stephen Marshall. A framework for breast cancer classification using multi-dcnns. *Computers in Biology and Medicine*, 131:104245, 2021.
118. Dina A. Ragab, Maha Sharkas, Stephen Marshall, and Jinchang Ren. Breast cancer detection using deep convolutional neural networks and support vector machines. *PeerJ*, 7, 2019.
119. R Rajakumari and L Kalaivani. Breast cancer detection and classification using deep cnn techniques. *Intelligent Automation & Soft Computing*, 32(2), 2022.
120. N Ravitha Rajalakshmi, R Vidhyapriya, N Elango, and Nikhil Ramesh. Deeply supervised u-net for mass segmentation in digital mammograms. *International Journal of Imaging Systems and Technology*, 31(1):59–71, 2021.
121. S Ramesh, S Sasikala, S Gomathi, V Geetha, and V Anbumani. Segmentation and clas- sification of breast cancer using novel deep learning architecture. *Neural Computing and Applications*, 34(19):16533–16545, 2022.
122. Jyoti Rani, Jaswinder Singh, and Jitendra Virmani. Hybrid computer aided diagnostic sys- tem designs for screen film mammograms using dl-based feature extraction and ml-based classifiers. *Expert Systems*, page e13309, 2023.
123. Ramin Ranjbarzadeh, Saeid Jafarzadeh Ghoushchi, Nazanin Tataei Sarshar, Erfan Babaee Tirkolaee, Sadia Samar Ali, Teerath Kumar, and Malika Bendechache. Me-ccnn: Multi- encoded images and a cascade convolutional neural network for breast tumor segmentation and recognition. *Artificial Intelligence Review*, pages 1–38, 2023.
124. Ramin Ranjbarzadeh, Nazanin Tataei Sarshar, Saeid Jafarzadeh Ghoushchi, Mohammad Saleh Esfahani, Mahboub Parhizkar, Yaghoub Pourasad, Shokofeh Anari, and Malika Ben- dechache. Mrfe-cnn: Multi-route feature extraction model for breast tumor segmentation in mammograms using a convolutional neural network. *Annals of Operations Research*, 328(1):1021–1042, 2023.
125. Bahman Rasuli. Breast hamartoma: Radiology case, Jun 2023. Accessed December 20, 2023.
126. M Ravikumar, PG Rachana, and BJ Shivaprasad. Segmentation of tumour from mammo- gram images using u-segnet: a hybrid approach. *Computer Methods in Biomechanics and Biomedical Engineering: Imaging & Visualization*, 2023.
127. Noor Fadzilah Razali, Iza Sazanita Isa, Siti Noraini Sulaiman, Noor Khairiah Abdul Karim, Muhammad Khusairi Osman, and Zainal Hisham Che Soh. Enhancement technique based on the breast density level for mammogram for computer-aided diagnosis. *Bioengineering*, 10(2):153, 2023.
128. Noor Fadzilah Razali, Iza Sazanita Isa, Siti Noraini Sulaiman, Noor Khairiah A Karim, and Muhammad Khusairi Osman. Cnn-wavelet scattering textural feature fusion for classifying breast tissue in mammograms. *Biomedical Signal Processing and Control*, 83:104683, 2023.
129. Shams ur Rehman, Muhamamd Attique Khan, Anum Masood, Nouf Abdullah Almujally, Jamel Baili, Majed Alhaisoni, Usman Tariq, and Yu-Dong Zhang. Brmi-net: Deep learning features and flower pollination-controlled regula falsi-based feature selection framework for breast cancer recognition in mammography images. *Diagnostics*, 13(9):1618, 2023.
130. Dezső Ribli, Anna Horváth, Zsuzsa Unger, Péter Pollner, and István Csabai. Detecting and classifying lesions in mammograms with deep learning. *Scientific reports*, 8(1):4165, 2018.
131. Rocky Mountain Cancer Centers. Understanding Breast Cancer Types | Colorado’s Breast Cancer Specialists — rockymountaincancercenters.com, n.d. Accessed December 22, 2023.
132. Nabilah Ruza, Saiful Izzuan Hussain, Siti Kamariah Che Mohamed, and Mohd Hafiz Arzmi. Early detection of breast cancer in mammograms using the lightweight modification of effi- cientnet b3. *Métodos numéricos para cálculo y diseño en ingeniería: Revista internacional*, 39(3):1–11, 2023.
133. Abeer Saber, Abdelazim G Hussien, Wael A Awad, Amena Mahmoud, and Alaa Allakany. Adapting the pre-trained convolutional neural networks to improve the anomaly detection and classification in mammographic images. *Scientific Reports*, 13(1):14877, 2023.
134. Abeer Saber, Mohamed Sakr, Osama M Abo-Seida, Arabi Keshk, and Huiling Chen. A novel deep-learning model for automatic detection and classification of breast cancer using the transfer-learning technique. *IEEE Access*, 9:71194–71209, 2021.
135. Nasibeh Saffari, Hatem A Rashwan, Mohamed Abdel-Nasser, Vivek Kumar Singh, Meritxell Arenas, Eleni Mangina, Blas Herrera, and Domenec Puig. Fully automated breast density segmentation and classification using deep learning. *Diagnostics*, 10(11):988, 2020.
136. Unaiza Sajid, Rizwan Ahmed Khan, Shahid Munir Shah, and Sheeraz Arif. Breast cancer classification using deep learned features boosted with handcrafted features. *Biomedical Signal Processing and Control*, 86:105353, 2023.
137. NK Sakthivel, S Subasree, Pachhaiammal Alias Priya M, and Amit Kumar Tyagi. Breast lesion identification and categorization using mammography screening based on combined convolutional recursive neural network framework with parameters optimized using multi- objective seagull optimization algorithm. *Concurrency and Computation: Practice and Experience*, 34(28):e7348, 2022.
138. NK Sakthivel, S Subasree, Shaveta Malik, and Amit Kumar Tyagi. A wrapper based fea- ture extraction framework based on alexnet deep convolutional neural network parameters optimized using gradient-based optimizer for mammogram images. *Concurrency and Com- putation: Practice and Experience*, 34(18):e7008, 2022.
139. Wessam M Salama and Moustafa H Aly. Deep learning in mammography images segmentation and classification: Automated cnn approach. *Alexandria Engineering Journal*, 60(5):4701– 4709, 2021.
140. Wessam M Salama, Azza M Elbagoury, and Moustafa H Aly. Novel breast cancer classification framework based on deep learning. *IET Image Processing*, 14(13):3254–3259, 2020.
141. Nagwan Abdel Samee, Amel A Alhussan, Vidan Fathi Ghoneim, Ghada Atteia, Reem Alkan- hel, Mugahed A Al-Antari, and Yasser M Kadah. A hybrid deep transfer learning of cnn-based lr-pca for breast lesion diagnosis via medical breast mammograms. *Sensors*, 22(13):4938, 2022.
142. Nagwan Abdel Samee, Ghada Atteia, Souham Meshoul, Mugahed A Al-antari, and Yasser M Kadah. Deep learning cascaded feature selection framework for breast cancer classification: Hybrid cnn with univariate-based approach. *Mathematics*, 10(19):3631, 2022.
143. SR Sannasi Chakravarthy, N Bharanidharan, and Harikumar Rajaguru. Multi-deep cnn based experimentations for early diagnosis of breast cancer. *IETE Journal of Research*, pages 1–16, 2022.
144. SR Sannasi Chakravarthy and Harikumar Rajaguru. Deep-features with bayesian optimized classifiers for the breast cancer diagnosis. *International Journal of Imaging Systems and Technology*, 31(4):1861–1881, 2021.
145. D Saranyaraj, M Manikandan, and S Maheswari. A deep convolutional neural network for the early detection of breast carcinoma with respect to hyper-parameter tuning. *Multimedia Tools and Applications*, 79(15-16):11013–11038, 2020.
146. T SenthilPrakash, G Kannan, Salini Prabhakaran, and Bhagirath Parshuram Prajapati. Deep convolutional spiking neural network fostered automatic detection and classification of breast cancer from mammography images. *Research on Biomedical Engineering*, pages 1–9, 2023.
147. Zijun Sha, Lin Hu, and Babak Daneshvar Rouyendegh. Deep learning and optimization algorithms for automatic breast cancer detection. *International Journal of Imaging Systems and Technology*, 30(2):495–506, 2020.
148. MC Shanker and M Vadivel. Hybrid transfer learning of mammogram images for screening of micro-calcifications. *SSRG International Journal of Electrical and Electronics Engineering*, 9(8):40–47, 2022.
149. MC Shanker and M Vadivel. Micro-calcification classification analysis in mammogram images with aid of hybrid technique analysis. *Wireless Personal Communications*, 128(2):1287–1307, 2023.
150. Li Shen, Laurie R Margolies, Joseph H Rothstein, Eugene Fluder, Russell McBride, and Weiva Sieh. Deep learning to improve breast cancer detection on screening mammography. *Scientific reports*, 9(1):12495, 2019.
151. Rongbo Shen, Kezhou Yan, Kuan Tian, Cheng Jiang, and Ke Zhou. Breast mass detection from the digitized x-ray mammograms based on the combination of deep active learning and self-paced learning. *Future Generation Computer Systems*, 101:668–679, 2019.
152. Tianyu Shen, Jiangong Wang, Chao Gou, and Fei-Yue Wang. Hierarchical fused model with deep learning and type-2 fuzzy learning for breast cancer diagnosis. *IEEE Transactions on Fuzzy Systems*, 28(12):3204–3218, 2020.
153. Yiqiu Shen, Nan Wu, Jason Phang, Jungkyu Park, Kangning Liu, Sudarshini Tyagi, Laura Heacock, S Gene Kim, Linda Moy, Kyunghyun Cho, et al. An interpretable classifier for high- resolution breast cancer screening images utilizing weakly supervised localization. *Medical image analysis*, 68:101908, 2021.
154. Ekta Shivhare and Vineeta Saxena. Optimized generative adversarial network based breast cancer diagnosis with wavelet and texture features. *Multimedia Systems*, 28(5):1639–1655, 2022.
155. Jaspreet Singh et al. Mammography image abnormalities detection and classification by deep learning with extreme learner. *International Journal of Advanced Computer Science and Applications*, 14(3), 2023.
156. Laxman Singh and Altaf Alam. An efficient hybrid methodology for an early detection of breast cancer in digital mammograms. *Journal of Ambient Intelligence and Humanized Computing*, pages 1–24, 2022.
157. V Sivakrithika and K Dinakaran. Subclass based parallel learning neural network for classifi- cation of masses in mammograms. *Design Automation for Embedded Systems*, 22(1-2):65–79, 2018.
158. J Sivamurugan and G Sureshkumar. Applying dual models on optimized lstm with u-net segmentation for breast cancer diagnosis using mammogram images. *Artificial Intelligence in Medicine*, 143:102626, 2023.
159. Robin Smithuis and Frank Smithuis. Benign calcifications, n.d. Accessed December 22, 2023.
160. Alex Snyder. Invasive ductal carcinoma, Apr 2022. Accessed December 23, 2023.
161. Hossein Soleimani and Oleg V Michailovich. On segmentation of pectoral muscle in digital mammograms by means of deep learning. *IEEE Access*, 8:204173–204182, 2020.
162. Hama Soltani, Mohamed Amroune, Issam Bendib, Mohamed-Yassine Haouam, Elhadj Benkhelifa, and Muhammad Moazam Fraz. Breast lesions segmentation and classification in a two-stage process based on mask-rcnn and transfer learning. *Multimedia Tools and Applications*, pages 1–18, 2023.
163. Runyu Song, Taoying Li, and Yan Wang. Mammographic classification based on xgboost and dcnn with multi features. *IEEE Access*, 8:75011–75021, 2020.
164. Chatsuda Songsaeng, Piyanoot Woodtichartpreecha, and Sitthichok Chaichulee. Multi-scale convolutional neural networks for classification of digital mammograms with breast calcifica- tions. *IEEE Access*, 9:114741–114753, 2021.
165. Khaoula Belhaj Soulami, Naima Kaabouch, and Mohamed Nabil Saidi. Breast cancer: Clas- sification of suspicious regions in digital mammograms based on capsule network. *Biomedical Signal Processing and Control*, 76:103696, 2022.
166. Khaoula Belhaj Soulami, Naima Kaabouch, Mohamed Nabil Saidi, and Ahmed Tamtaoui. Breast cancer: One-stage automated detection, segmentation, and classification of digital mammograms using unet model based-semantic segmentation. *Biomedical Signal Processing and Control*, 66:102481, 2021.
167. V Sridevi and J Abdul Samath. A combined deep cnn-lasso regression feature fusion and clas- sification of mlo and cc view mammogram image. *International Journal of System Assurance Engineering and Management*, pages 1–11, 2023.
168. Alexandra Stanislavsky. Neurofibromatosis of the breast: Radiology case, Nov 2022. Accessed December 20, 2023.
169. Yongye Su, Qian Liu, Wentao Xie, and Pingzhao Hu. Yolo-logo: A transformer-based yolo segmentation model for breast mass detection and segmentation in digital mammograms. *Computer Methods and Programs in Biomedicine*, 221:106903, 2022.
170. Yong Joon Suh, Jaewon Jung, and Bum-Joo Cho. Automated breast cancer detection in digital mammograms of various densities via deep learning. *Journal of personalized medicine*, 10(4):211, 2020.
171. Hui Sun, Cheng Li, Boqiang Liu, Zaiyi Liu, Meiyun Wang, Hairong Zheng, David Dagan Feng, and Shanshan Wang. Aunet: attention-guided dense-upsampling networks for breast mass segmentation in whole mammograms. *Physics in Medicine & Biology*, 65(5):055005, 2020.
172. Lilei Sun, Huijie Sun, Junqian Wang, Shuai Wu, Yong Zhao, and Yong Xu. Breast mass detection in mammography based on image template matching and cnn. *Sensors*, 21(8):2855, 2021.
173. Lilei Sun, Jie Wen, Junqian Wang, Yong Zhao, Bob Zhang, Jian Wu, and Yong Xu. Two-view attention-guided convolutional neural network for mammographic image classification. *CAAI Transactions on Intelligence Technology*, 8(2):453–467, 2023.
174. Yeheng Sun and Yule Ji. Aaws-net: Anatomy-aware weakly-supervised learning network for breast mass segmentation. *PloS one*, 16(8):e0256830, 2021.
175. Yingshi Sun, Yuhong Qu, Dong Wang, Yi Li, Lin Ye, Jingbo Du, Bing Xu, Baoqing Li, Xiaoting Li, Kexin Zhang, et al. Deep learning model improves radiologists’ performance in detection and classification of breast lesions. *Chinese Journal of Cancer Research*, 33(6):682, 2021.
176. Y Nguyen Tan, Vo Phuc Tinh, Pham Duc Lam, Nguyen Hoang Nam, and Tran Anh Khoa. A transfer learning approach to breast cancer classification in a federated learning framework. *IEEE Access*, 11:27462–27476, 2023.
177. Mickael Tardy and Diana Mateus. Looking for abnormalities in mammograms with self-and weakly supervised reconstruction. *IEEE Transactions on Medical Imaging*, 40(10):2711– 2722, 2021.
178. Nasrin Tavakoli, Maryam Karimi, Alireza Norouzi, Nader Karimi, Shadrokh Samavi, and SM Reza Soroushmehr. Detection of abnormalities in mammograms using deep features. *Journal of Ambient Intelligence and Humanized Computing*, pages 1–13, 2019.
179. Selvakumar Thirumalaisamy, Kamaleshwar Thangavilou, Hariharan Rajadurai, Oumaima Saidani, Nazik Alturki, Sandeep kumar Mathivanan, Prabhu Jayagopal, and Saikat Gochhait. Breast cancer classification using synthesized deep learning model with metaheuristic opti- mization algorithm. *Diagnostics*, 13(18):2925, Sep 2023.
180. Nagalakshmi Thirunavukkarasu, Govindarajan Muthukumarasamy, and Ramalingam Mu- rugesan. Breast cancer detection in mammogram images by mapreduce based deep con- volutional neural networks. *International Journal of Intelligent Engineering & Systems*, 15(1), 2022.
181. VM Tiryaki and V Kaplanoğlu. Deep learning-based multi-label tissue segmentation and density assessment from mammograms. *IRBM*, 43(6):538–548, 2022.
182. Volkan Müjdat Tiryaki. Mass segmentation and classification from film mammograms using cascaded deep transfer learning. *Biomedical Signal Processing and Control*, 84:104819, 2023.
183. Kuen-Jang Tsai, Mei-Chun Chou, Hao-Ming Li, Shin-Tso Liu, Jung-Hsiu Hsu, Wei-Cheng Yeh, Chao-Ming Hung, Cheng-Yu Yeh, and Shaw-Hwa Hwang. A high-performance deep neural network model for bi-rads classification of screening mammography. *Sensors*, 22(3):1160, 2022.
184. Lazaros Tsochatzidis, Panagiota Koutla, Lena Costaridou, and Ioannis Pratikakis. Integrat- ing segmentation information into cnn for breast cancer diagnosis of mammographic masses. *Computer Methods and Programs in Biomedicine*, 200:105913, 2021.
185. Ioannis N Tzortzis, Agapi Davradou, Ioannis Rallis, Maria Kaselimi, Konstantinos Makanta- sis, Anastasios Doulamis, and Nikolaos Doulamis. Tensor-based learning for detecting abnor- malities on digital mammograms. *Diagnostics*, 12(10):2389, 2022.
186. Daiju Ueda, Akira Yamamoto, Naoyoshi Onoda, Tsutomu Takashima, Satoru Noda, Shinichiro Kashiwagi, Tamami Morisaki, Shinya Fukumoto, Masatsugu Shiba, Mina Morimura, et al. Development and validation of a deep learning model for detection of breast cancers in mam- mography from multi-institutional datasets. *PLoS One*, 17(3):e0265751, 2022.
187. Venugopal Ulagamuthalvi, Govindarajan Kulanthaivel, A Balasundaram, and Arun Kumar Sivaraman. Breast mammogram analysis and classification using deep convolution neural network. *Comput. Syst. Sci. Eng.*, 43(1):275–289, 2022.
188. Hosna Asma Ull, Il Dong Yun, and Bo La Yun. Regression to classification: Ordinal prediction of calcified vessels using customized resnet50. *IEEE Access*, 2023.
189. I Valencia-Hernandez, Hayde Peregrina-Barreto, CA Reyes-Garcia, and GC Lopez-Armas. Density map and fuzzy classification for breast density by using bi-rads. *Computer Methods and Programs in Biomedicine*, 200:105825, 2021.
190. Gabriele Valvano, Gianmarco Santini, Nicola Martini, Andrea Ripoli, Chiara Iacconi, Dante Chiappino, Daniele Della Latta, et al. Convolutional neural networks for the segmentation of microcalcification in mammography imaging. *Journal of healthcare engineering*, 2019, 2019.
191. Chong Wang, Yuanhong Chen, Fengbei Liu, Michael Elliott, Chun Fung Kwok, Carlos Peña- Solorzano, Helen Frazer, Davis James McCarthy, and Gustavo Carneiro. An interpretable and accurate deep-learning diagnosis framework modelled with fully and semi-supervised re- ciprocal learning. *IEEE Transactions on Medical Imaging*, 2023.
192. Juan Wang and Yongyi Yang. A context-sensitive deep learning approach for microcalcifica- tion detection in mammograms. *Pattern recognition*, 78:12–22, 2018.
193. Yan Wang, Yangqin Feng, Lei Zhang, Zizhou Wang, Qing Lv, and Zhang Yi. Deep adversarial domain adaptation for breast cancer screening from mammograms. *Medical image analysis*, 73:102147, 2021.
194. Yan Wang, Zizhou Wang, Yangqin Feng, and Lei Zhang. Wdccnet: Weighted double-classifier constraint neural network for mammographic image classification. *IEEE Transactions on Medical Imaging*, 41(3):559–570, 2021.
195. Yan Wang, Lei Zhang, Xin Shu, Yangqin Feng, Zhang Yi, and Qing Lv. Feature-sensitive deep convolutional neural network for multi-instance breast cancer detection. *IEEE/ACM Transactions on Computational Biology and Bioinformatics*, 19(4):2241–2251, 2021.
196. Yiming Wang, Yunliang Qi, Chunbo Xu, Meng Lou, and Yide Ma. Learning multi-frequency features in convolutional network for mammography classification. *Medical & Biological Engineering & Computing*, 60(7):2051–2062, 2022.
197. Yuehang Wang, Shengsheng Wang, Juan Chen, and Chun Wu. Whole mammographic mass segmentation using attention mechanism and multiscale pooling adversarial network. *Journal of Medical Imaging*, 7(5):054503–054503, 2020.
198. Zhiqiong Wang, Mo Li, Huaxia Wang, Hanyu Jiang, Yudong Yao, Hao Zhang, and Junchang Xin. Breast cancer detection using extreme learning machine based on feature fusion with cnn deep features. *IEEE Access*, 7:105146–105158, 2019.
199. Maria Wimmer, Gert Sluiter, David Major, Dimitrios Lenis, Astrid Berg, Theresa Neubauer, and Katja Bühler. Multi-task fusion for improving mammography screening data classifica- tion. *IEEE Transactions on Medical Imaging*, 41(4):937–950, 2021.
200. Nan Wu, Jason Phang, Jungkyu Park, Yiqiu Shen, Zhe Huang, Masha Zorin, Stanisław Jastrzębski, Thibault Févry, Joe Katsnelson, Eric Kim, et al. Deep neural networks improve radiologists’ performance in breast cancer screening. *IEEE transactions on medical imaging*, 39(4):1184–1194, 2019.
201. Lili Xia, Jianpeng An, Chao Ma, Hongjun Hou, Yanpeng Hou, Linyang Cui, Xuheng Jiang, Wanqing Li, and Zhongke Gao. Neural network model based on global and local features for multi-view mammogram classification. *Neurocomputing*, 536:21–29, 2023.
202. Shengzhou Xu, Ehsan Adeli, Jie-Zhi Cheng, Lei Xiang, Yang Li, Seong-Whan Lee, and Ding- gang Shen. Mammographic mass segmentation using multichannel and multiscale fully convo- lutional networks. *International Journal of Imaging Systems and Technology*, 30(4):1095– 1107, 2020.
203. Asumi Yamazaki and Takayuki Ishida. Two-view mammogram synthesis from single-view data using generative adversarial networks. *Applied Sciences*, 12(23):12206, 2022.
204. Yutong Yan, Pierre-Henri Conze, Mathieu Lamard, Gwenolé Quellec, Béatrice Cochener, and Gouenou Coatrieux. Towards improved breast mass detection using dual-view mammogram matching. *Medical image analysis*, 71:102083, 2021.
205. Yutong Yan, Pierre-Henri Conze, Gwenolé Quellec, Mathieu Lamard, Béatrice Cochener, and Gouenou Coatrieux. Two-stage multi-scale breast mass segmentation for full mammogram analysis without user intervention. *Biocybernetics and Biomedical Engineering*, 41(2):746– 757, 2021.
206. Melissa Min-Szu Yao, Hao Du, Mikael Hartman, Wing P Chan, and Mengling Feng. End- to-end calcification distribution pattern recognition for mammograms: An interpretable ap- proach with gnn. *Diagnostics*, 12(6):1376, 2022.
207. Joshua Yap. Sclerosing adenosis: Radiology reference article, Feb 2023. Accessed December 20, 2023.
208. Xiang Yu, Cheng Kang, David S Guttery, Seifedine Kadry, Yang Chen, and Yu-Dong Zhang. Resnet-scda-50 for breast abnormality classification. *IEEE/ACM transactions on computa- tional biology and bioinformatics*, 18(1):94–102, 2020.
209. Xiang Yu, Zeyu Ren, David S Guttery, and Yu-Dong Zhang. Df-drvfl: A novel deep feature based classifier for breast mass classification. *Multimedia Tools and Applications*, pages 1–30, 2023.
210. Xiang Yu, Shui-Hua Wang, Juan Manuel Górriz, Xian-Wei Jiang, David S Guttery, and Yu- Dong Zhang. Pemnet for pectoral muscle segmentation. *Biology*, 11(1):134, 2022.
211. Xiang Yu, Shui-Hua Wang, and Yu-Dong Zhang. Multiple-level thresholding for breast mass detection. *Journal of King Saud University-Computer and Information Sciences*, 35(1):115– 130, 2023.
212. Xiang Yu, Ziquan Zhu, Yoav Alon, David S Guttery, and Yudong Zhang. Gfnet: A deep learning framework for breast mass detection. *Electronics*, 12(7):1583, 2023.
213. Ayşe Aydın Yurdusev, Kemal Adem, and Mahmut Hekim. Detection and classification of microcalcifications in mammograms images using difference filter and yolov4 deep learning model. *Biomedical Signal Processing and Control*, 80:104360, 2023.
214. Saliha Zahoor, Umar Shoaib, and Ikram Ullah Lali. Breast cancer mammograms classification using deep neural network and entropy-controlled whale optimization algorithm. *Diagnostics*, 12(2):557, 2022.
215. Felipe André Zeiser, Cristiano André da Costa, Tiago Zonta, Nuno MC Marques, Adriana Vial Roehe, Marcelo Moreno, and Rodrigo da Rosa Righi. Segmentation of masses on mammo- grams using data augmentation and deep learning. *Journal of digital imaging*, 33:858–868, 2020.
216. Chen Zhang, Jumin Zhao, Jing Niu, and Dengao Li. New convolutional neural network model for screening and diagnosis of mammograms. *PLoS One*, 15(8):e0237674, 2020.
217. Xinsheng Zhang and Zhe Wang. A microcalcification cluster detection method based on deep learning and multi-scale feature fusion. *The Journal of Supercomputing*, 75:5808–5830, 2019.
218. Xinyu Zhang, Cuixia Liang, Dong Zeng, Xiaocong Jiang, Rikui Zhong, Yuhong Lan, Jianhua Ma, and Li Bai. Pattern classification for breast lesion on ffdm by integration of radiomics and deep features. *Computerized Medical Imaging and Graphics*, 90:101922, 2021.
219. Yu-Dong Zhang, Chichun Pan, Xianqing Chen, and Fubin Wang. Abnormal breast identifi- cation by nine-layer convolutional neural network with parametric rectified linear unit and rank-based stochastic pooling. *Journal of computational science*, 27:57–68, 2018.
220. Yu-Dong Zhang, Suresh Chandra Satapathy, David S Guttery, Juan Manuel Górriz, and Shui-Hua Wang. Improved breast cancer classification through combining graph convolu- tional network and convolutional neural network. *Information Processing & Management*, 58(2):102439, 2021.
221. Jianhui Zhao, Tianquan Chen, and Bo Cai. A computer-aided diagnostic system for mam- mograms based on yolov3. *Multimedia Tools and Applications*, pages 1–25, 2022.
222. Kuochen Zhou, Wei Li, and Dazhe Zhao. Deep learning-based breast region extraction of mammographic images combining pre-processing methods and semantic segmentation sup- ported by deeplab v3+. *Technology and Health Care*, 30(S1):173–190, 2022.
223. Yuanpin Zhou, Jun Wei, Dongmei Wu, and Yaqin Zhang. Generating full-field digital mammo- gram from digitized screen-film mammogram for breast cancer screening with high-resolution generative adversarial network. *Frontiers in Oncology*, 12:868257, 2022.
224. *Cancer Today*. (n.d.). Retrieved January 27, 2025, from https://gco.iarc.fr/today/en
